# Molecular basis for the allosteric activation mechanism of the heterodimeric imidazole glycerol phosphate synthase complex

**DOI:** 10.1038/s41467-021-22968-6

**Published:** 2021-05-12

**Authors:** Jan Philip Wurm, Sihyun Sung, Andrea Christa Kneuttinger, Enrico Hupfeld, Reinhard Sterner, Matthias Wilmanns, Remco Sprangers

**Affiliations:** 1 Institute of Biophysics and Physical Biochemistry, Regensburg Center for Biochemistry, University of Regensburg, Regensburg, Germany; 2 European Molecular Biology Laboratory, Hamburg Unit, Hamburg, Germany; 3 University Hamburg Clinical Center Hamburg-Eppendorf, Hamburg, Germany

**Keywords:** Enzyme mechanisms, Solution-state NMR, X-ray crystallography

## Abstract

Imidazole glycerol phosphate synthase (HisFH) is a heterodimeric bienzyme complex operating at a central branch point of metabolism. HisFH is responsible for the HisH-catalyzed hydrolysis of glutamine to glutamate and ammonia, which is then used for a cyclase reaction by HisF. The HisFH complex is allosterically regulated but the underlying mechanism is not well understood. Here, we elucidate the molecular basis of the long range, allosteric activation of HisFH. We establish that the catalytically active HisFH conformation is only formed when the substrates of both HisH and HisF are bound. We show that in this conformation an oxyanion hole in the HisH active site is established, which rationalizes the observed 4500-fold allosteric activation compared to the inactive conformation. In solution, the inactive and active conformations are in a dynamic equilibrium and the HisFH turnover rates correlate with the population of the active conformation, which is in accordance with the ensemble model of allostery.

## Abstract (web summary)

The allosteric regulation of the bienzyme complex imidazole glycerol phosphate synthase (HisFH) remains to be elucidated. Here, the authors provide structural insights into the dynamic allosteric mechanism by which ligand binding to the cyclase and glutaminase active sites of HisFH regulate enzyme activation.

## Introduction

Allostery, the communication of signals between distal sites in (bio)-macromolecules, is a ubiquitous biochemical phenomenon and involved in the regulation of basically every cellular process. When ligand binding to one site influences a remote site, it is possible to describe the thermodynamic basis of allostery using an ensemble model^1^. This model states that binding of one ligand shifts the ensemble of macromolecular states toward a conformation with altered affinity or activity with respect to a second ligand. A major challenge in the study of allosteric processes lies in the quantification and structural description of these ensembles as the populations of high energy states are often too small to be experimentally accessible. The situation is even more complex under non-equilibrium conditions, a situation inevitably encountered in biology, where kinetic effects can dominate over thermodynamics. A striking example is the mnemonical effect, where slow conformational transitions take place in the enzyme upon ligand binding^2,3^. A comprehensive understanding of allosteric processes in enzymes thus requires a complete description of all structural states involved, their populations, dynamics and interconversion rates.

**Fig. 1:**
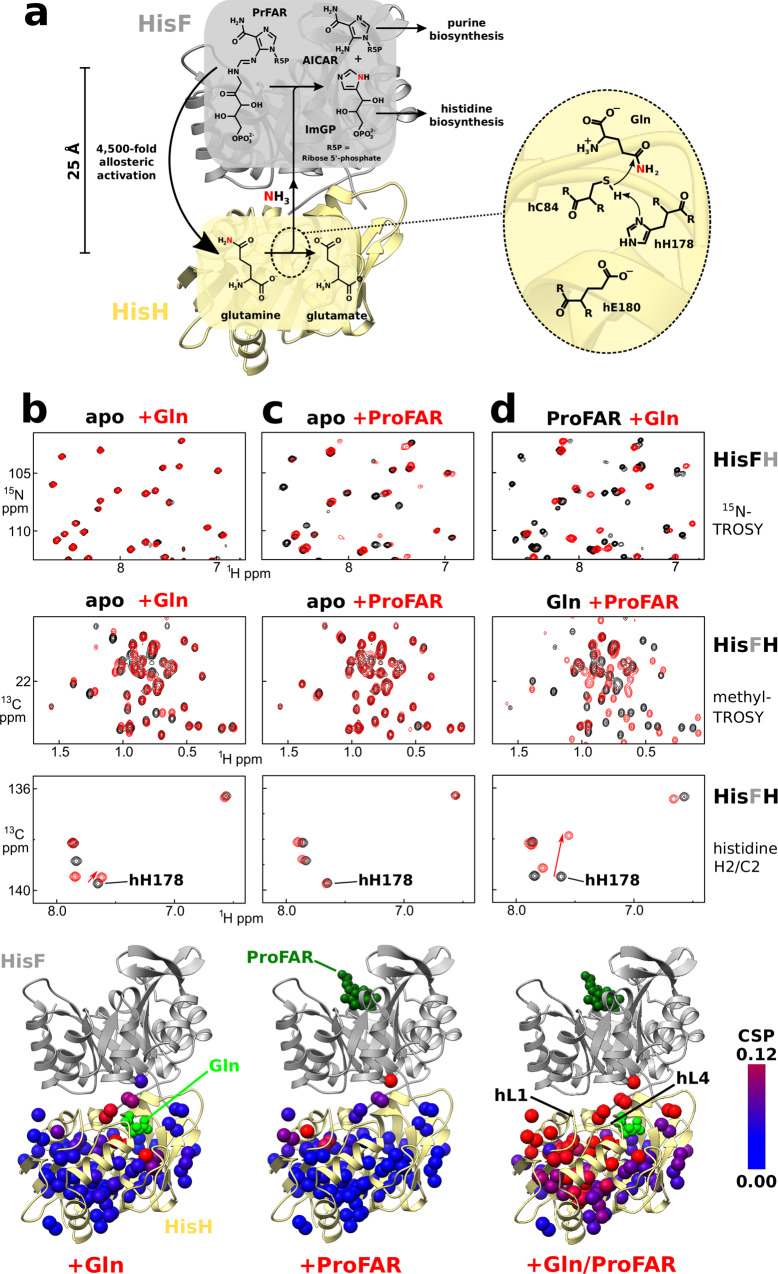
Formation of the active HisFH conformation requires both substrates. **a** Reaction catalyzed by the HisFH complex, the glutaminase activity of HisH is dramatically increased by PrFAR binding to HisF. Right: catalytic triad of HisH and the initial step of the glutaminase reaction. **b**–**d** NMR titration experiments reveal that both substrates are necessary for the formation of the active conformation of HisF and HisH. The top and middle spectra are recorded on a HisFH–hC84S complex where signals of HisF backbone amides are visible in ^1^H-^15^N TROSY spectra and HisH methyl group signals are visible in methyl-TROSY spectra (the invisible subunit is indicated in gray on the right). The bottom spectrum is recorded on a HisFH–hC84S complex that contains histidine-^13^C2/^1^H2 in HisH. In the top spectrum only the HisF subunit of the HisFH complex is visible, in the middle and bottom spectra only the HisH subunit of the HisFH complex is visible. **b** Spectra of the apo HisFH complex (black) and the HisFH complex in the presence of saturating amounts of Gln (red). Gln binding is only sensed in the vicinity of the HisH active site. Chemical shift perturbations (CSPs) upon Gln binding mapped on the HisH structure (PDBID: 1GPW). **c** As in (**b**), but comparing the apo (black) HisFH and the ProFAR-bound HisFH complex (red). ProFAR binding to HisF leads to large CSPs in HisF, but HisH is only influenced in the vicinity of the HisF–HisH interface. No CSPs are observed for the catalytic histidine, that is 25 Å remote to the HisF active site. **d** As in (**b**, **c**), but comparing HisFH spectra in the presence of both ligands (red) to spectra in which only one ligand is bound (black; corresponding to the red spectra in **b**, **c**). CSPs in HisH are dramatically increased when ProFAR binds in the presence of Gln (middle and bottom spectra, compare to **c**). Similarly, Gln binding is communicated to HisF when ProFAR is present as many CSPs are observed in HisF (top spectrum, compare to **b**).

The imidazole glycerol phosphate synthase from *Thermotoga maritima* forms a heterodimeric bienzyme complex consisting of the two subunits HisF and HisH (HisFH). HisFH belongs to the class I of glutamine amidotransferases (GATs) that play a central role in metabolism as they incorporate nitrogen derived from glutamine into a variety of metabolites^4–8^. The HisFH complex catalyzes the synthesis of the histidine precursor imidazole glycerol phosphate (ImGP) and the purine precursor 5-aminoimidazole-4-carboxamide ribonucleotide (AICAR) from N′-[(5′-phosphoribulosyl) formimino]-5-aminoimidazole-4-carboxamide ribonucleotide (PrFAR) and glutamine (Gln)^9,10^ (Fig. 1a). This reaction takes place in two main steps and starts with the hydrolysis of Gln to glutamate (Glu) and ammonia in the glutaminase subunit HisH. The ammonia then migrates over 25 Å from the HisH active site through a central tunnel across the beta-strand barrel of HisF to the HisF cyclase active site at the opposite face of the barrel^11^. There it reacts with PrFAR to produce ImGP and AICAR. In the absence of a HisF ligand the glutaminase activity of HisFH is only basal, which prevents the wasteful turnover of Gln by HisFH. Binding of PrFAR to the HisF active site allosterically increases the turnover number (*k*_cat_) of the glutaminase reaction in HisH by ~4500-fold^9,12^. HisFH thereby achieves a very tight coupling between the two reactions which ensures that every ammonia molecule that is produced by HisH is utilized in the HisF dependent reaction^13^.

The active site of HisH consists of a catalytic triad composed of a cysteine (hC84), a histidine (hH178) and a glutamate (hE180) (h and f indicate residues/secondary structure elements from HisH or HisF, respectively; Fig. 1a)^11^. In analogy to the well-studied papain-like cysteine proteases, the cysteine acts as a nucleophile and attacks the Gln amide carbon thereby forming a thioester via an oxyanion intermediate (Supplementary Fig. 1), which leads to the release of ammonia^8^. In the final step the thioester is hydrolyzed, again via an oxyanion intermediate. Other class I GATs possess a preformed oxyanion hole^14–19^, which consists of two backbone amide protons that stabilize the oxyanion intermediates during catalysis. In HisH and in the homologous HIS7 protein from yeast where the cyclase and the glutaminase domains are fused, the oxyanion hole is not preformed, as one of the amides groups (hV51) is flipped^11,20^. As a result, the amide proton points away from the catalytic site and the carbonyl oxygen of hV51 blocks the amide proton hL85 in the oxyanion hole (see below), which explains why the catalytic activity of HisH in the absence of an activating event is very low. It has been suggested^21^ that the allosteric activation of the glutaminase reaction entails the formation of an oxyanion hole in HisH similar to the ones observed in other class I GATs.

Substrate-dependent allosteric activation of the glutaminase reaction is observed in most GATs and HisFH as well as HIS7 have been used as model systems for the study of this mechanism. So far, available crystal structures show minimal structural changes in the glutaminase or the cyclase active sites upon binding of their respective substrate. Even when both binding sites are occupied, long-range conformational changes that might explain the allosteric effect have so far not been observed^21^. On the other hand, molecular dynamics (MD) simulations have revealed that PrFAR binding leads to changes in correlated ns timescale motions in HisF and influences fast inter-subunit motions between HisF and HisH^22–26^. Recent results suggest that the inter protein angle between HisF and HisH is an important determinant for the catalytic activity as the structure of a HisFH complex with increased basal activity shows a more closed inter-subunit orientation^12^. NMR measurements show that binding of ligands to HisF results in increased dynamics that extend from the HisF active site to the HisF–HisH interface and it has been proposed that these dynamics are part of the allosteric coupling between the two enzymes^27,28^. Interestingly, it has been observed that high concentrations of the products of the cyclase reaction (ImGP^9^ and AICAR^29^) can also stimulate the glutaminase activity and a correlation between the degree of stimulation of HisH and dynamics in HisF was found^28^. This correlation was further supported by the identification of mutations that reduce HisF dynamics and that interfere with the allosteric HisFH communication^30^. Taken together, previous work identified the part of HisF that encompasses fα1–3/fβ1–3 (α-helices 1–3 as well as β-strands 1–3 in HisF) and the connecting loops as a hot spot for the transmittance of the allosteric signal from HisF to HisH^9,12,26,28^. Importantly, this region includes fL1 (β1α1 loop of HisF), which displays varying conformations between different crystal structures^11^ and that has been proposed to interact with the PrFAR molecule in the HisF active site. The structure of the active enzyme complex and the mechanism behind the impressive allosteric activation remain, however, elusive.

In this work, we show how dual ligand binding to the cyclase and glutaminase active sites induces a large number of structural changes in HisFH that ultimately result in the formation of a fully active conformation of the complex. In the active conformation, the cyclase active site is closed and the oxyanion hole in the glutaminase active site is formed. In solution, the active conformation is in a dynamic equilibrium with the inactive form of the enzyme, even when both active sites are fully occupied, and the activity of the complex is directly determined by the population of the active conformation. Our data thus reveal a detailed and dynamic picture that describes the mechanism of the allosteric activation of a central metabolic enzyme complex.

## Results

### The simultaneous interaction with both substrates causes large conformational changes in HisFH

In order to delineate the allosteric activation pathway that connects the two active sites of the HisFH complex, we performed NMR titration experiments. To that end, we prepared highly deuterated enzyme complexes in which HisF is ^1^H–^15^N labeled and HisH is ^1^H–^13^C labeled in all methyl groups except for threonine^31,32^ (Supplementary Fig. 2). This labeling scheme allows us to independently detect interactions and structural changes in both subunits of the complex within the same sample. To monitor conformational changes of the catalytic histidine hH178 we also prepared deuterated HisFH complexes, in which the HisH histidines are labeled at the C2/H2 position (Supplementary Fig. 3). As the PrFAR substrate of HisFH is too unstable for NMR measurements we use as an analog the more stable precursor ProFAR (Supplementary Fig. 4) that shows a similar stimulation of the turnover number of the HisH glutaminase reaction^12^. To prevent rapid turnover of Gln during our experiments in the presence of both substrates (see below), we used a HisH mutant that displays strongly reduced glutaminase activity (hC84S) (Supplementary Fig. 5). Importantly, the wild type (WT) HisFH and the hC84S HisFH complex interact with Gln or ProFAR in the same way as very similar chemical shift perturbations (CSPs) are observed for both complexes (Supplementary Figs. 6–8).

In a number of NMR titration experiments, we find that Gln binding to the HisFH complex leads to a few small CSPs in HisF (Fig. 1b, top panel). These CSPs localize to residues at the protein–protein interface (Supplementary Fig. 9), showing that Gln binding to the active site of HisH does not induce conformational changes within HisF. As expected, Gln binding does cause significant CSPs in the close vicinity of the known Gln binding site in HisH (Fig. 1b, middle and bottom panels, Supplementary Fig. 10), including hH178 in the active site. In a complementary experiment, we find that the addition of ProFAR to HisFH in the absence of Gln leads to large CSPs and strong peak broadening in HisF (Fig. 1c, top panel, Supplementary Figs. 6, 7). In previous studies similar broadening for HisF resonances upon interaction with other activating ligands has been observed^27,28^. This indicates that ligand binding induces dynamics in HisF on the μs-ms (microsecond to millisecond) timescale. At the same time, we observe only a few CSPs in HisH that all localize to the HisF–HisH interface (Fig. 1c, middle panel). Importantly, no CSPs are detected for the catalytic hH178 in the HisH active site upon ProFAR binding to HisF (Fig. 1c, bottom panel). In summary these NMR titration experiments reveal that occupation of a single active site in the HisFH complex does not lead to structural changes in the unoccupied other active site.

We reasoned that simultaneous occupation of both active sites might be required for the allosteric communication between HisF and HisH. We observe that this is indeed the case, as addition of ProFAR to the Gln saturated HisFH complex results in dramatically increased CSPs in HisH (Fig. 1d, middle panel, compare to the titration in the absence of ProFAR, Fig. 1b). These CSPs are clustered in the vicinity of the β1α1 loop in HisH (hL1) and the adjacent β3α2 loop (hL4), which is part of the HisH active site. This is independently confirmed by the large CSPs for the catalytic hH178 (Fig. 1d, bottom panel). Likewise, we also observe large CSPs in HisF when Gln is added to the ProFAR saturated HisFH complex (Fig. 1d, top panel). In summary, these data show that binding of both ligands is necessary for the formation of a novel conformation that we show below to be the catalytically active one. This new conformation is tolerant to the hC84S mutation in HisH as virtually identical results are obtained in a hC84A background (Supplementary Fig. 11) as well as for WT HisFH in combination with the covalent HisH inhibitor DON (a thioester mimic; Supplementary Fig. 12). Interestingly, even at saturating concentrations of ProFAR and Gln the active conformation of the enzyme is only populated to 80% (Supplementary Fig. 13 and see below), indicating that the inactive and active conformations are in a dynamic equilibrium with interconversion rates that are slow on the NMR timescale (<50 s^−1^).

### The structure of the allosterically activated conformation of the enzyme

**Fig. 2:**
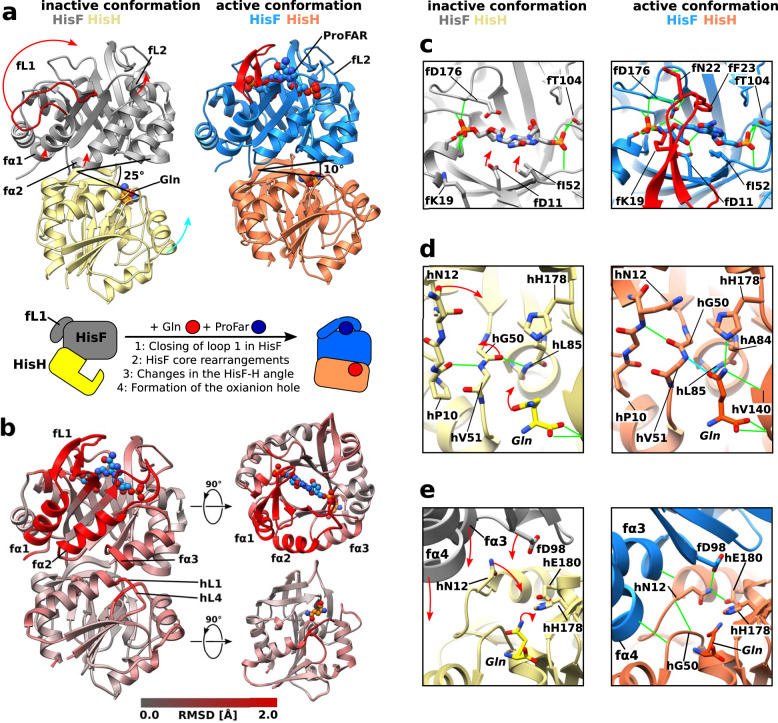
Structural changes in the HisFH complex upon allosteric activation. **a** Crystal structure of the HisFH complex (hC84A) in the inactive conformation (HisF in gray, HisH in yellow; PDBID: 7AC8, chains C/D) bound to Gln (left) and in the active conformation (HisF in marine, HisH in orange, chains E/F) bound to ProFAR and Gln (right). The largest structural differences between the two conformations are observed in HisF (red arrows). The reorientation of HisH relative to HisF is indicated by a cyan arrow and leads to a reduction of the angle between fF120, hW123 and hG52 (Cα atoms) from 25° to 10°. The major structural changes between the inactive and active conformations are indicated as cartoons. **b** The local backbone RMSD between the inactive and active conformations (PDBID 7AC8, chains C/D and E/F), after individual alignment of the proteins, mapped onto the structure of the active conformation. **c** Closeup of the HisF active site: Comparison between the inactive (left; PDBID 7AC8; chain A) and the active conformation (right; PDBID 7AC8, chain E). Important hydrogen bond interactions are shown in green. fL1 is shown in red. The compaction of the HisF active site in the active conformation is indicated by red arrows. **d** Oxyanion hole formation in the active conformation of HisH: The oxyanion hole is not formed in the inactive conformation (left; PDBID 7AC8, chain D). Red arrows indicate important conformational changes upon formation of the active conformation. The oxyanion hole (right; PDBID 7AC8, chain F) is formed by the rotation of the hG50/hV51 amide group. The side chain of the substrate Gln rotates into the oxyanion hole and forms hydrogen bonds to hG50 and hL85 (cyan). Other hydrogen bonds are shown in green. **e** The rearrangement of the HisF–HisH interface moves fα3/α4 and fD98 closer to the active site (red arrows). Upon transition from the inactive (left; PDBID 7AC8, chains C/D) into the active conformation (right; PDBID 7AC8, chains E/F) fD98 moves by 3.6 Å and interacts with hN12. Additional backbone hydrogen bonds are formed that connect hL4 and hα1 in HisH with fα4 and fα3 in HisF.

In order to visualize the structural changes that take place upon formation of the active conformation, we determined the crystal structure of HisFH in the presence of ProFAR and Gln. We used the inactive hC84A mutant for crystallization, which shows identical CSPs to the hC84S mutant (Supplementary Fig. 11) and solved the structure of the complex to a resolution of 2.06 Å. The asymmetric unit contains three HisFH complexes (Supplementary Table 1**;** Supplementary Fig. 14), all of which show electron density for Gln (Supplementary Fig. 15). Two of the complexes (chains A/B and C/D) adopt a conformation highly similar to previously published apo structures of the HisFH complex (Supplementary Fig. 16)^11,13^. We refer to this conformation as the inactive conformation of the complex (Fig. 2a). In one of these HisFH complexes in the inactive conformation (chains A/B) the HisF active site shows unambiguous electron density for a ProFAR molecule (Supplementary Fig. 15). In the second complex in the inactive conformation (chain C/D) electron density is located at the two sites equivalent to the phosphate groups of the ProFAR molecule in chain A. This density likely originates from a less well ordered ProFAR molecule and those sites were modeled as a phosphate ion and a solvent molecule (Supplementary Fig. 15). In these two complexes (chains A/B and C/D) substrate binding only induced minute conformational changes in the vicinity of the HisF ProFAR binding site (Supplementary Fig. 16), a situation that was also observed for HIS7 from yeast in complex with PrFAR^21^. In contrast, the third HisFH complex (chains E/F) in our crystal shows electron density for both ligands (Supplementary Fig. 15) and large, long range conformational changes that extend between both active sites (Fig. 2a). This conformation has not been observed before and we refer to this structure as the active conformation of HisFH (see also below).

We noticed four significant structural differences upon conversion from the inactive to the active conformation. First, the β1α1 loop (fL1) in HisF moves by 25 Å from a position close to helix fα1 to a state, in which it covers the HisF active site (Fig. 2a, red arrow). Accordingly, this large conformational change is emphasized by a large root-mean-square deviation (RMSD) value comparing the inactive and active conformation (Fig. 2b). In the closed state fL1 forms a short beta sheet and several highly conserved residues in the loop make extensive contacts with ProFAR and the HisF active site residues (Fig. 2c; Supplementary Fig. 16): fK19 forms a salt bridge with fD176 and the phosphate of ProFAR, the aromatic side chain of fF23 forms a π-stacking interaction with the imidazole ring of ProFAR and fN22 forms hydrogen bonds to the backbone carbonyls of fI173/fD174/fD176. In addition, the β2α2 loop (fL2) moves by 5 Å to contact the aromatic ring of ProFAR. In particular, the conserved residues fI52 and fS55 stack against the aromatic ring and form a hydrogen bond to the ProFAR phosphate, respectively. There is also a 2–3 Å compaction of the ProFAR binding site caused by small movements of fβ1/2, which positions the catalytically essential fD11^9^ closer to ProFAR. Second, with the transition into the active conformation helices fα1 and fα2 move away from HisH by 2–3 Å which leads to a rearrangement of the HisF hydrophobic core between these helices and fβ1/2 (Fig. 2a; small red arrows). Third, the formation of the active conformation is accompanied by an inter-subunit reorientation of the complex, similar as described in a previously reported hyperactive HisFH variant containing an unnatural amino acid at the subunit interface^12^. This can, e.g., be visualized through the angle between the Cα atoms of fF120, hW123, and hG52 which is reduced from 25° in the inactive conformation to 10° in the active conformation (Fig. 2a). Concomitantly fα3/fα4/fβ4 of HisF are closing in on the HisH active site. Finally, in good agreement with the observed CSPs (Fig. 1d), the regions around hL1 and hL4 in HisH (Fig. 2b) are rearranged. As a consequence, the hydrogen bond between hP10 in hL4 and hV51 in hL1 is broken and the hG50/V51 peptide bond is flipped (Fig. 2d; red arrows). This exposes the amide groups of hV51 and hL85 and results in the formation of the proposed oxyanion hole. As a result, the amide group of the substrate Gln in the active site rotates into the newly formed oxyanion hole and forms hydrogen bonds with hV51/hL85 (through its amide carbonyl group) and hH178/hV140 (through its amide NH_2_ group). In this conformation the Gln is in a proper orientation for a nucleophilic attack by the catalytic cysteine hC84 (mutated to alanine in our structure). Due to the inter-subunit reorientation fD98 at the end of fα3 is brought closer to HisH (Fig. 2e; red arrows). Such a movement of fD98 has been postulated before based on increased HisH activity as observed for the fD98E mutant^6^. As a result, hN12 approaches the active site and donates hydrogen bonds to hE180, which is part of the catalytic triad, and to fD98. A new hydrogen bond from the hN12 backbone amide to the flipped hG50 carbonyl stabilizes the oxyanion hole. The inter-subunit orientation in the active conformation is stabilized by backbone hydrogen bonds that connect hL4 and hα1 in HisH with fα4 and fα3 in HisF. In summary the structure of the active conformation of HisFH shows large conformational changes in fL1/2 that form intimate contacts with ProFAR, a movement of the neighboring fα1/2 and a reorientation of the two subunits. Conformational changes in hL1/L2 and a reorientation of the substrate Gln in HisH lead to the formation of the oxyanion hole (Fig. 2a).

### Rapid Gln binding is followed by slower formation of the active conformation

**Fig. 3:**
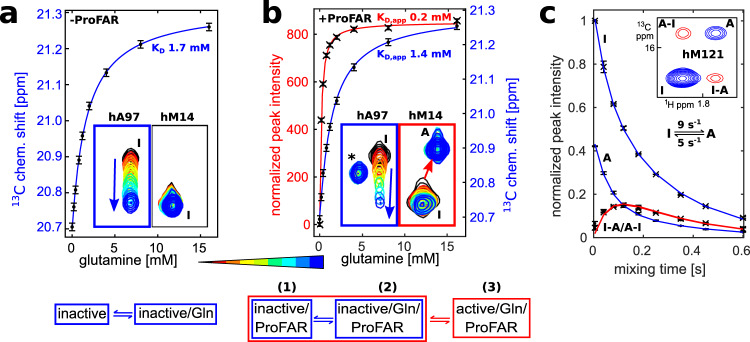
The HisFH inactive and active conformations are in a dynamic equilibrium. **a** Gln titration to HisFH–hC84S in the absence of ProFAR. Insets show closeups of methyl TROSY spectra for hA97 and hM14, which report on Gln binding (hA97) and formation of the active conformation (hM14). Gln binding to the inactive conformation (I) is fast on the NMR timescale and is followed based on the chemical shift of hA97 (blue arrow). The ^13^C chemical shift of hA97 is plotted against the Gln concentration (black dots) and a fit (blue line) to a two-state binding model is shown. The error-bars correspond to the spectral resolution. **b** Gln titration to HisFH–hC84S in the presence of ProFAR. The saturation of the HisF substrate binding site does not affect Gln binding to the inactive conformation (blue box). Concurrent with Gln binding to the inactive conformation the formation of the active conformation takes place. This conformational change is slow on the NMR timescale and is monitored based on the intensity of the hM14 signal (red box). With increasing Gln concentration the resonances of the inactive conformation decrease, while the signals of the active conformation (A) increase (black crosses). Note that the hA97 resonance intensity decreases with increasing Gln concentration due to the depletion of the inactive conformation. The signal of the active conformation of hA97 cannot be analyzed as it overlaps with other signals. The data are fitted to a three-state model (red and blue lines) that includes the formation of the active state. The error-bars correspond to the resolution (hA97) and noise level (hM14) of the spectra. The signal marked with an asterisk that appears next to hA97 belongs to the active conformation of hV100. The error-bars correspond to the spectral resolution. **c** Longitudinal ZZ-exchange experiments for HisFH–hC84S in the presence of ImGP/Gln reveal that the inactive and active conformations interconvert on the ms-time scale. The inset shows hM121 in the inactive (blue, labeled I) and active conformation (blue, labeled A) for a mixing time of 80 ms. The exchange peaks that originate from the interconversion between the two conformations are shown in red. Data are presented as mean values ± 1 standard deviation (*n* = 8 spectra). Source data are provided as a Source data file for all panels.

Based on our data above, it is evident that HisFH can undergo a transition from an inactive conformation to an active conformation upon interaction with its two ligands. To gain insights into this transition that go beyond the static pictures of the two crystallized conformations we returned to solution NMR experiments. To study the kinetics and thermodynamics of Gln binding to HisH and to assess how Gln binding influences the formation of the active conformation we performed Gln titrations in the presence and absence of ProFAR (Fig. 3a, b and Supplementary Fig. 17). In the absence of ProFAR, Gln binding takes place in fast exchange on the NMR timescale as evidenced by the gradual shift of the hA97 peak (Fig. 3a, blue arrow). The peak position of hA97 directly reports on the equilibrium between free and Gln-bound states and a fit of the binding curve reveals that HisFH–hC84S binds to Gln with a *K*_D_ of 1.7 ± 0.1 mM. Importantly, the hC84S mutation only minimally impacts Gln binding as WT HisFH binds Gln with a similar *K*_D_ of 1.3 ± 0.1 mM (Supplementary Fig. 17). The fast exchange behavior during the Gln titration implies that both Gln binding and dissociation are rapid. In agreement with this, a 2D-lineshape analysis (Supplementary Fig. 17) yields a *k*_off_ of 1600 ± 200 s^−1^ for WT HisFH and a *k*_off_ of 2100 ± 300 s^−1^ for HisFH–hC84S, which implies that the *k*_on_ is 1.2 × 10^6^ M^−1^ s^−1^ in both cases.

In the presence of ProFAR, Gln still binds to the inactive conformation in fast exchange as evidenced by the gradual shift of the hA97 peak (Fig. 3b, blue arrow), whose position reports on the equilibrium between the free and the Gln-bound inactive conformation. Moreover, we now observe the formation of the active HisFH conformation. This transition takes place in the slow exchange regime on the NMR timescale and is thus much slower than Gln binding (here we use the signal of hM14 as a reporter for this transition as it shows well resolved signals for both conformations). The inactive conformation signal (I) decreases in intensity during the Gln titration, whereas the active conformation signal (A) increases. We fitted the binding data to a three-state model considering the following states: (1) ProFAR bound inactive conformation, (2) ProFAR and Gln-bound inactive conformation, and (3) ProFAR and Gln-bound active conformation. We used the peak position of hA97 as a reporter for the equilibrium between states (1) and (2) and the intensity of the hM14 signal as a reporter for state (3). The fit reveals that the Gln affinity of the inactive conformation is not significantly changed by ProFAR (*K*_D,app_ 1.4 ± 0.1 mM compared to 1.7 ± 0.1 mM in the absence of ProFAR) and that the active conformation is favored relative to the inactive conformation when both substrates are bound. This is reflected in a higher apparent affinity for Gln for the active conformation (*K*_D,app_ 0.2 ± 0.1 mM). Based on these data, we conclude that Gln rapidly binds to the HisFH inactive conformation and that this binding then induces a partial transition of the complex into the active conformation when the HisF active site is occupied.

Above, we noted that the inactive conformation of the enzyme complex remains populated, even when both the HisF and the HisH active sites are saturated. To determine the interconversion rates between the inactive and active conformations under these conditions, we used longitudinal-exchange experiments (Fig. 3c). Sample stability during this long-term NMR experiment was ensured by using the product ImGP which is even more stable than ProFAR (Supplementary Fig. 18) and which is also able to induce the formation of the active conformation in the presence of Gln. Interconversion between the two conformations during a mixing delay of 80 ms leads to the appearance of exchange peaks (I–A and A–I; red) that connect the signals of the inactive and active conformation (I and A; blue). Fitting of the signal intensities for different mixing times (see Methods) reveals that inactive and active conformations exchange with rates of 5 s^−1^ (I to A) and 9 s^−1^ (A to I). This is 25 times faster than the turnover rate of WT HisFH in the presence of ImGP (0.2 s^−1^) indicating that the large structural changes that are associated with the transition from inactive to active conformation are not rate limiting for catalysis. Importantly, also in the presence of other HisF ligands the exchange rates are faster than the turnover rates, as can be judged from a longitudinal-exchange experiments with a single timepoint that could be recorded in the presence of ProFAR (Supplementary Fig. 18).

### HisF ligands and mutations modulate the equilibrium between inactive and active conformation

**Fig. 4:**
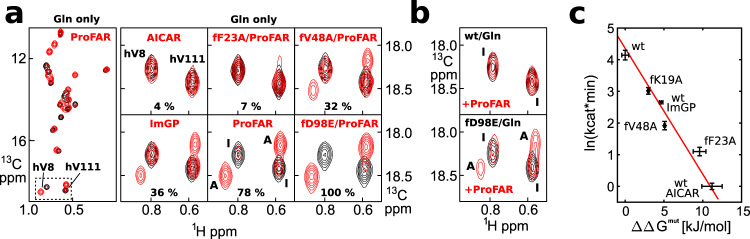
The activity of HisFH is correlated with the population of the active conformation. **a** Methyl TROSY spectra of NMR active HisH (hC84S background) that is incorporated in the HisFH complex either in the presence of saturating concentrations of Gln (black) or in the presence of saturating amounts of Gln and ProFAR (red). Weak signals of the inactive conformation are still present in the HisFH/Gln/ProFAR complex (left), indicating that the complex does not fully adopt the active conformation. The populations of inactive and active conformations are shifted by different HisF ligands and mutations (right, closeups of the hV8 and hV111 signals are shown). The populations of the active conformation are given at the bottom of the spectrum and the signals of the inactive (I) and active (A) conformations are labeled in the spectrum of the WT HisFH complex in the presence of ProFAR. **b** Methyl TROSY spectra of NMR active HisH in a fully active HisFH complex during catalytic turnover. Close-ups for hV8/hV111 in the presence of Gln/ProFAR during catalytic turnover (red) are shown for WT HisFH (top) and for the hyperactive HisFH–fD98E complex (bottom). The signals of the inactive (I) and active (A) conformations are labeled in the spectra. Under multiple turnover conditions, the active conformation is observed only for the fD98E mutant. Spectra of the inactive conformation (before addition of ProFAR) are shown in black. **c** Correlation between ln(*k*_cat_) of HisFH and ΔΔ*G*^mut^ of the active conformation for different mutants/ ligands. A linear fit with a slope of (−RT)^−1^ is shown (red line). This implies that the glutamine turnover rates in HisFH are directly determined by the population of the active conformation. Data are presented as mean values ± 1 standard deviation (based on 8 resonances (*x*-axis) or ≥ 28 timepoints(*y*-axis)). Source data are provided as a Source data file.

According to the framework of the ensemble allosteric model^1^, the position of the equilibrium between inactive and active conformation should dictate the glutaminase activity of the HisFH complex. In addition, the equilibrium should be shifted toward the inactive conformation for ligands that show weaker activation (e.g., ImGP or AICAR) or mutations that interfere with allosteric activation. To test whether the ensemble allosteric model applies to the allosteric activation mechanism of HisFH, we evaluated the modulation of the turnover rates and populations by different ligands and HisF mutations (Fig. 4a, Supplementary Table 2, Supplementary Fig. 19). To determine the populations of the inactive and active conformations we used HisFH complexes that contain the hC84S mutation as this is compatible with long-term NMR experiments. In the presence of saturating amounts of Gln and ProFAR the peak volumes of the inactive and active conformation indicate that 78 ± 3% of the complex is in the active conformation. This result is in good agreement with the value calculated from the apparent Gln-dissociation constants (Fig. 3b), which predict a population of 86 ± 7% active conformation under saturating concentrations of ProFAR and Gln. Under these conditions we determined that the WT HisFH complex shows a glutaminase activity (*k*_cat_) of 63 min^−1^.

In the next step, we analyzed various ligands and mutations (Fig. 4a, right subpanels). For the weaker glutaminase activators ImGP (*k*_cat_ = 14 min^−1^) and AICAR (*k*_cat_ = 1 min^−1^), the HisFH complex is shifted toward the inactive conformation (36 ± 2% and 4 ± 2% active state, respectively). In addition, we tested previously identified HisF mutations that interfere with the allosteric activation^9,30^ (fK19A, with *k*_cat_ = 20 min^−1^ and fV48A, with *k*_cat_ = 7 min^−1^) and found that these enzymes populate the active conformation to a lesser extent (52 ± 1% and 32 ± 1% active state, respectively) in the presence of saturating amounts of ProFAR and Gln. The structure of the active conformation shows that fF23 is important for the stabilization of the closed conformation of fL1 (Fig. 2c) and based on that we also prepared the fF23A mutant. As anticipated this mutation decreases the population of the active conformation (7 ± 1%) and concomitantly reduces the catalytic activity (*k*_cat_ = 3 min^−1^) compared to the WT protein. In contrast, the fD98E mutation that has been shown to increase the Gln turnover rates upon allosteric activation^6^ (*k*_cat_ = 179 min^−1^) results in a HisFH complex in which the inactive-active conformation equilibrium is shifted completely toward the active conformation (Fig. 4a). In summary, we observe a strong (Spearman’s rank correlation *r*_s_ = 1, *p* = 3.9 × 10^−4^), albeit nonlinear correlation between the population of the active conformation and the turnover rate of the complex (Supplementary Table 2, Supplementary Fig. 20).

As the ensemble allosteric model predicts a linear correlation between the active state population and catalytic activity, we wondered whether the nonlinearity of the correlation was caused by the inactivating hC84S mutant that we used for the NMR experiments. To be able to quantify the active conformation in the WT HisFH complex that is saturated with ProFAR we used high concentrations of Gln (100 mM), which allowed for the recording of a single methyl TROSY spectrum during catalytic turnover before the majority of the Gln was hydrolyzed (Fig. 4b, top panel, Supplementary Fig. 21). Interestingly, we only observed small CSPs characteristic for ProFAR and Gln binding (as observed in Fig. 1b, c), but no signals of the active conformation. This indicates that the steady-state population of the active conformation is below the detection limit of the NMR experiment (~5%). The population of the active conformation can, however, be shifted to the NMR observable range by replacing WT HisF with the activating fD98E mutant. For the HisFH–fD98E complex, we observe a population of the active conformation of 49 ± 8% (Fig. 4b, Supplementary Fig. 21) during multiple turnover. The fD98E induced increase in the population of the active conformation is in full agreement with the results obtained in the hC84S background, where the active conformation is also stabilized by the fD98E mutation (Fig. 4a).

It is, however, important to note that the low population of the active conformation in the HisFH complex during multiple turnover (<5%; WT enzyme) is in contrast to the populations that we determine in the hC84S background, where we observe 78 ± 3% active conformation. This discrepancy might be due to a thermodynamic stabilization of the active conformation due to the mutation in the active site or, alternatively, due to the slower Gln hydrolysis in the hC84S mutant, i.e., it might be a thermodynamic or a kinetic effect. To evaluate this, we performed titration experiments with ProFAR and the nonhydrolyzable Gln analog Albizziin, a reversible, competitive glutaminase inhibitor^29^ (Supplementary Fig. 22). This setup allows for a direct comparison between WT HisFH and HisFH–hC84S complexes. Here, we observed no active conformation for ProFAR/WT HisFH in the presence of Albizziin, whereas for the HisFH–hC84S mutant the active conformation is clearly visible. Based on that, we conclude that the hC84S mutation thermodynamically stabilizes the active conformation relative to the WT complex. This finding shows that a direct comparison between the population of the active conformation as determined in the hC84S mutant background and the turnover rates of the WT HisFH complex is not possible.

### A quantitative correlation between the population of the active conformation and the activity of the complex

To enable a quantitative comparison between the turnover rates measured for the WT complex and the populations determined in the hC84S mutant background we here make use of an alternative approach. Instead of comparing the absolute population of the active state in the hC84S background, we determine the destabilization of the active conformation (ΔΔ*G*^mut^) caused by HisF mutations or HisF ligands with weaker glutaminase activation than ProFAR. This destabilization can be readily measured in the hC84S mutant background, for which the populations of the active conformation for various HisFH mutants are directly observable by NMR (Fig. 4a). The prerequisite for this approach is that HisF mutations and weaker activating ligands lead to a similar destabilization of the active conformation in the WT complex and in the hC84S mutant. This assumption is reasonable as the HisF active site and the HisF mutations are remote from the hC84S mutation. In agreement with that, we observe that the HisF NMR spectra are not perturbed by the HisH hC84S mutation. In case the population p_A_ of the active conformation is low (as is the case for the WT complex during catalysis) the change in the population of the active conformation is given by exp(−ΔΔ*G*^mut^/RT) (see Methods). Experimentally, we indeed observe a linear relationship between ΔΔ*G*^mut^ (measured in the hC84S background) and ln(*k*_cat_) (measured in the WT HisH complex) with the expected slope of (*−*RT*)*^*−1*^ (Fig. 4c). This important finding implies that the turnover rate *k*_cat_ is directly determined by the population of the active conformation. Our data thus reveal a thermodynamic model for the allosteric mechanism during catalysis, where the activating ligands (AICAR, ImGP, and ProFAR) stabilize the active conformation to a different degree. This finding is in excellent agreement with the ensemble allosteric model, which predicts a direct correlation between the population of the active conformation and the glutaminase activity.

## Discussion

The production of ammonia through deamination of glutamine is a central step in the biosynthesis of a large variety of bio-molecules. Because of the high reactivity of ammonia a tight coupling between glutaminase activity and the subsequent synthase reaction is required to prevent the unproductive turnover of glutamine^4^. One of the most tightly coupled enzyme systems that is known to date is the bi-enzyme HisFH complex. In this enzyme complex, the HisF glutaminase activity is increased by more than three orders of magnitude upon recruitment of the ammonia acceptor PrFAR to HisH^9,13^. A large number of studies have addressed the mechanism behind the allosteric pathway that links the two active sites that are over 25 Å remote. These efforts include computational work that suggest changes in motions on the ns timescale, including small (e.g., PrFAR dependent) inter-subunit motions^22,23,25,26^ and NMR studies that have revealed increased dynamics in HisF upon binding of activating ligands^27,30^. Our findings go significantly beyond those previous results.

**Fig. 5:**
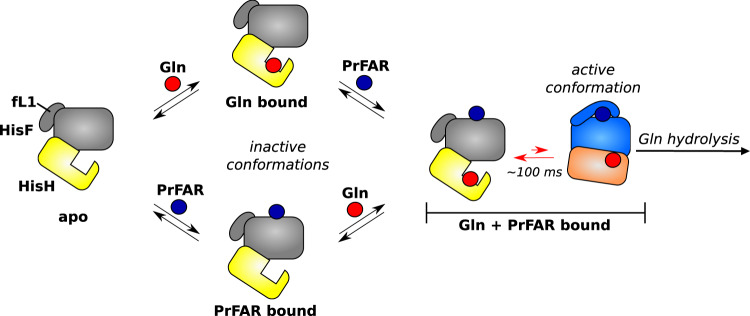
Model of the allosteric activation pathway of the HisFH complex. The HisFH complex adopts the inactive conformation (gray and yellow) in the apo state (left) and in the presence of one of the two substrates (Gln: red; PrFAR: blue). In the inactive conformation fL1 of HisF is in the open orientation, the HisF–HisH interface is open and the oxyanion hole is not formed. Binding of the second ligand (either PrFAR or Gln) results in the formation of the HisFH:PrFAR:Gln complex. The presence of both ligands enables the formation of the active conformation of the complex (marine and orange). This active conformation is, however, only sparsely populated and in equilibrium (indicated by red arrows) with the inactive conformation (timescale ~100 ms). In the active conformation fL1 is closed over the PrFAR substrate in the HisF active site, the HisF–HisH domains are in a closed orientation and the oxyanion hole in the HisH active site is formed, which enables Gln hydrolysis. The population of the active conformation ultimately determines the overall catalytic rate of the HisFH complex.

The NMR experiments (Figs. 1, 3), crystal structures (Fig. 2) and enzyme activity data (Figs. 3, 4) that we present here reveal the molecular details of the intricate and long-range allosteric mechanism that regulates the glutaminase activity of the HisFH enzyme complex (Fig. 5). We find that the presence of both substrates is a prerequisite for the activation of the allosteric pathway that links the HisF and HisH active sites (Fig. 1b–d). This results in the formation of the active enzyme conformation, which is accompanied by significant structural changes in four separate regions in the HisFH enzyme: First, the side in HisF that ranges from fL1 to the end of fα3 is remodeled upon HisF substrate binding (Fig. 2b). Then, the hydrophobic core in HisF is rearranged. This then, thirdly, changes the HisF–HisH inter-subunit orientation as well as the HisH region surrounding hL1 (Fig. 2e). Finally, these events prime the HisH active site for the formation of the oxyanion hole (Fig. 2d) that can be stably formed only in the presence of Gln (Fig. 1). Most of these central steps have not been observed to this degree in previous studies. We anticipate that this is due to the slow (ms timescale) exchange between the inactive and active conformations in solution which is hard to detect or predict computationally. In addition, previous NMR experiments have failed to reveal the active conformation of the complex due to the use of PrFAR (that is highly unstable; Supplementary Fig. 4) and the HisH inhibitor acivicin (that is not able to induce the formation of the active conformation Supplementary Fig. 23).

A central aspect of the extraordinarily tight regulation of the glutaminase activity is the absence of a pre-formed oxyanion hole in the isolated HisH enzyme^33^ and in the apo HisFH complex. As a result, Gln is not turned over at biologically relevant rates by HisH alone or by the HisFH complex in the absence of a HisF substrate. Interestingly, in other, less tightly regulated, synthase complexes that contain a class I glutaminase, the oxyanion hole appears to be fully present in the absence of the ammonia acceptor (e.g., Pdx2 in the pyridoxal 5′-phosphate synthase complex)^17^ (Supplementary Fig. 24). In that light, the long-range allosteric activation in HisFH, which ultimately results in the formation of the HisH oxyanion hole when both substrates are present, is a unique mechanism that links the glutaminase and cyclase reactions. The hydrolysis of glutamine to glutamate in the HisH active site (Supplementary Fig. 1) involves two chemical steps: the formation of a thioester intermediate and the hydrolysis of this intermediate. Both steps will be significantly enhanced by the formation of the oxyanion hole in the active conformation of HisFH.

Our data is compatible with a situation where thioester hydrolysis is faster than thioester formation, as we observe that the enzyme is mainly in the ground conformation during multiple turnover conditions (Fig. 4b). In case the thio-ester hydrolysis would be slow, the intermediate would accumulate, and we would observe the complex in the active conformation (as can be judged from the NMR in the presence of the DON thioester mimic; Supplementary Fig. 12).

Our work also highlights that bio-molecules and especially enzymes are highly dynamic and sample different structural states. These dynamic aspects are often important for function but easily escape our attention based on static crystal structures^34–37^. Here, we show that the HisFH complex that is saturated with both substrates exchanges between the inactive and the catalytically competent active conformation (Figs. 3–5). The population of the active conformation depends on the ligand that is bound to HisF and can also be increased or decreased by specific point mutations (Fig. 4a, b). Importantly, we establish that there is a strong and direct correlation between the thermodynamic stability of the active conformation and the turnover rate of the complex (Fig. 4c). As the exchange rates between the inactive and active conformations are fast compared to the catalytic turnover rates (Fig. 3c), this provides compelling evidence that the ensemble allosteric model underlies HisFH activity. Consequently, the glutaminase activity is directly determined by the population of the active conformation. In that light, the regulation of the HisFH enzyme appears to have evolved in a manner that ensures a fine-tuning of the population of the active conformation (Fig. 5). This mechanism might be generally employed by GATs to regulate their catalytic activity. Interestingly, also for other enzymes, including aspartate transcarbamoylase^38^, effector binding has been shown to modulate a conformational equilibrium between two states with different substrate affinity. Together with our findings this fits into a more general picture of allosteric activation of large molecular assemblies, including the proteasome^39^ and GPCRs^40–42^ where shifts in conformational equilibria have been described as the basis for the allosteric mechanism^43^.

In summary, we here show that a comprehensive description of allosteric processes necessitates a detailed understanding of the structural changes, populations, and the associated interconversion rates (Fig. 5). This notion will spark further investigations that address how complex biological processes are regulated to ensure cellular homeostasis. In addition, our results will support current and very challenging efforts to design enzymes that are allosterically regulated^44^.

## Methods

### Protein expression and purification

All experiments were performed with *Thermotoga maritima* HisFH complexes. HisH (Uniprot ID: Q9X0C8) and HisF (Uniprot ID: Q9X0C6), as well as mutant forms of the proteins (Supplementary Table 3), were expressed from modified pET vectors and purified as described below. Unlabeled (NMR inactive) proteins were expressed in LB medium. Isotopically labeled HisFH (histidine ring-C2-^13^C or ILMVA methyl group labeling) and HisF (^15^N labeling) (both with an N-terminal, TEV-cleavable His-tag) were expressed in D_2_O-based M9 minimal medium. Expression was performed at 20–25 °C overnight in *E. coli* BL21 Gold (DE3) (NMR inactive; Agilent Technologies) or in *E. coli* BL21-CodonPlus (DE3)-RIL cells (Stratagene) after induction with 0.5–1 mM Isopropyl β-D-1-thiogalactopyranoside (IPTG) at an OD_600_ of 0.6–0.8. For ^15^N labeling of HisF 0.5 g/l ^15^NH_4_Cl was used. Specific ^1^H^13^C labeling of the methyl groups of alanine (A), isoleucine (I), leucine/valine (L/V) and methionine (M) in HisFH was achieved by addition of 100 mg L-Alanine-3-^13^C,2-^2^H, 60 mg 2-ketobutyric acid-4-^13^C,3,3-^2^H_2_, 100 mg 2-keto-(3-methyl-^13^C)-butyric acid-4-^13^C,3-^2^H and 100 mg L-methionine-(methyl-^13^C) per liter of D_2_O-based M9 medium. In order to label only valine methyl groups 2-keto-(3-methyl-^13^C)-butyric acid-4-^13^C,3-^2^H was used in combination with 150 mg/l unlabeled 4-methyl-2-oxovalerate^45^. For histidine labeling 100 mg/l ring-^13^C2 labeled histidine (Cambridge Isotope Laboratories) was used. All labeled precursors/amino acids were added 1 h prior to induction except for alanine, which was added 15–20 min before induction.

For NMR inactive proteins, cells were harvested by centrifugation, resuspended in 50 mM (tris(hydroxymethyl)aminomethane) Tris-HCl pH 7.5 (HisF without purification Tag), 50 mM Tris-HCl pH 7.5, 100 mM NaCl, 10 mM imidazole (HisF), or 50 mM potassium phosphate pH 7.8, 100 mM NaCl, 10 mM imidazole (HisH), and lysed by sonication. *E. coli* proteins were precipitated by a heat shock (15 min, 70 °C). Cell debris and precipitated proteins were removed by centrifugation and the supernatant was either subjected to ion-exchange chromatography with a MonoQ column (HR 16/10, 20 ml, Pharmacia; HisF without purification Tag) or nickel-affinity chromatography (HisTrap FF Crude column, 5 ml, GE Healthcare). Proteins were eluted with a linear gradient of NaCl (0–3 M) from the MonoQ column, and with a linear gradient of imidazole (10–750 mM) from the nickel column. Fractions containing the proteins of interest were identified by sodium dodecyl sulfate-polyacrylamide gel electrophoresis (SDS-PAGE), and pooled. Eluted HisF proteins without purification Tag were dialysed against the previously used buffer without NaCl. To remove DNA contaminations, ammonia sulfate precipitation was applied to the proteins. Finally, the proteins were further purified with a size-exclusion chromatography column (Superdex 75 HiLoad 26/600, GE Healthcare) by using 50 mM Tris-HCl pH 7.5 (HisF) or 50 mM potassium phosphate pH 7.8 and 100 mM NaCl (HisH) as the running buffer. Eluted proteins with a N-His6-Tag were digested with TEV protease at room temperature over night during dialysis against 50 mM Tris-HCl pH 7.5 (HisF) or 50 mM potassium phosphate pH 7.8 and 100 mM NaCl (HisH). The remaining TEV protease was then removed by nickel-affinity chromatography (HisTrap FF Crude column, 5 ml, GE Healthcare) with a linear gradient of imidazole (0–750 mM). Fractions at a low imidazole concentration containing the proteins of interest were identified by SDS-PAGE analysis, pooled, and further purified with a size-exclusion chromatography column as described above. Eluted protein fractions were checked by SDS-PAGE for >90% purity, pooled, concentrated, and dripped into liquid nitrogen for storage at −80 °C.

The HisFH-hC84A complex (HisF-wt without tag, HisH-C84S with a TEV-cleavable N-His_6_-Tag) for crystallization was purified by combining the cell lysates after separate expression before the NiNTA column and purified analogously to the NiNTA purification described above, but with the difference that the heat shock was omitted, and that the final size-exclusion chromatography was performed using the following buffer: 10 mM Tris-HCl pH 8.0, 100 mM NaCl.

For isotopically labeled proteins, cells were harvested by centrifugation, resuspended in buffer A (400 mM NaCl, 50 mM sodium phosphate pH 7.4, 10 mM imidazole) supplemented with lysozyme and DNase I and lysed by sonication. *E. coli* proteins were precipitated by a heat shock (20 min, 70 °C). Cell debris and precipitated proteins were removed by centrifugation and the supernatant was applied to gravity flow NiNTA columns equilibrated in buffer A. Columns were washed with buffer A containing 20 mM imidazole and proteins were eluted with buffer B (150 mM NaCl, 25 mM sodium phosphate pH 7.4) supplemented with 300 mM imidazole. Eluted proteins were digested with TEV protease at room temperature over night during dialysis against buffer B supplemented with 1 mM Dithiothreitol (DTT) (molecular weight cut off 3.5 kDa). After dialysis HisF and HisH were combined at a 1.3/1 ratio and free HisF was removed by size-exclusion chromatography using a 16/60 Superdex 75 column equilibrated in NMR buffer (50 mM NaCl, 20 mM 4-(2-hydroxyethyl)−1-piperazineethanesulfonic acid (HEPES) pH 7.3, 1 mM DTT).

### Crystallization

For crystallization of the HisFH-hC84A complex in the presence of Gln and ProFAR we used 40% (w/v) pentaerythritol (5/4 PO/OH), 0.2 M sodium thiocyanate, 0.1 M HEPES (pH 7.5), and 10 mM L-glutamine. Crystals grew after mixing 2 µl at 30 mg/ml protein solution in size-exclusion buffer (10 mM Tris-HCl pH 8.0, 100 mM NaCl) with 2 µl of the reservoir solution at 18 °C by the hanging‐drop vapor diffusion method. 10 mM ProFAR dissolved in size-exclusion buffer was added by soaking. Crystals appeared after 2 days and reached the final size (0.3 × 0.4 × 0.1 mm) after three to four additional days. Of note: crystals of the HisFH–hC84S complex dissolved during soaking and could thus not be used for crystallographic studies.

### Data collection, structure determination, and refinement

Diffraction data were collected at a wavelength of 0.97625 Å and temperature of −170 °C using flash‐frozen crystals at the EMBL/DESY beamline P13. The collected data was first integrated using XDS^46^ and merged and scaled using the CCP4 suite program AIMLESS^47,48^. The structures of the HisFH-hC84A complex were determined by the molecular replacement method with the program PHASER^49^ using the previously published apo-HisFH structure^11^ (PDB: 1gpw) as search model. An electron density map at 2.06 Å resolution was generated with the PHENIX program^50^. The crystals contained two HisFH-ProFAR-Gln complexes and one HisFH–Gln complex per asymmetric unit. Iterative rounds of manual model building using COOT^51^ and PHENIX refinement^52^ on xyz coordinates, optimize X-ray/ADP weight, NCS, and TLS were carried out to build the complete model. The final refinement statistics are summarized in Supplementary Table 1. Coordinates and structure factors have been deposited in the PDB with accession code 7AC8.

### NMR measurements

NMR experiments were performed at 30 °C (except for NOESY spectra, which were recorded at 50 °C) on Bruker 500, 600, and 800 MHz spectrometers equipped with NEO AVANCE consoles and cryogenically cooled probes (nitrogen cooled (500 and 600 MHz) or helium cooled (800 MHz)). All measurements were done in NMR buffer supplemented with 5% D_2_O for the lock. Protein concentrations were 50–150 µM for titration experiments and 300–500 µM for NOESY- and ZZ-exchange experiments. Methyl ^1^H^13^C-HMQC and histidine ring^−13^C2 ^1^H^13^C-HMQC spectra were recorded using the SOFAST-HMQC pulse sequence^53,54^, HCH- and CCH-NOESY were recorded using the pulse sequences from Rossi et al.^55^. The pulse sequences for the ^1^H^13^C and ^1^H^15^N longitudinal exchange experiments were generously provided by Lewis E. Kay (University of Toronto). All other spectra were recorded using standard Bruker pulse sequences. Spectra were processed using Bruker Topspin 4.0 and NMRPipe^56^ and analyzed using CARA^57^, Sparky^58^, and NMRDraw^56^.

The backbone amide resonance assignments of HisF in the free state^59^ and the partial assignment of HisF bound to HisH^60^ were taken from the literature and were transferred to our buffer conditions using an HNH-NOESY spectrum.

The assignment of the ILMVA methyl groups of HisH in the free HisFH complex (inactive state) was obtained by comparison of the peak pattern in HCH- and CCH-NOESY spectra with the high-resolution crystal structure of the HisFH complex (pdb: 1gpw). Initial assignments were obtained using the MAGIC assignment algorithm^61^ in combination with the CCH-NOESY spectrum and the amino acid type of the methyl group signals. The latter was obtained by comparison of spectra of ILMVA-labeled samples to spectra of samples where only IA- or V-methyl groups were labeled^45^. These initial MAGIC assignments served as a starting point for a manual assignment. The final assignment was verified using a mutational approach. To this end the following HisH mutants were prepared: I106L, V111A, V51A, L153V, V81I. Finally, the assignments were transferred to 30 °C by tracking the peak changes in a temperature series from 50 °C to 30 °C.

For the methyl group assignment of the active conformation of HisH in the HisFH complex a sample of ILMVA labeled HisH-wt complexed with unlabeled HisF-D98E was reacted with DON in the presence of 10 mM ImGP and 5 mM AICAR. This leads to >95% active conformation and the long-term stability of the sample allowed for recording of HCH- and CCH-NOESY spectra of the active conformation. The assignments of the inactive state were then transferred to the active conformation based on the HCH-/CCH-NOESY spectra and on the ^1^H^13^C ZZ exchange spectra of HisFH–hC84S/ImGP/Gln sample that shows crosspeaks between the inactive and active conformations.

Assignments of H2/C2 signals of the histidines in HisH was achieved by a mutational approach. To this end the two double mutants hC84S/hH178A and hC84S/hH53A were used. Use of the hH53A mutant was necessary as the hH178A mutation prevents the formation of the active conformation and thereby the assignment of the hH178 signal by mutation. All signals except for hH178 and hH53 show only minor CSP between the Gln-bound and the active conformation, but for hH178 and hH53 the CSP are too large to unambiguously assign the signals in the active conformation. As the hH53 does not impede active state formation, its signals can be readily assigned in the hH84S/hH53A double mutant in the active conformation indicating that the other signal belongs to hH178.

The populations of the inactive and active conformation were determined based on the peak volumes of the following signals in both states: hM14, hV8, hL45, hM58, hV80, hL89, hL189, hL190. The reported values are the mean and the standard deviation of the populations for these residues. SOFAST-HMQC spectra for WT HisFH were recorded in the presence of 20 mM Gln and either 10 mM ImGP, 10 mM AICAR or 0.4 mM ProFAR. To determine the influence of the HisF mutations (fK19A, fF23A and fD98E) the spectra were recorded in the presence of 20 mM Gln and 0.4 mM ProFAR. For measurements under multiple turnover conditions SOFAST-HMQC experiments were recorded on a sample containing 50 µM HisH-wt/HisF-wt (or HisF-fD98E), 400 µM ProFAR and 100 mM Gln.

CSPs were calculated according to CSP = (((ΔC/4)^2 + (ΔH)^2))^(0.5) (with ΔC, ΔH chemical shift difference in ppm in the ^13^C and ^1^H dimension).

### Glutaminase assays

Activity measurements were performed on a 500 MHz NMR spectrometer in NMR buffer at 30 °C using 0.75–20 µM HisFH complex (depending on the activity). Following concentrations of substrate and HisF ligands were used: Gln 10 mM, ImGP 10 mM, AICAR 10 mM, ProFAR 0.3 mM. Gln hydrolysis was monitored by recording 1D ^1^H spectra every 34 s. The fractions of Gln and Glu were determined by integrating the signals of the Hγ signals at 2.42 ppm (Gln) and 2.32 ppm (Glu) and normalizing them by the combined integrals using MATLAB. Turnover rates were determined by linear fitting of the reaction curves (Supplementary Fig. 25). Reported values are the mean and the standard deviation of at least two independent experiments.

### Gln titration analysis

Titration curves for Gln binding in the absence and presence of ProFAR were analyzed with Dynafit^62^. In the absence of ProFAR a two-state model was used to fit the peak position of hA97 versus the Gln concentration. In the presence of ProFAR a three-state model was used (1. inactive state/ProFAR, 2. inactive state/ProFAR/Gln, 3. active conformation/ProFAR/Gln), where the equilibrium between states 1 and 2 was determined from the peak position of hA97 and the population of the active conformation was set proportional to the peak intensity of the hM14 signal of the active conformation.

The line-shape fitting of the Gln titrations to determine the on- and off-rates was performed with TITAN^63^. Errors were calculated using the jackknife approach implemented in TITAN.

### Correlating ln(*k*_cat_) and ΔΔ*G*^mut^

Under the assumption that thioester formation is rate limiting, the turnover rate of the complex in the presence of saturation concentrations of ProFAR and Gln is determined by the population of the active conformation *P*_a_ and the rate of thioester formation of the active conformation *T*_act_. (The rate of thioester formation of the inactive conformation is assumed to be negligible):

1 $${k}_{{\rm{cat}}}={P}_{{\rm{a}}}\ast {T}_{{\rm{act}}}$$

The population of the active conformation is well approximated by the equilibrium constant *K*_a_ between active (*P*_a_) and inactive conformation (*P*_b_), when the population of the active conformation is <<1:

2 $${K}_{{\rm{a}}}={P}_{{\rm{a}}}/{P}_{{\rm{b}}}={P}_{{\rm{a}}}/(1-{P}_{{\rm{a}}})\,{\rm{for}}\,{P}_{{\rm{a}}}\ll 1:{K}_{{\rm{a}}}={P}_{{\rm{a}}}$$

The equilibrium constant *K*_a_ between active and inactive conformation is determined by the free energy difference Δ*G* between the two conformations:

3 $${K}_{{\rm{a}}}=\exp (-\Delta {{G}}/{\rm{RT}})$$

Combining (1), (2), (3) we can relate the *k*_cat_ of the complex with the free energy difference between the two conformations in general:

4 $${k}_{{\rm{cat}}}={T}_{{\rm{act}}}\ast \exp (-\Delta {{G}}/{\rm{RT}})$$

and for the WT complex (with Δ*G*_WT_ = free energy difference between the two conformations for the WT complex):

5 $${k}_{{\rm{cat}}/{\rm{WT}}}={T}_{{\rm{act}}}\ast \exp (-\Delta {{{G}}}_{{\rm{WT}}}/{\rm{RT}})$$

In case of a HisF mutation or another activating ligand (both will be subsumed under mutation in what follows) the free energy difference between the two conformations is changed by ΔΔ*G*^mut^:

6 $$\Delta {{{G}}}_{{\rm{mut}}}=\Delta {{{G}}}_{{\rm{WT}}}+\Delta \Delta {{{G}}}^{{\rm{mut}}}$$

The turnover rate of the mutant complex *k*_cat/mut_ is thus given by combining (4) and (6):

7 $${k}_{{\rm{cat}}/{\rm{mut}}}={T}_{{\rm{act}}}\ast \exp (-(\Delta {{{G}}}_{{\rm{WT}}}+\Delta \Delta {{{G}}}^{{\rm{mut}}})/{\rm{RT}})$$

By combining (5) and (7) the turnover rate of the mutant complex can be expressed relative to the WT complex:

8 $${k}_{{\rm{cat}},{\rm{mut}}}={k}_{{\rm{cat}},{\rm{wt}}}\ast \exp (-\Delta \Delta {{{G}}}^{{\rm{mut}}}/{\rm{RT}})$$

A linear relation between ΔΔ*G*^mut^ and ln(*k*_cat,mut_) with a slope of −(1/RT) is thus expected:

9 $${\mathrm{ln}}({k}_{{\rm{cat}},{\rm{mut}}})=\,{\mathrm{ln}}({k}_{{\rm{cat}},{\rm{wt}}})-(1/{\rm{RT}})\ast \Delta \Delta {{{G}}}^{{\rm{mut}}}$$

### ProFAR/PrFAR synthesis

The HisF ligands were synthesized enzymatically from 5-phospho-D-ribosyl α-1-pyrophosphate and adenosine triphosphate using the purified enzymes HisE/IG^64^. The progress of the reaction was traced spectrophotometrically and the ProFAR product was purified using ion-exchange chromatograph (POROS column; HQ 20, 10 mL, Applied Biosystems, using a linear gradient of 50 mM to 1 M NH_4_COOCH_3_). ProFAR purity was examined through the absorbance ratio A_290_/A_260_ and the concentration was determined at a wavelength of 300 nm (ε_300_ = 6069 M^−1^ cm^−1^). PrFAR was obtained by reacting ProFAR with HisA. The product was purified using ion-exchange chromatograph as described for ProFAR.

The enzymes (HisA and HisE/IG) were purified by standard methods from *E. coli* BL21 Gold cells (DE3) (Agilent Technologies) that overexpressed the respective proteins.

### Reporting summary

Further information on research design is available in the Nature Research Reporting Summary linked to this article.

## Supplementary information

Supplementary Information

Peer Review File

Reporting Summary

## Data Availability

The atomic coordinates and structure factors for the HisF–HisH complex have been deposited in the Protein Data Bank under accession codes 7AC8. All relevant data are available from the authors. Source data are provided with this paper.

## Source data

Source Data
