# Supplementary material for: Molecular basis for the allosteric activation mechanism of the heterodimeric imidazole glycerol phosphate synthase complex
Source: Nat Commun. 2021 May 12;12:2748. doi: 10.1038/s41467-021-22968-6 (PMC8115485; doi:10.1038/s41467-021-22968-6)
Supplement: Supplementary file 1 — Supplementary Information [file 41467_2021_22968_MOESM1_ESM.pdf]

Supplementary information for:

Molecular basis for the allosteric activation mechanism of the heterodimeric imidazole glycerol phosphate synthase complex.

Jan Philip Wurm[1†], Sihyun Sung[2†], Andrea Christa Kneuttinger[1], Enrico Hupfeld[1], Reinhard Sterner[1], Matthias Wilmanns[2,3,], Remco Sprangers[1,*]*

[1] Institute of Biophysics and Physical Biochemistry, Regensburg Center for Biochemistry, University of Regensburg, 93053 Regensburg, Germany.
[2] European Molecular Biology Laboratory, Hamburg Unit, Notkestraße 85, 22607 Hamburg, Germany
[3] University Hamburg Clinical Center Hamburg-Eppendorf, Martinistraße 52, 20246 Hamburg
[†] these authors contributed equally

This file contains:

Supplementary Figures S1-S25 and Supplementary Tables 1-3

Table of contents

S2 ***Supplementary Fig. 1 | Proposed catalytic mechanism of the glutaminase reaction in HisH***
S3 ***Supplementary Fig. 2 | Methyl group assignments of HisH in the HisFH complex***
S4 ***Supplementary Fig. 3 | Assignment of the catalytic histidine hH178 in HisH***
S6 ***Supplementary Fig. 4 | Stability of PrFAR and ProFAR in NMR buffer at 30 °C***
S8 ***Supplementary Fig. 5 | The HisFH-hC84S mutant complex shows very low glutaminase activity***
S9 ***Supplementary Fig. 6 | Full NMR spectra of Gln and ProFAR titrations to WT HisFH***
S10 ***Supplementary Fig. 7 | Full NMR spectra of Gln and ProFAR titrations to HisFH-hC84S***
S11 ***Supplementary Fig. 8 | Comparison of WT HisFH and the HisFH-C84S mutant spectra***
S12 ***Supplementary Fig. 9 | CSP in HisF upon Gln binding to HisFH***
S13 ***Supplementary Fig. 10 | CSPs in HisH elicited by binding of Gln, ProFAR and Gln+ProFAR***
S14 ***Supplementary Fig. 11 | The active conformations of HisFH-hC84S and HisFH-hC84A are virtually identical***
S15 ***Supplementary Fig. 12 | Active conformation for the WT HisFH complex in the presence of DON and ProFAR***
S17 ***Supplementary Fig. 13 | Equilibrium between inactive and active conformation for the HisFH-hC84S complex***
S18 ***Supplementary Fig. 14 | Crystal packing***
S19 ***Supplementary Fig. 15 | Electron density maps***
S21 ***Supplementary Fig. 16 | Comparison between HisFH structures***
S23 ***Supplementary Fig. 17 | Gln titrations to the HisFH complex***
S24 ***Supplementary Fig. 18 | ImGP is stable during longitudinal ZZ-exchange experiments***
S25 ***Supplementary Fig. 19 | The population of the active conformation of HisFH can be modulated***
S27 ***Supplementary Fig. 20 | Correlation between the k_{cat} and the population of the active conformation***
S28 ***Supplementary Fig. 21 | NMR spectra under multiple turnover conditions***
S29 ***Supplementary Fig. 22 | Albizziin can induce the formation of the active conformation***
S31 ***Supplementary Fig. 23 | The HisFH/acivicin/ProFAR complex does not form the active conformational***
S33 ***Supplementary Fig. 24 | The oxyanion holes of Pdx2 and of HisH in the active conformation***
S34 ***Supplementary Fig. 25 | 1D-^{1}H NMR based glutaminase assay for WT HisFH in the presence of ProFAR***
S35 ***Supplementary Table 1 | Crystallographic data and refinement***
S36 ***Supplementary Table 2 | Influence of activators and HisFH mutants***
S37 ***Supplementary Table 3 | Plasmids used for protein expression***

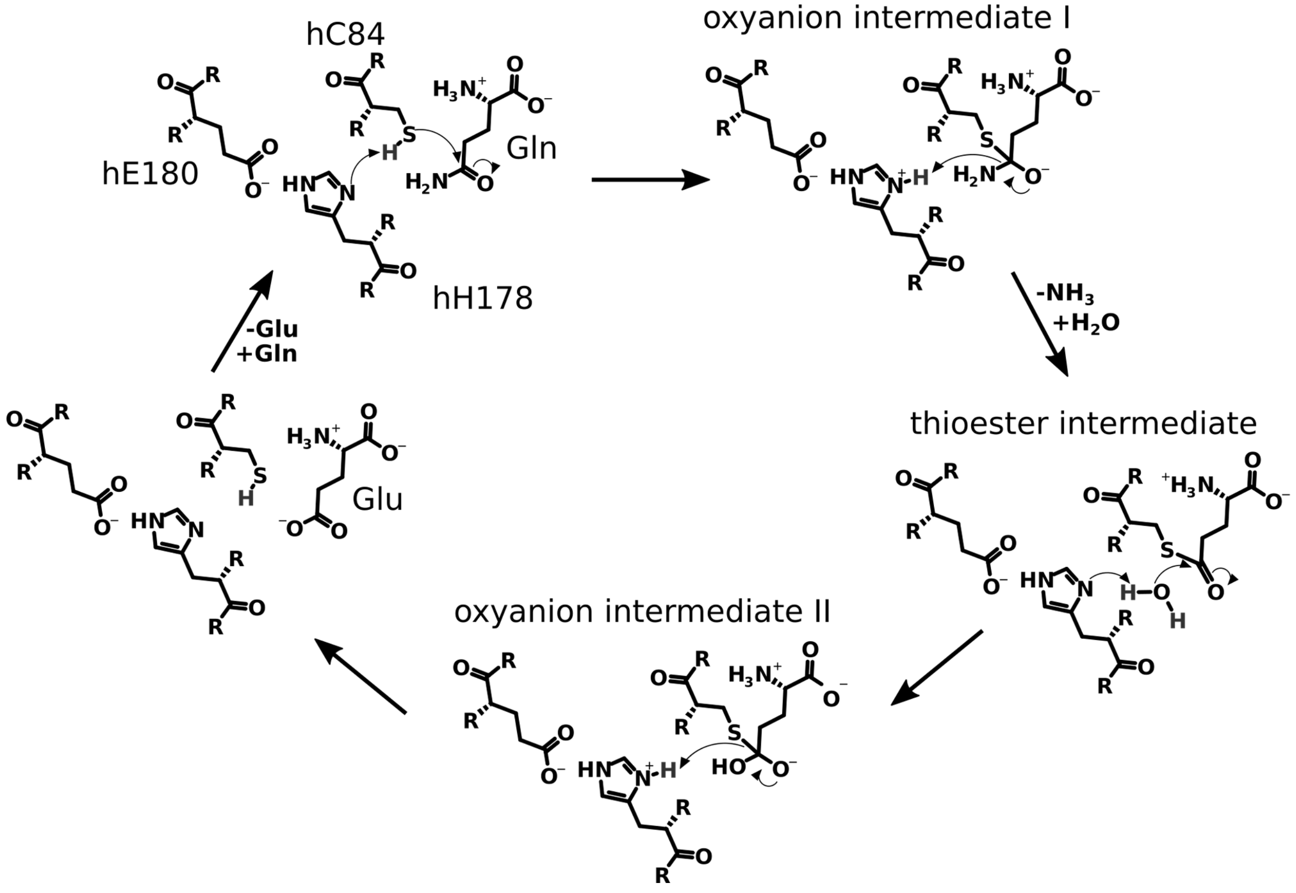

Supplementary Fig. 1| Proposed catalytic mechanism of the glutaminase reaction in HisH *(adopted from [1]).*

The HisH reaction starts with the nucleophilic attack of hC84 on the Gln amide group (top left), which leads to the formation of the tetrahedral oxyanion intermediate I (top right). Proton transfer from hC84 to hH178 might take place before or concomitant with the nucleophilic attack. Collapse of the tetrahedral intermediate I liberates the ammonia and generates the thioester intermediate (right). Finally, the thioester is hydrolyzed via the oxyanion intermediate II (bottom) yielding glutamate (left).

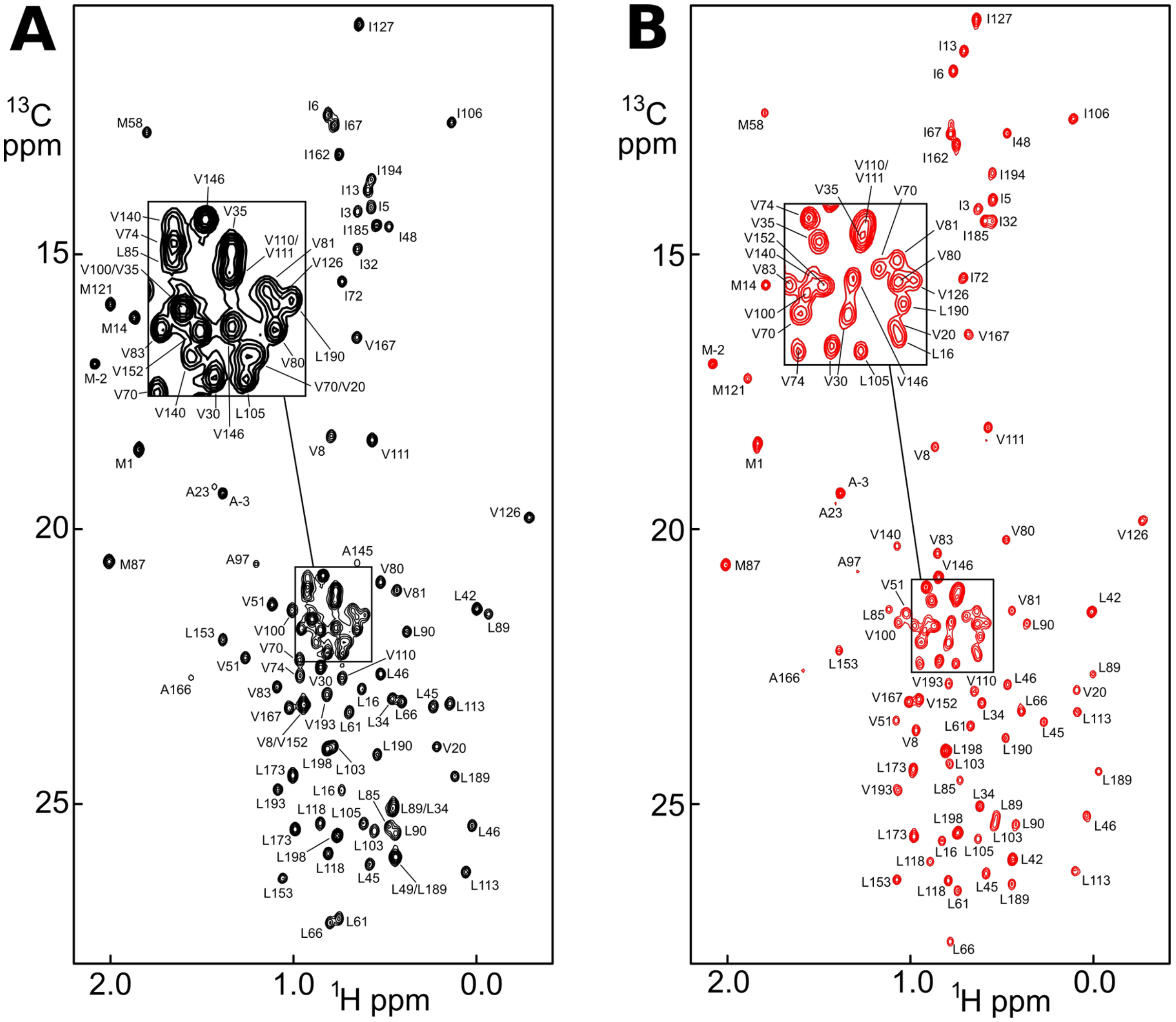

Supplementary Fig. 2 | Methyl group assignments of HisH in the HisFH complex in the apo state and in the active conformation.

*(**A**) Assignment of the apo state. Methyl-TROSY spectrum of WT HisFH (HisF $^{2}H^{15}N$-labeled, HisH ^{2}H, ILMVA methyl-$^{1}H^{13}C$-labeled)*

*(**B**) Assignment of the active conformation. Methyl-TROSY spectrum of HisFH-hC84S (HisF $^{2}H^{15}N$-labeled, HisH-hC84S ^{2}H, ILMVA methyl-$^{1}H^{13}C$-labeled) in the presence of Gln and ProFAR. The contour levels are chosen such that only the signals of the active conformation are visible.*

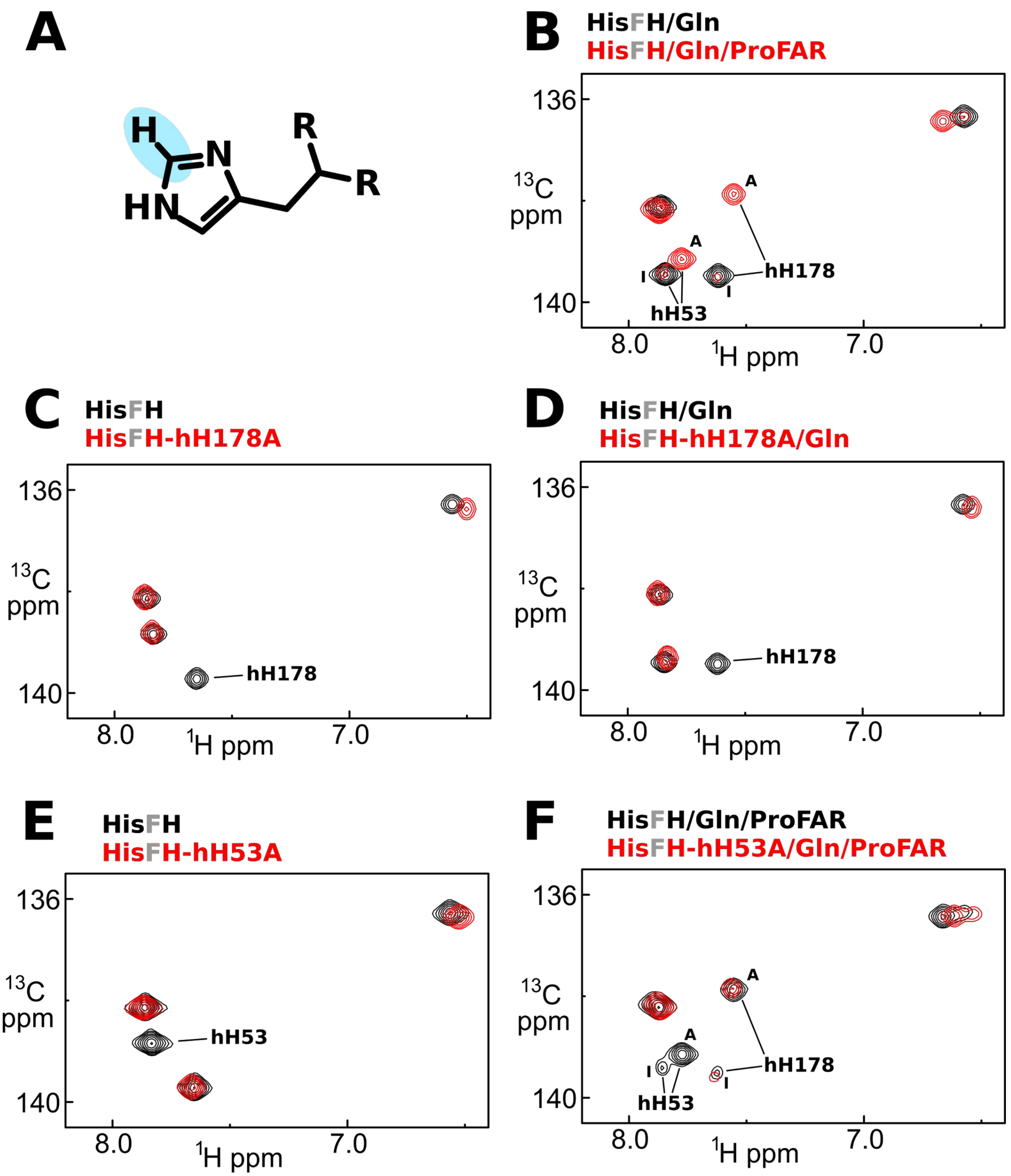

Supplementary Fig. 3 | Assignment of the catalytic histidine hH178 in HisH

*(**A**) The ^{1}H2-^{13}C2 correlation that is observed in the NMR spectra is indicated by a blue ellipse on the histidine structure.*

*(**B**) ^{1}H-^{13}C-HMQC spectra of the aromatic region are shown (HisF ^{2}H^{15}N-labeled, HisH ^{2}H, ring-^{1}H2/^{13}C2 histidine labeled). Only the ^{1}H2-^{13}C2 of signals of HisH histidine are visible, HisF is invisible in the spectra (indicated by a gray F on the top). Black spectrum: HisFH-hC84S/Gln complex, red spectrum: HisFH-hC84S/Gln/ProFAR complex, signals of the active (A) and inactive (I) conformation are labeled and the signals of hH53 and hH178 are indicated.*

*(**C**) Assignment of hH178 in the apo state: ^{1}H-^{13}C-HMQC spectra of the aromatic region are shown (HisF $^{2}H^{15}N$-labeled, HisH ^{2}H, ring-$^{1}H2$/$^{13}C2$ histidine labeled) for HisFH-hC84S (black) and HisFH-hC84S/hH178A (red). The signal of hH178 is missing in the hH178A background.*

*(**D**) Assignment of hH178 in the Gln-bound state: ^{1}H-^{13}C-HMQC spectra of the aromatic region are shown (HisF $^{2}H^{15}N$-labeled, HisH ^{2}H, ring-$^{1}H2$/$^{13}C2$ histidine labeled) for HisFH-hC84S (black) and HisFH-hC84S/hH178A (red) (both spectra were recorded in the presence of Gln). The signal of hH178 is missing in the hH178A background.*

*(**E**) Assignment of hH53 in the apo state: ^{1}H-^{13}C-HMQC spectra of the aromatic region are shown (HisF $^{2}H^{15}N$-labeled, HisH ^{2}H, ring-$^{1}H2$/$^{13}C2$ histidine labeled) for HisFH-hC84S (black) and HisFH-hC84S/hH53A (red). The signal of hH53 is missing in the hH53A background.*

*(**F**) Assignment of hH53 in the Gln/ProFAR bound state: ^{1}H-^{13}C-HMQC spectra of the aromatic region are shown (HisF $^{2}H^{15}N$-labeled, HisH ^{2}H, ring-$^{1}H2$/$^{13}C2$ histidine labeled) for HisFH-hC84S (black) and HisFH-hC84S/hH53A (red) (both spectra were recorded in the presence of Gln and ProFAR). The active conformation is formed. The signals of the active (A) and inactive (I) conformation are labeled and the signals of hH53 and hH178 are indicated. The signals of hH53 is missing in the hH53A background. As only hH53 and hH178 show large CSP in the active conformation, the assignment of the hH53 signals allows for the assignment of the hH178 signal in the active conformation.*

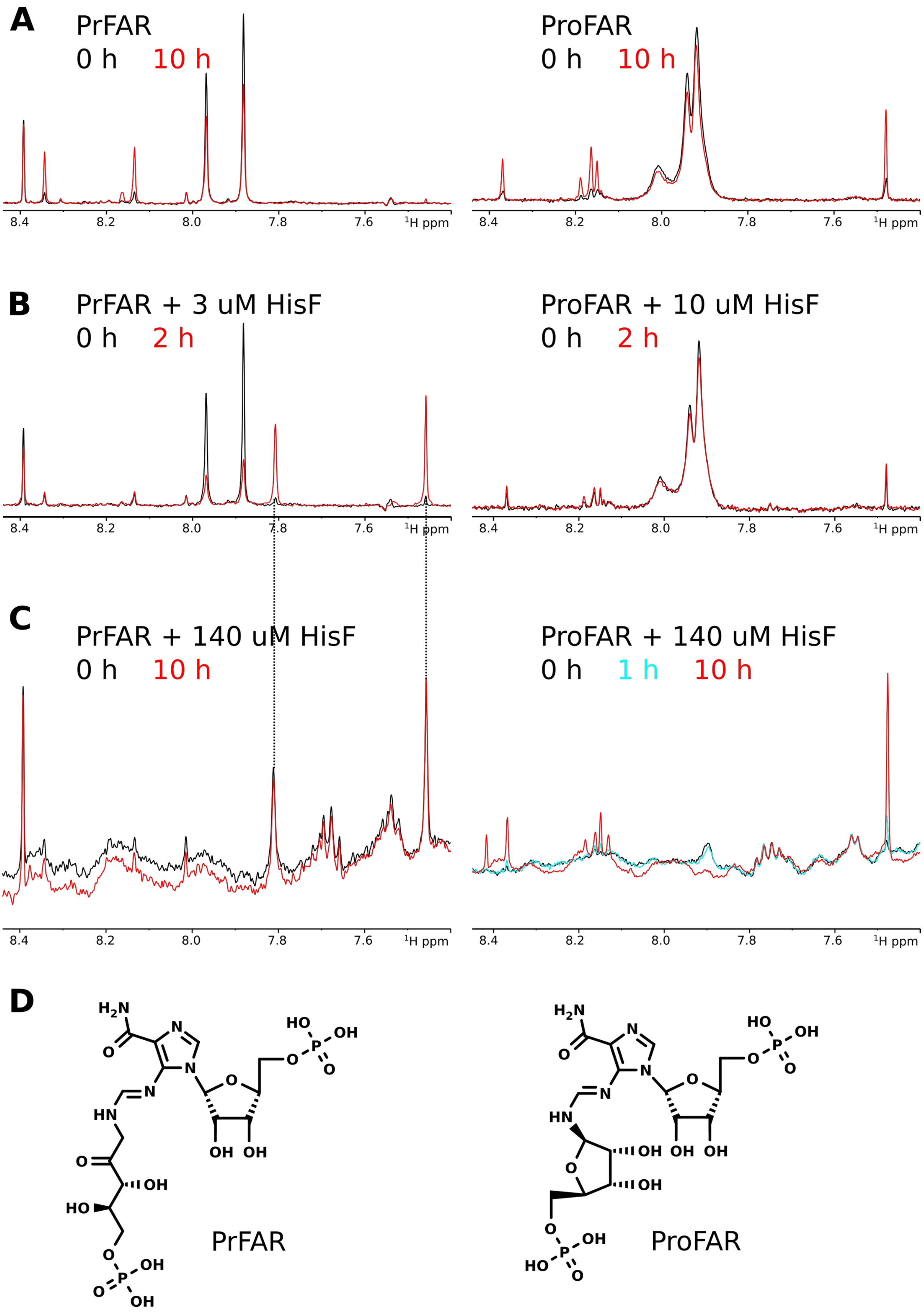

A
PrFAR
0 h 10 h
ProFAR
0 h 10 h
B
PrFAR + 3 uM HisF
0 h 2 h
ProFAR + 10 uM HisF
0 h 2 h
C
PrFAR + 140 uM HisF
0 h 10 h
ProFAR + 140 uM HisF
0 h 1 h 10 h
8.4
8.2
8.0
7.8
7.6
¹H ppm
D
H2N
N
O
HN
HO
OH
P
PrFAR
ProFAR

Supplementary Fig. 4 | Stability of 500 µM PrFAR (left) and 500 µM ProFAR (right) in NMR buffer at 30 °C followed by 1D-^{1}H NMR.

*(**A**) Spectra of PrFAR (left) and ProFAR (right) in the absence of HisF before (black) and after incubation for 10 h (red). Most of PrFAR and ProFAR are still intact after 10 h, but some degradation products are observed. Note that the line width of the ProFAR signals is much larger than the line width of the PrFAR signals. This is most likely due to exchange processes in the ProFAR molecule. The ProFAR spectra have been re-scaled to facilitate the comparison with PrFAR.*

*(**B**) PrFAR degradation is dramatically enhanced in the presence of 3 µM HisF (left). Most PrFAR is degraded already after 2 h incubation in the presence of 3 µM HisF. Note that different degradation products are formed in the presence of HisF (see signals at 7.45/7.82 ppm) compared to the incubation in the absence of HisF. In contrast ProFAR stability is only slightly decreased in the presence of 10 µM HisF (right).*

*(**C**) PrFAR degradation is too fast to be followed by NMR in the presence of 140 µM HisF (left). The first spectrum after addition of HisF (black) already shows exclusively degradation products of PrFAR (compare to (B)), no signals of intact PrFAR are observable (left). No further changes are observed after incubation for 10 h. In contrast ProFAR (right) is stable for several hours in the presence of 140 µM HisF. After 1 h (cyan) only small amounts of degradation products are observed (see signal at 7.48 ppm), after 10 h most of the ProFAR is degraded (red). Note that 140 µM HisFH is a concentration that is required for detailed NMR studies of the enzyme complex.*

*(**D**) Structures of PrFAR (left) and ProFAR (right).*

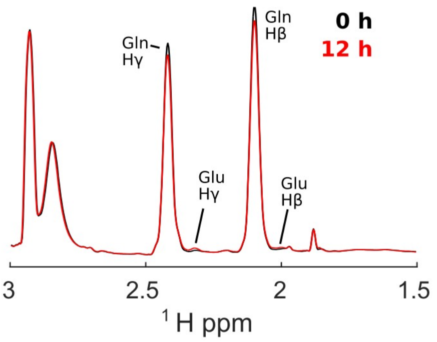

Supplementary Fig. 5 | The HisFH-hC84S mutant complex shows very low glutaminase activity in the presence of ProFAR.

Initial 1D-^{1}H spectrum (black) and spectrum recorded after incubation at 30 °C for 12 h (red) of a sample containing 180 µM HisFH-C84S complex, 20 mM Gln and 500 µM ProFAR in NMR buffer. The Hβ and Hγ signals of Gln and Glu are labeled. Less than 5 % of the Gln is hydrolyzed during the incubation time. Based on this the upper limit for the turnover rate of the HisH-C84S mutant is 0.008 min^{-1} compared to 63.0 ± 9.6 min^{-1} for the WT complex. Nonetheless a residual glutaminase activity is clearly present and is also observed in long term measurements with ImGP at higher HisFH-C84S concentrations (see suppl. Fig. 18).

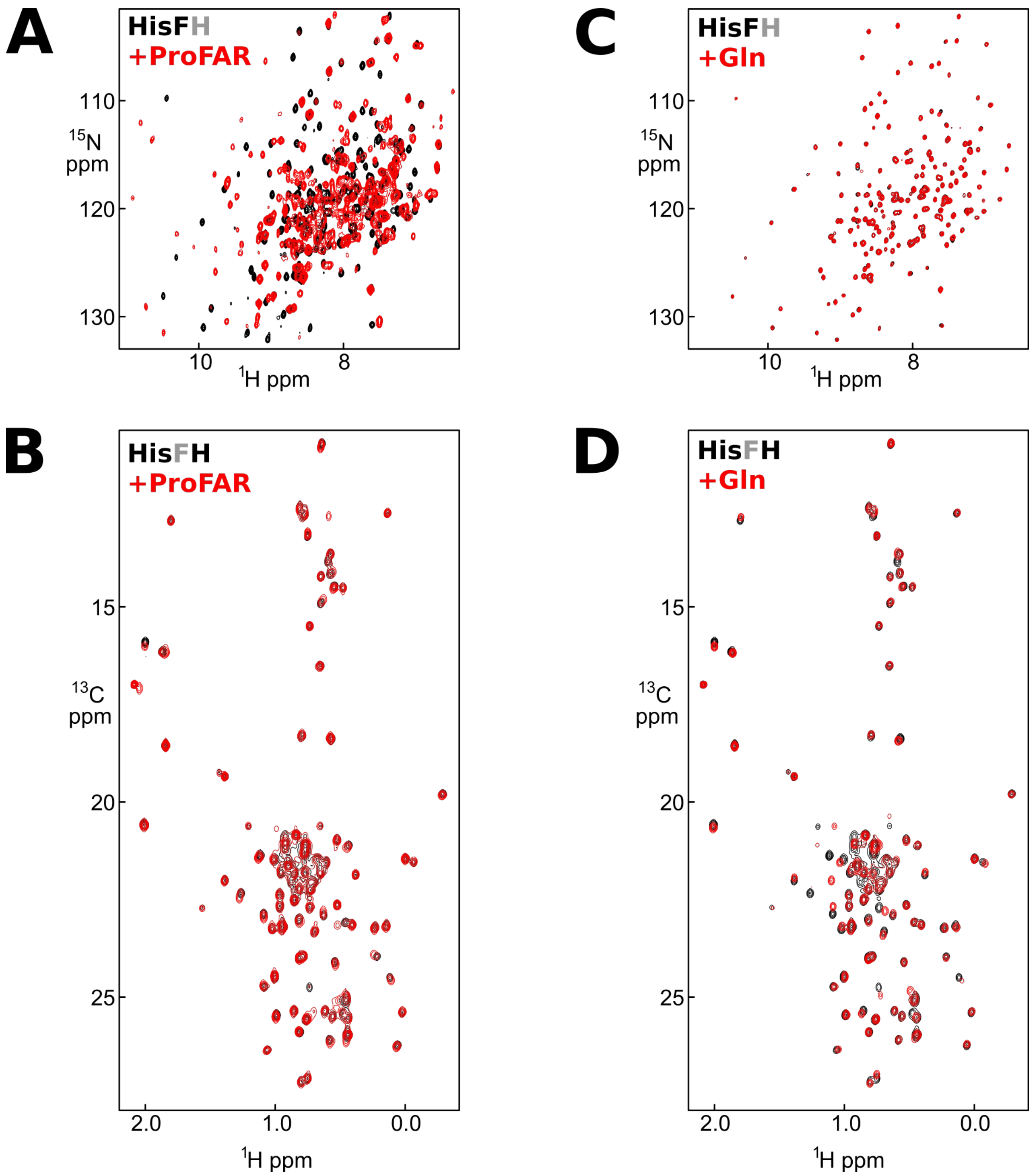

Supplementary Fig. 6 | Full NMR spectra of Gln and ProFAR titrations to WT HisFH (HisF $^{2}H^{15}N$-labeled, HisH ^{2}H, ILMVA methyl-$^{1}H^{13}C$-labeled).

*$^{1}H^{15}N$-TROSY (**A,C**; that report on HisF) and methyl-TROSY (**B,D**; that report on HisH) spectra of WT HisFH. Spectra of the apo state are shown in black, spectra in the presence of ProFAR (**A,B**) or Gln (**C,D**) are shown in red. The subunit that is not NMR visible in the respective spectrum is indicated in gray at the top.*

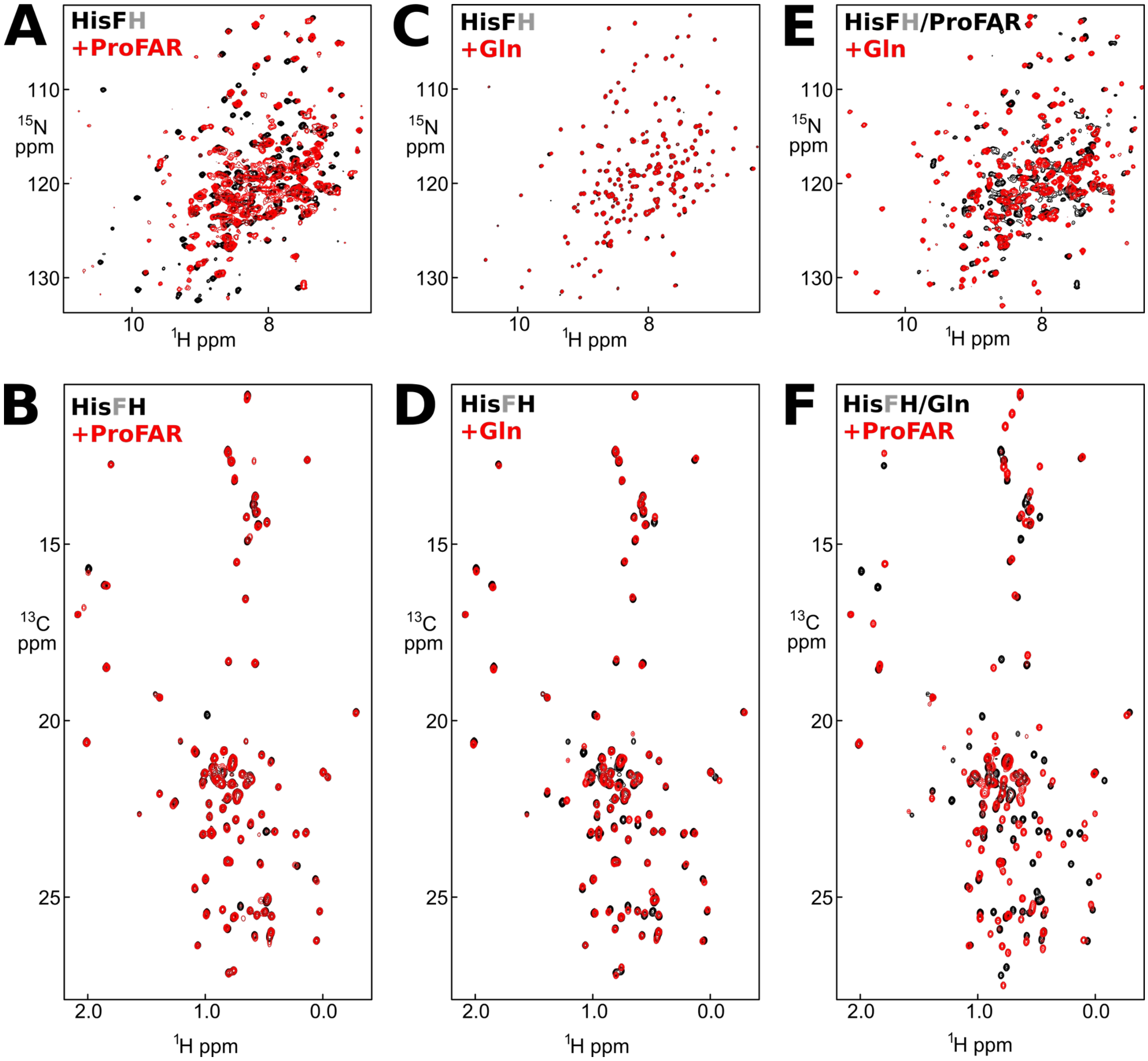

Supplementary Fig. 7 | Full NMR spectra of Gln and ProFAR titrations to HisFH-hC84S (HisF $^{2}H^{15}N$-labeled, HisH ^{2}H, ILMVA methyl-$^{1}H^{13}C$-labeled).

*$^{1}H^{15}N$-TROSY (**A,C,E**; that report on HisF) and methyl-TROSY (**B,D,F**; that report on HisH) spectra of HisFH-hC84S. (**A,B**) Spectra of the apo state (black) and ProFAR-bound (red) states. (**C,D**) Spectra of the apo state (black) and Gln bound (red) states. (**E**) Spectrum of the ProFAR bound state before (black) and after (red) addition of Gln. (**F**) Spectrum of the Gln-bound state before (black) and after (red) addition of ProFAR. The subunit that is not NMR visible in the respective spectrum is indicated in gray at the top.*

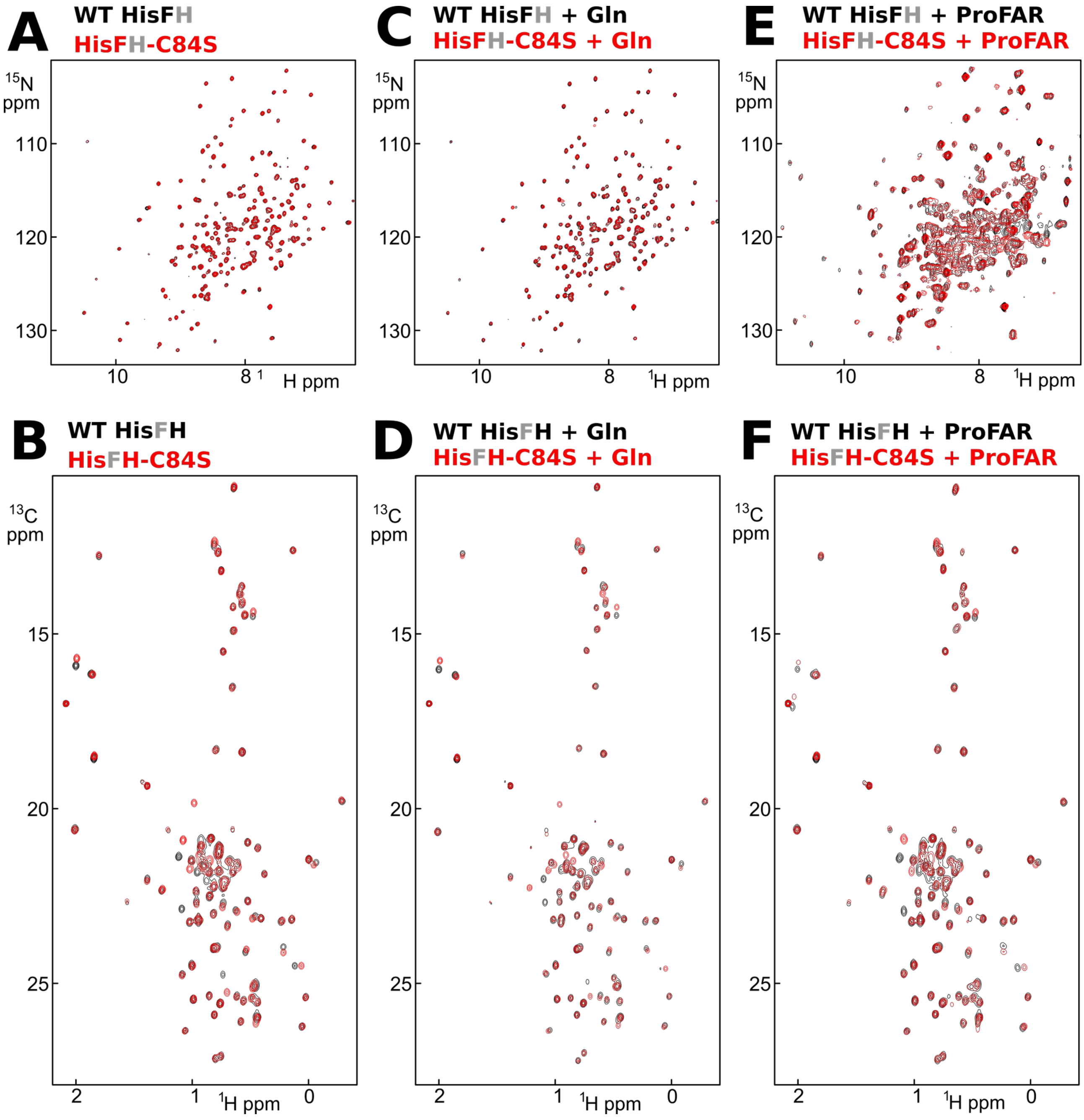

Supplementary Fig. 8 | Comparison of WT HisFH and the HisFH-C84S mutant spectra in the apo, Gln- and ProFAR-bound states (HisF $^{2}H^{15}N$-labeled, HisH ^{2}H, ILMVA methyl-$^{1}H^{13}C$-labeled).

Overlay of $^{1}H^{15}N$-TROSY (A,C,E; that report on HisF) and methyl-TROSY (B,D,F; that report on HisH) spectra of WT HisFH (black) and HisFH-hC84S (red). (A,B) Spectra of the apo state state. (C,D) Spectra of the Gln-bound state. (E,F) Spectra of the ProFAR bound state. The subunit that is not NMR visible in the respective spectrum is indicated in gray at the top.

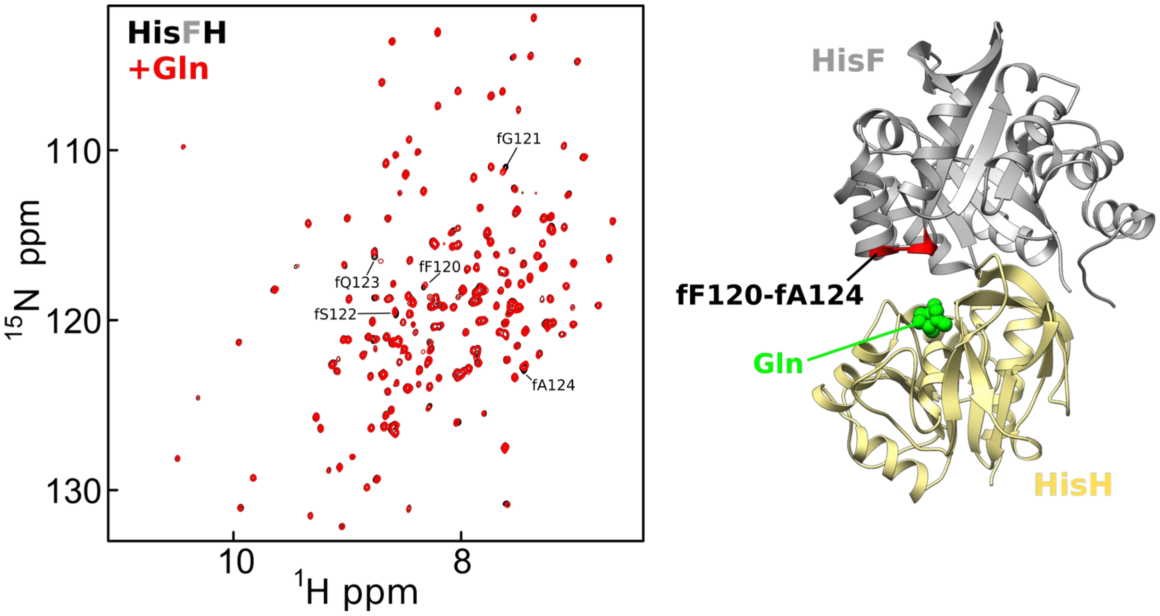

Supplementary Fig. 9 | CSP in HisF upon Gln binding to HisFH.
$^{1}H^{15}N$-TROSY spectrum of WT HisFH (HisF $^{2}H^{15}N$-labeled, HisH ^{2}H, ILMVA methyl-$^{1}H^{13}C$-labeled) in the absence (black) and presence of Gln (red) (left). Small CSPs are observed for HisF residues 120-124. These residues are located close to the Gln binding site of HisH (right). Gln is shown in green, F120-A124 in HisF are colored red (PDBID: 1GPW).

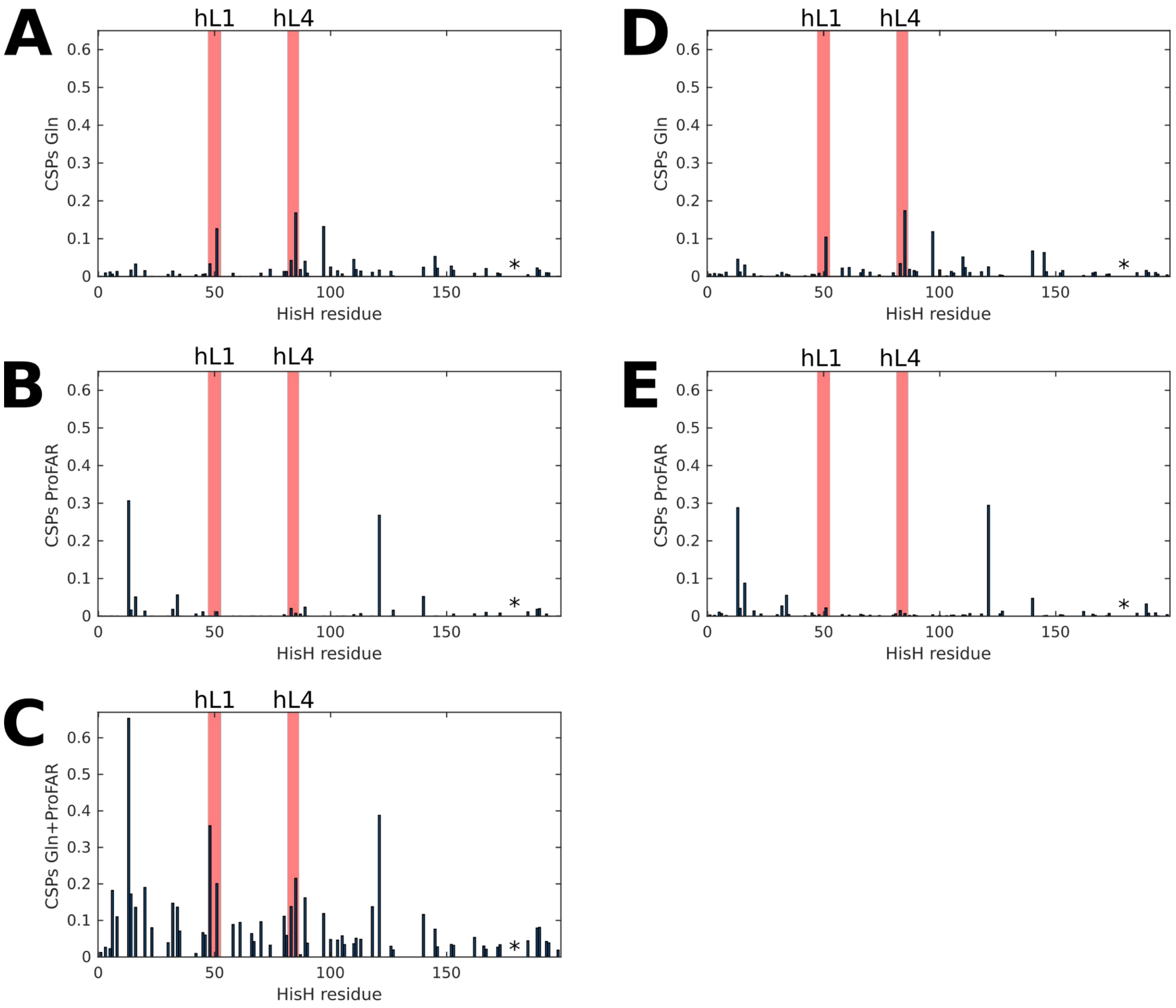

Supplementary Fig. 10 | CSPs in HisH elicited by binding of Gln, ProFAR and Gln+ProFAR to the HisFH complex

CSPs of HisH methyl groups are plotted against the residue number. ***A-C*** *shows CSPs of titrations with the HisFH-hC84S complex,* ***D-E*** *for the WT HisFH complex. In both cases HisF was $^{2}H^{15}N$-labeled and HisH ^{2}H, ILMVA methyl-$^{1}H^{13}C$-labeled.* ***A*** *and* ***D:*** *CSPs caused by Gln binding.* ***B*** *and* ***E:*** *CSPs caused by ProFAR binding.* ***C:*** *CSPs upon addition of ProFAR to the Gln bound HisFH-hC84S complex.*

hL1 and hL4 (including hC84) that form part of the active site are indicated by red bars. The region surrounding hH178 and hE180 of the catalytic triad does not contain methyl group bearing residues and is labeled with an asterisk. Note that the CSPs for the WT and hC84S complexes are virtually identical, showing that the WT and C84S proteins behave the same in the NMR titration experiments.

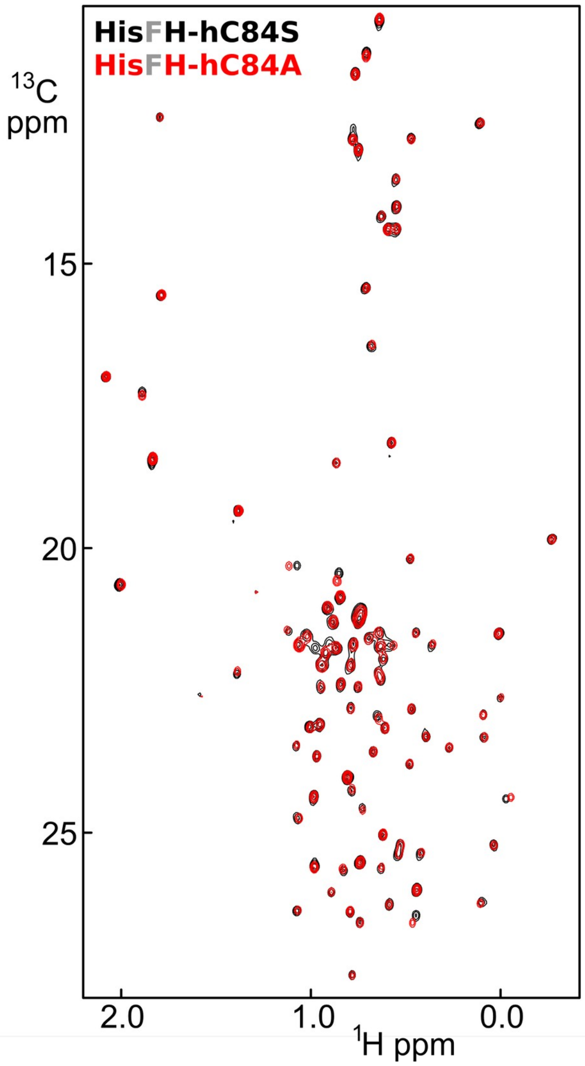

Supplementary Fig. 11 | The active conformations of HisFH-hC84S and HisFH-hC84A are virtually identical.

Methyl-TROSY spectra of HisFH-hC84S (black) and HisFH-hC84A (red) (HisF $^{2}H^{15}N$-labeled, HisH ^{2}H, ILMVA methyl-$^{1}H^{13}C$-labeled) in the presence of Gln and ProFAR are very similar. The active state conformation is thus not an artifact of the C84S or C84A mutation.

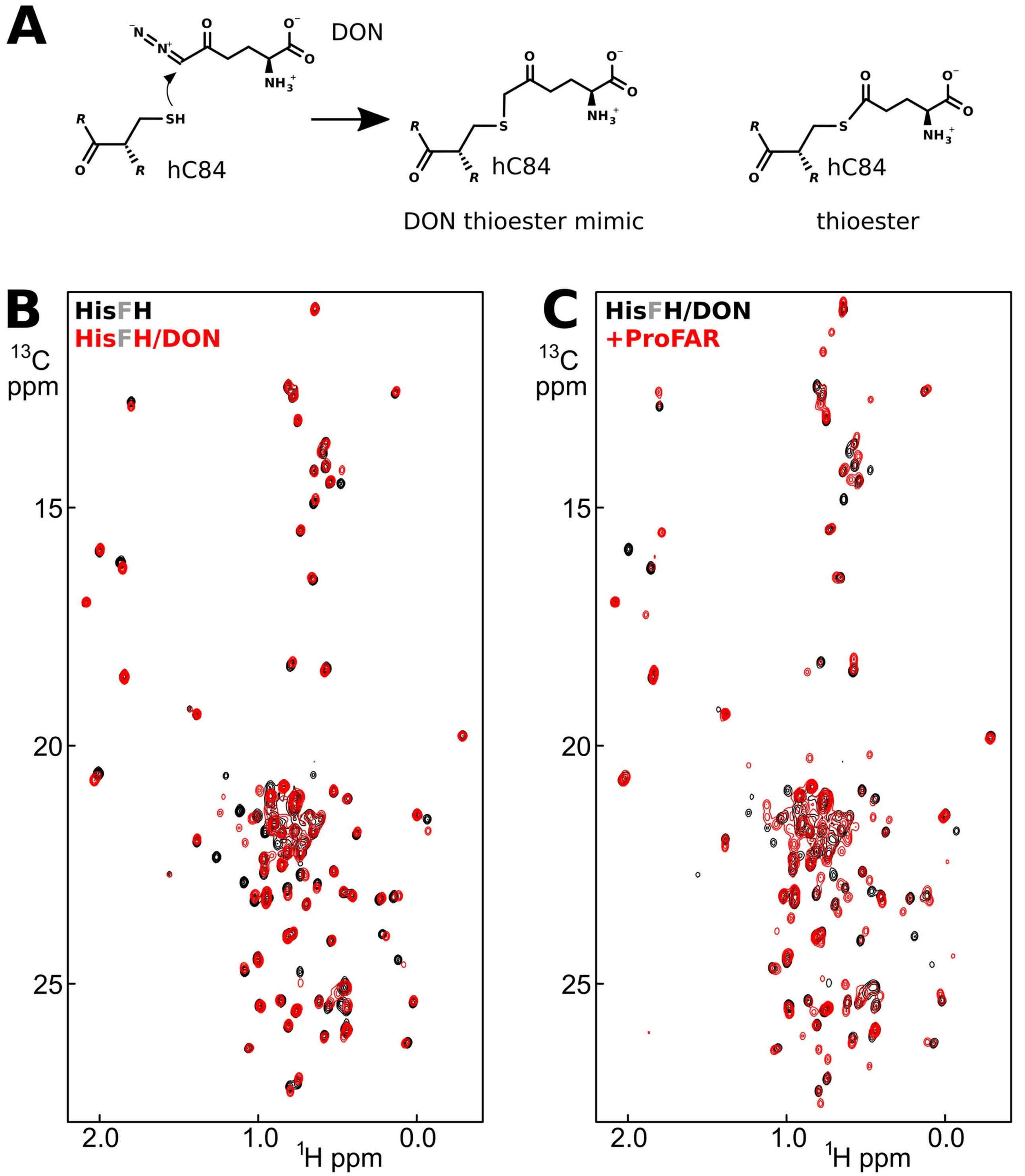

Supplementary Fig. 12 | Active conformation for the WT HisFH complex in the presence of DON and ProFAR

*(**A**) Reaction of the covalent glutaminase inhibitor DON (6-Diazo-5-oxo-L-norleucin) with the catalytic hC84 in HisH leads to the formation of a thioester mimic (left). For comparison the natural thioester intermediate is shown (right).*

*(**B**) Methyl-TROSY spectra of WT HisFH (HisF $^{2}H^{15}N$-labeled, HisH ^{2}H, ILMVA methyl-$^{1}H^{13}C$-labeled) in the apo state (black) and after reaction with DON are shown (red). The CSPs report on*

the interaction between HisH and DON and are comparable with the CSPs that are observed when WT HisFH or HisFH hC84S interact with Gln.
*(**C**) Addition of ProFAR to the HisFH/DON complex leads to partial formation of the active conformation. Methyl-TROSY spectra of the WT HisFH/DON complex in the absence (black) and presence (red) of ProFAR are shown. The CSPs are comparable to the CSPs that are observed when ProFAR is added to the Gln saturated hC84S HisFH complex (Suppl. Fig. 7F).*

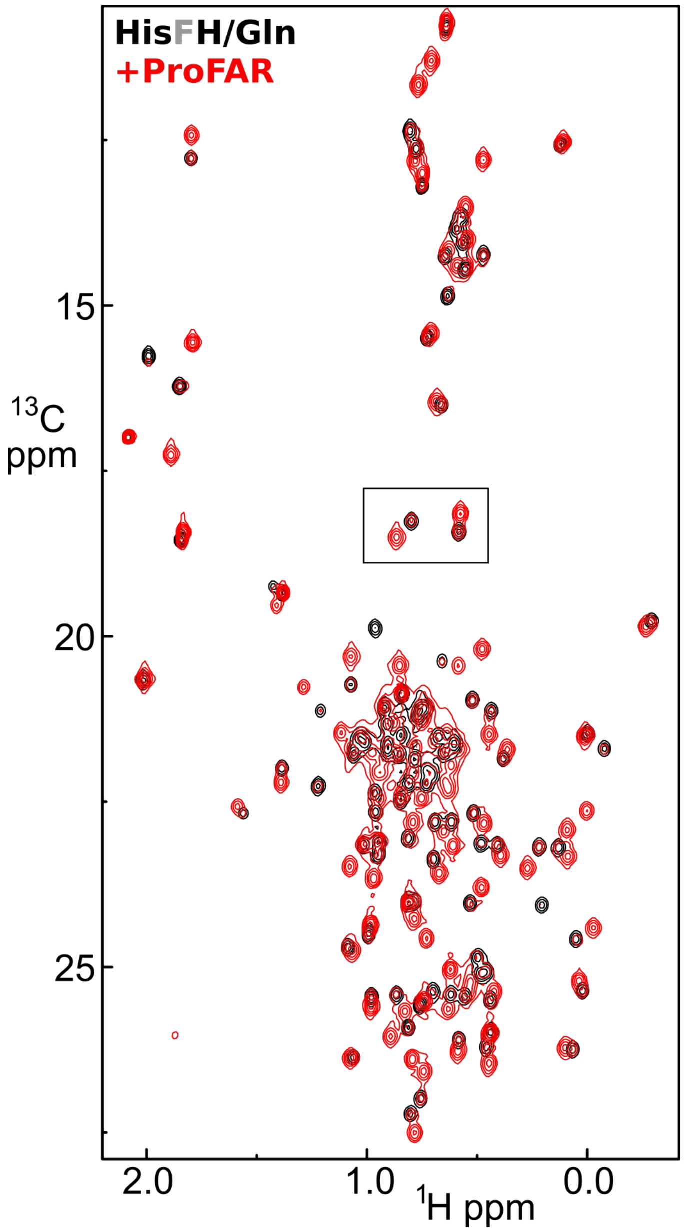

Supplementary Fig. 13 | Equilibrium between inactive and active conformation for the HisFH-hC84S complex in the presence of Gln and ProFAR

Methyl-TROSY spectra of HisFH-hC84S (HisF $^{2}H^{15}N$-labeled, HisH ^{2}H, ILMVA methyl-$^{1}H^{13}C$-labeled) in the presence of 20 mM Gln (black) and after addition of 0.4 mM ProFAR are shown (red). Only HisH is visible in the spectrum. Strong CSPs report on the formation of the active conformation, but weak signals of the inactive conformation remain present. This shows that both conformations are still populated in the Gln/ProFAR saturated HisFH complex and that the interconversion rate between both conformations is slow on the NMR timescale (<50 s^{-1}). The boxed regions correspond to the regions shown in Fig. 3 in the main text.

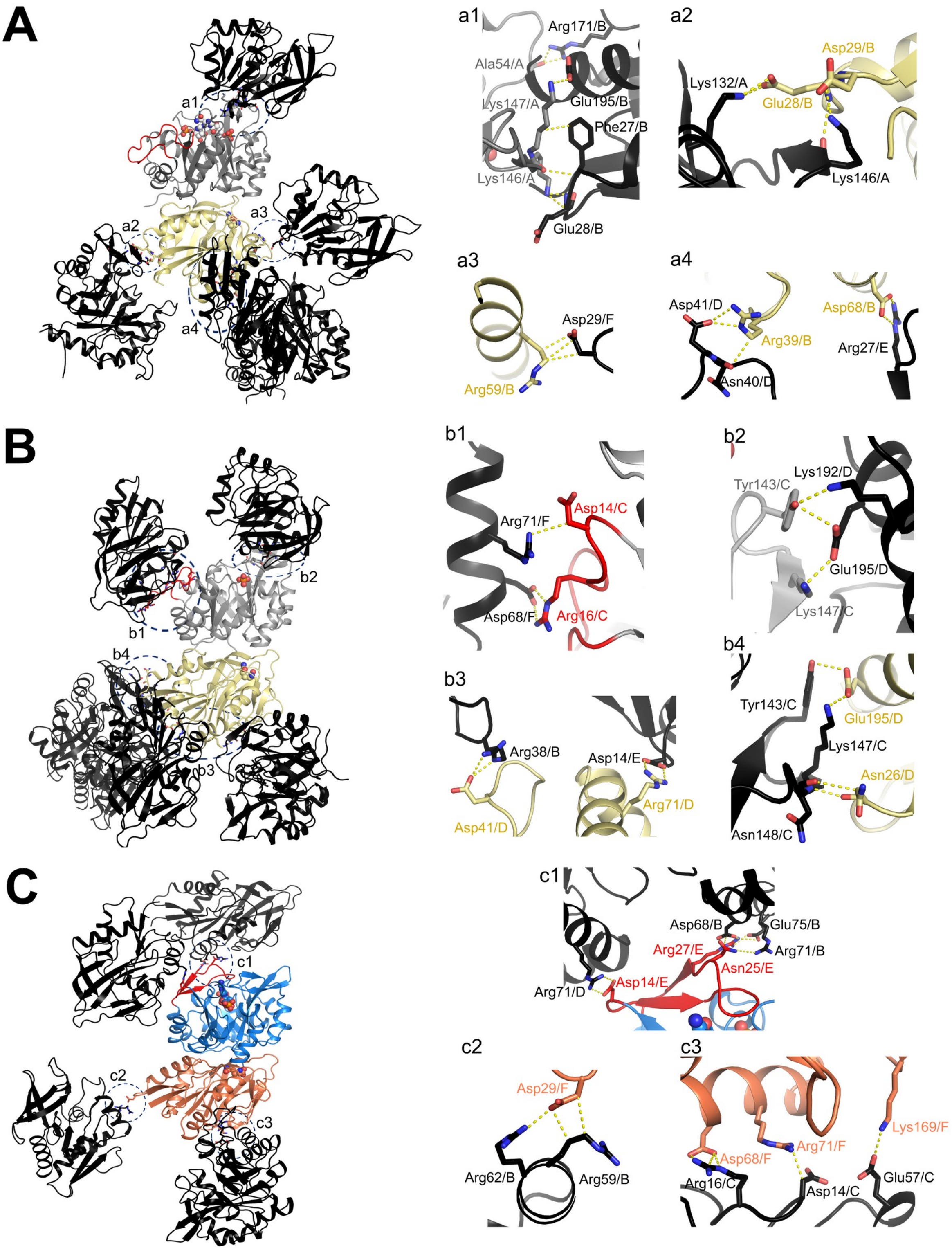

Supplementary Fig. 14 | Crystal packing

A) Crystal packing around chains A and B (that are in the ground conformation)

B) Crystal packing around chains C and D (that are in the ground conformation)

C) Crystal packing around chains E and F (that are in the active conformation)

Close-up views of the regions at the packing interface are shown on the right.

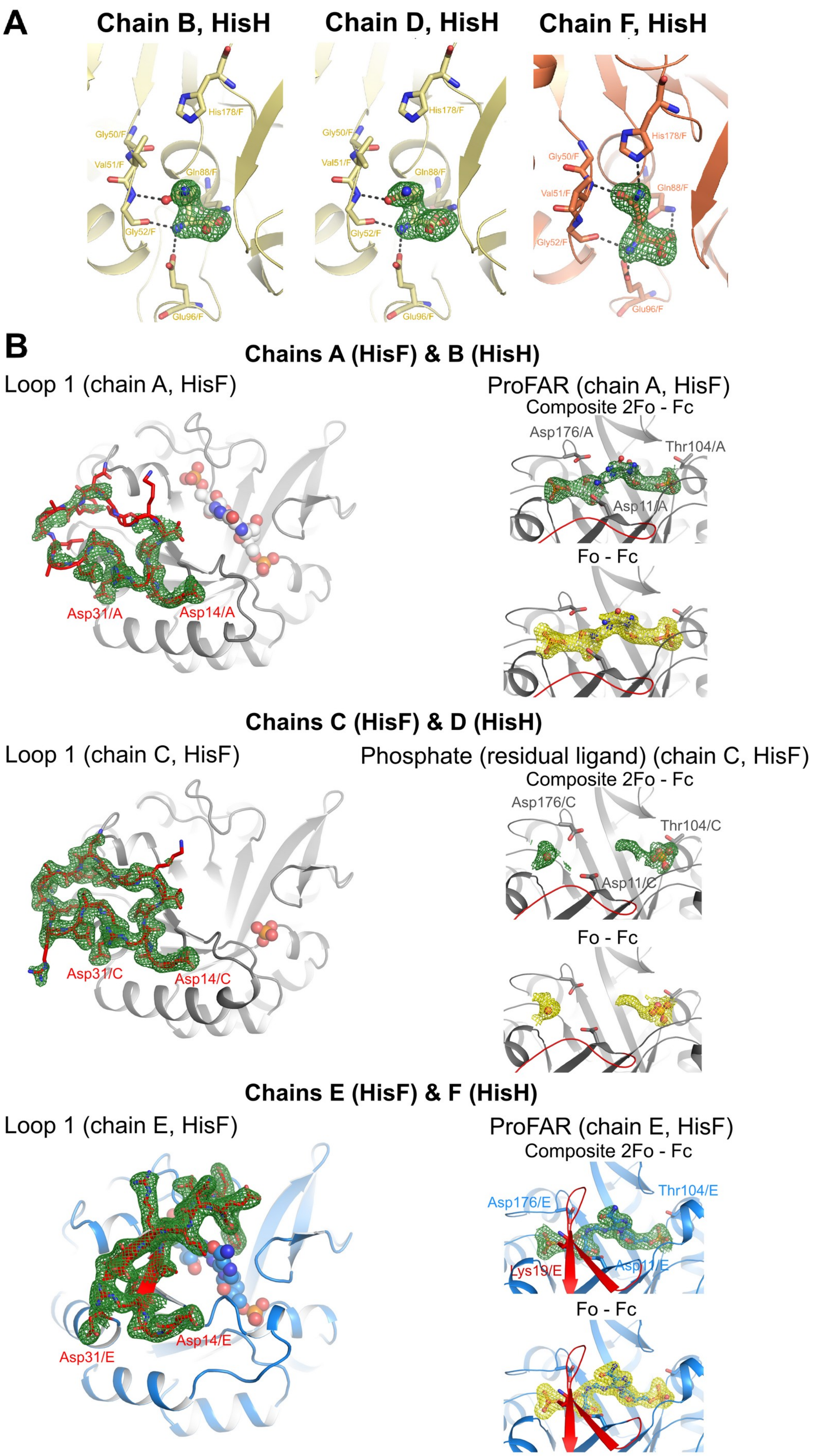
A
Chain B, HisH
Chain D, HisH
Chain F, HisH
His178/F
Gly50/F
Val51/F
Gln88/F
Gly52/F
Glu96/F
B
Chains A (HisF) & B (HisH)
Loop 1 (chain A, HisF)
Asp31/A
Asp14/A
ProFAR (chain A, HisF)
Composite 2Fo - Fc
Asp176/A
Thr104/A
Asp11/A
Fo - Fc
Chains C (HisF) & D (HisH)
Loop 1 (chain C, HisF)
Asp31/C
Asp14/C
Phosphate (residual ligand) (chain C, HisF)
Composite 2Fo - Fc
Asp176/C
Thr104/C
Asp11/C
Fo - Fc
Chains E (HisF) & F (HisH)
Loop 1 (chain E, HisF)
Asp31/E
Asp14/E
ProFAR (chain E, HisF)
Composite 2Fo - Fc
Asp176/E
Thr104/E
Lys19/E
Asp11/E
Fo - Fc

Supplementary Fig. 15 | Electron density maps

(A) Composite omit 2Fo - Fc electron density maps for Glutamine in the HisH active site, contoured at 1.0 σ. Hydrogen bonds are shown as dotted black lines.

(B) Composite omit 2Fo - Fc maps contoured at 1.0 σ are shown as a green mesh for loop 1 in HisF (left panels) and for ligands at the ProFAR binding site in HisF (right upper panels). Fo-Fc difference maps contoured at 3.0 σ are shown as a yellow mesh (right lower panel). The color codes of the cartoons are same as in Figure 2.

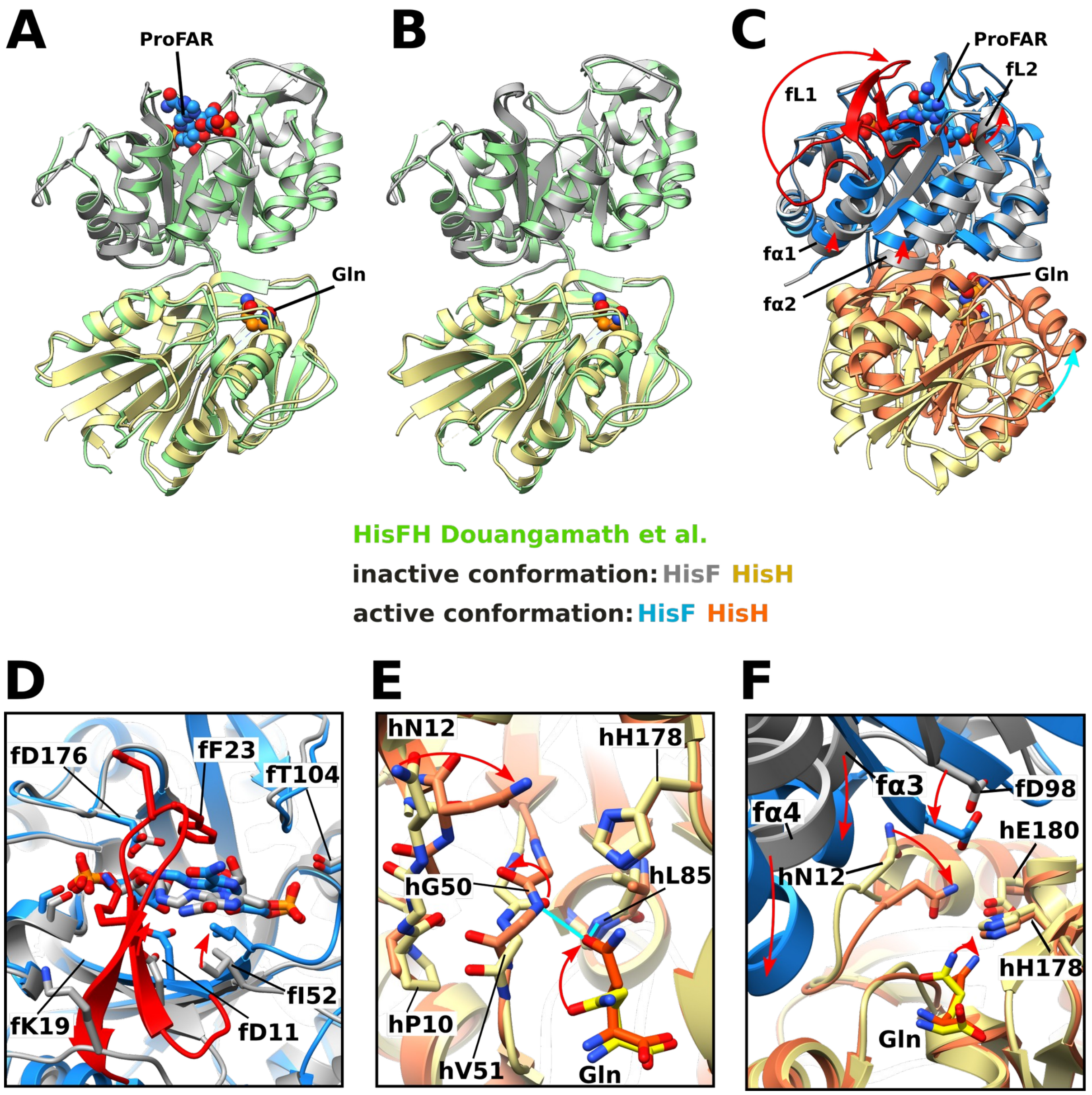

Supplementary Fig. 16 | Comparison between the apo HisFH structure from Douangamath et al. [2] ***and the structures of the inactive conformation determined in this publication and direct comparison between the structures in the active and inactive conformation.***

*(**A,B**) The structure of the apo HisFH complex from Douangamath et al. (PDB 1GPW, chains E/F, shown in green) was superposed with the structures of the inactive and active conformations that we determined here. HisF and HisH of our structures are colored gray and khaki for the inactive conformation and blue and orange for the active conformation. ProFAR (blue) and Gln (orange) are shown in ball representation. Structures are superimposed based on HisF. (**A**) Comparison of the apo structure with the structure in the inactive conformation bound to ProFAR and Gln (chains A/B). (**B**) Comparison of the apo structure with the structure in the inactive conformation bound to Gln (chains C/D)*

*(**C-F**) Direct comparison between the structures determined in this publication (as in Fig. 2 of the main manuscript; PDBID: 7AC8). Structures are overlayed instead of shown side by side to allow for a more direct comparison. (**C**) Overlay of the structure of the HisFH complex (hC84A mutation) in the inactive conformation bound to Gln with the structure in the active conformation bound to*

*ProFAR and Gln. Structures are superimposed on based on HisF. The largest structural differences between the two conformations are observed in HisF (red arrows) (fL1 (red), fL2, fα1 and fα2). The reorientation of HisH relative to HisF is indicated by a cyan arrow. (**D**) Closeup of the HisF active site: Overlay of HisF in the inactive conformation bound to ProFAR and Gln and in the active conformation. Important hydrogen bond interactions are shown in green. fL1 is shown in red. The compaction of the HisF active site in the active conformation is indicated by red arrows. (**E**) Oxyanion hole formation in the active conformation of HisH: Overlay of the structures of HisH in the inactive conformation bound to ProFAR and Gln and in the active conformation. The oxyanion hole is not formed in the inactive conformation. Red arrows indicate important conformational changes upon formation of the active conformation. The oxyanion hole is formed by the rotation of the hG50/hV51 amide group, which also leads to the formation of a new hydrogen-bond to hN12. The side chain of the substrate Gln rotates into the oxyanion hole and forms hydrogen bonds to hG50 and hL85 (shown in cyan). (**E**) Overlay of the structures of the inactive conformation bound to ProFAR and Gln and of the active conformation. Structures are superimposed on based on HisH. The rearrangement of the HisF-HisH interface moves fα3/α4 and fD98 closer to the active site (movements are indicated by red arrows). Upon transition into the active conformation (right) fD98 moves by 3.6 Å and interacts with hN12.*

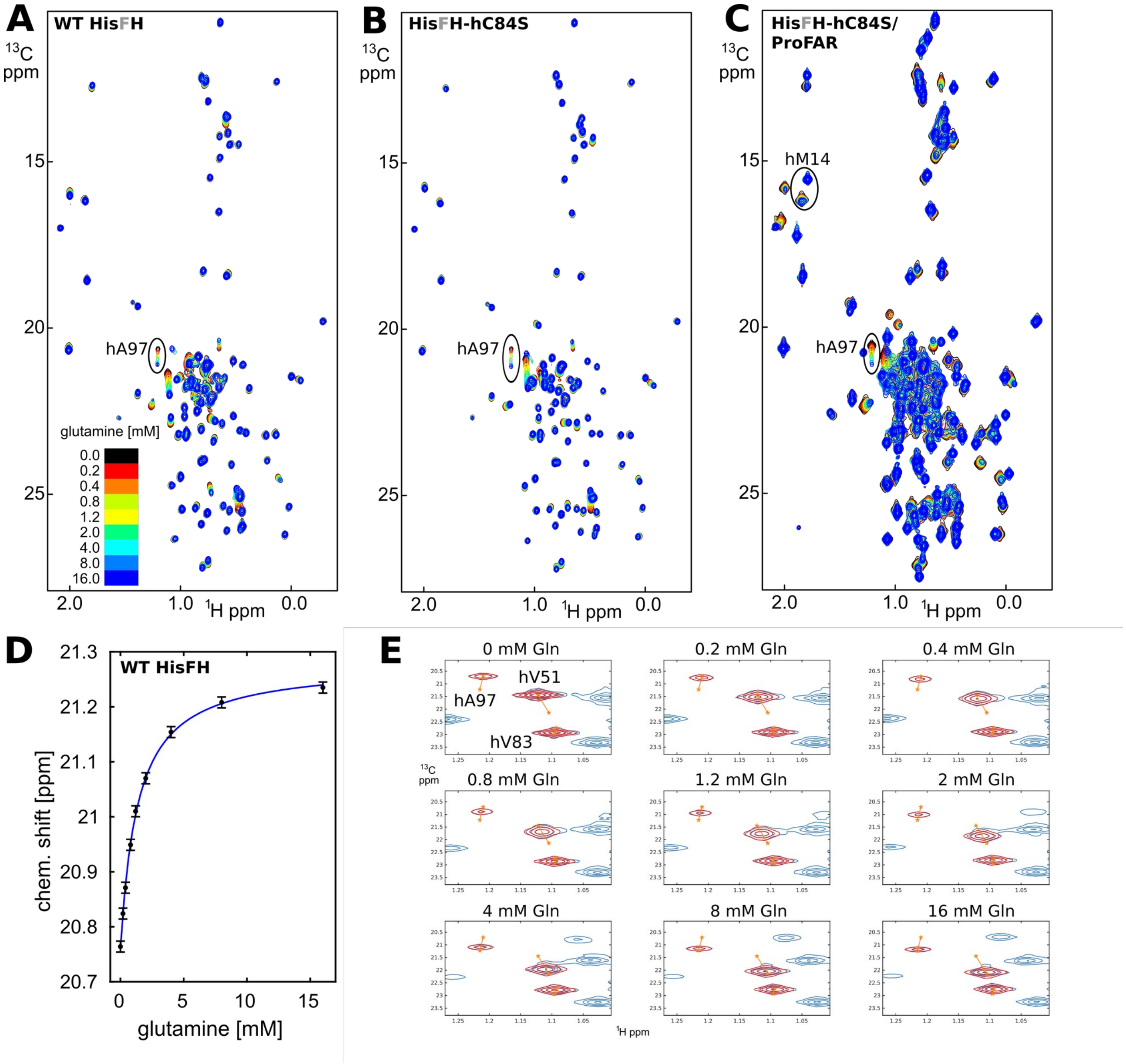

Supplementary Fig. 17 | Gln titrations to the HisFH complex.

*(**A**) Gln titration to WT HisFH in the absence of ProFAR (HisF $^{2}H^{15}N$-labeled, HisH ^{2}H, ILMVA methyl-$^{1}H^{13}C$-labeled). Methyl-TROSY spectra were recorded after addition of 0-16 mM Gln (black to blue, see color legend). The signal of hA97 is marked by an ellipse.*

*(**B**) same as A for HisFH-hC84S*

*(**C**) Gln titration to HisFH-hC84S in the presence of ProFAR shown at lower contour levels due to the low intensity of the inactive conformation signals at higher Gln concentrations. Colors as in (A)*

*(**D**) Fitting of the Gln titration to WT HisFH in the absence of ProFAR (for full spectrum see (A)). The ^{13}C chemical shift of hA97 is plotted against the Gln concentration and fitted to a two-state model binding model (blue line). The Gln K_D obtained from the fit is 1.3 (± 0.1) mM. Data are presented as mean values ± 1 standard deviation (corresponding to the spectral resolution). Source data are provided as a Source Data file.*

*(**E**) Exemplary 2D-lineshape analysis of Gln binding to WT HisFH (HisF $^{2}H^{15}N$-labeled, HisH ^{2}H, ILMVA methyl-$^{1}H^{13}C$-labeled). Sections of the methyl-TROSY spectra during the Gln titrations are shown (blue contours). Fits to the signals of hA97, hV51 and hV83 (red contours) using a two-state binding model in TITAN yield a k_{off} of 1600 ± 200 s^{-1}.*

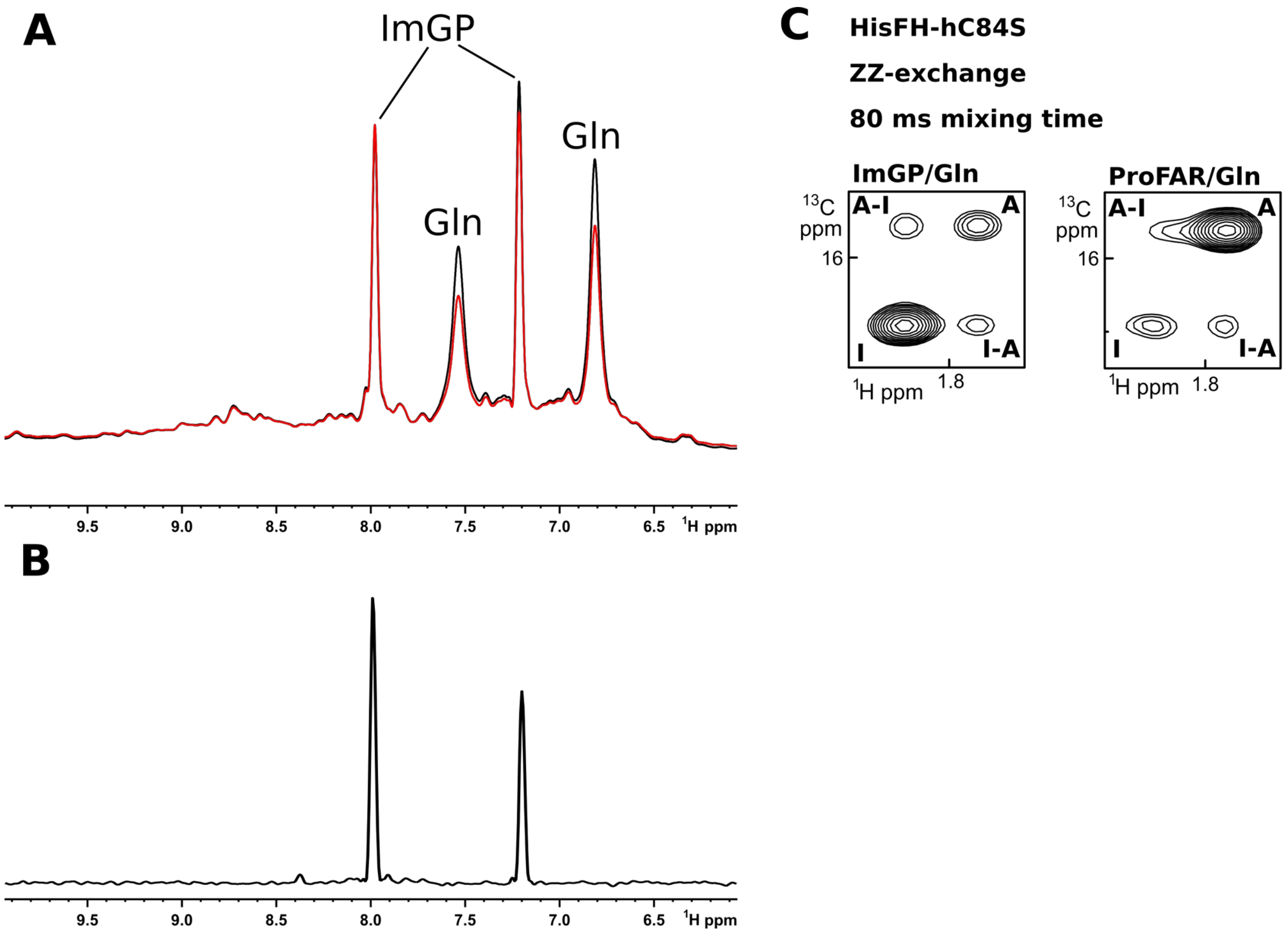

Supplementary Fig. 18 | ImGP is stable during longitudinal ZZ-exchange experiments.
*(**A**) 1D-^{1}H NMR spectra were recorded before (black) and after the ZZ-exchange experiment (~2.5 days) in the presence of ImGP (10 mM) and Gln (40 mM) for HisFH-hC84S. ImGP and Gln peaks are indicated. The residual glutaminase activity of the HisFH-C84S complex leads to partial degradation of Gln; ImGP is stable (for ProFAR stability see Suppl. Fig 4).*
*(**B**) 1D-^{1}H NMR spectrum of ImGP in NMR buffer at 30 °C for comparison.*
*(**C**) Comparison of longitudinal ZZ-exchange experiments for HisFH-hC84S in the presence of ImGP/Gln (left) and ProFAR/Gln (right). Closeup of the hM121 signals of the inactive (labeled I) and active conformation (labeled A) for a mixing time of 80 ms. The exchange peaks that originate from the interconversion between inactive and active conformation (A-I and I-A) are indicated. The intensities of the exchange peaks between the two spectra are comparable indicating that the exchange between active and inactive conformation in the presence of ProFAR/Gln takes place on a similar timescale as in the presence of ImGP/Gln. Due to the low stability of ProFAR only one spectrum with a single mixing time can be recorded for the ProFAR/Gln-bound sample thus preventing the precise determination of the exchange rates.*

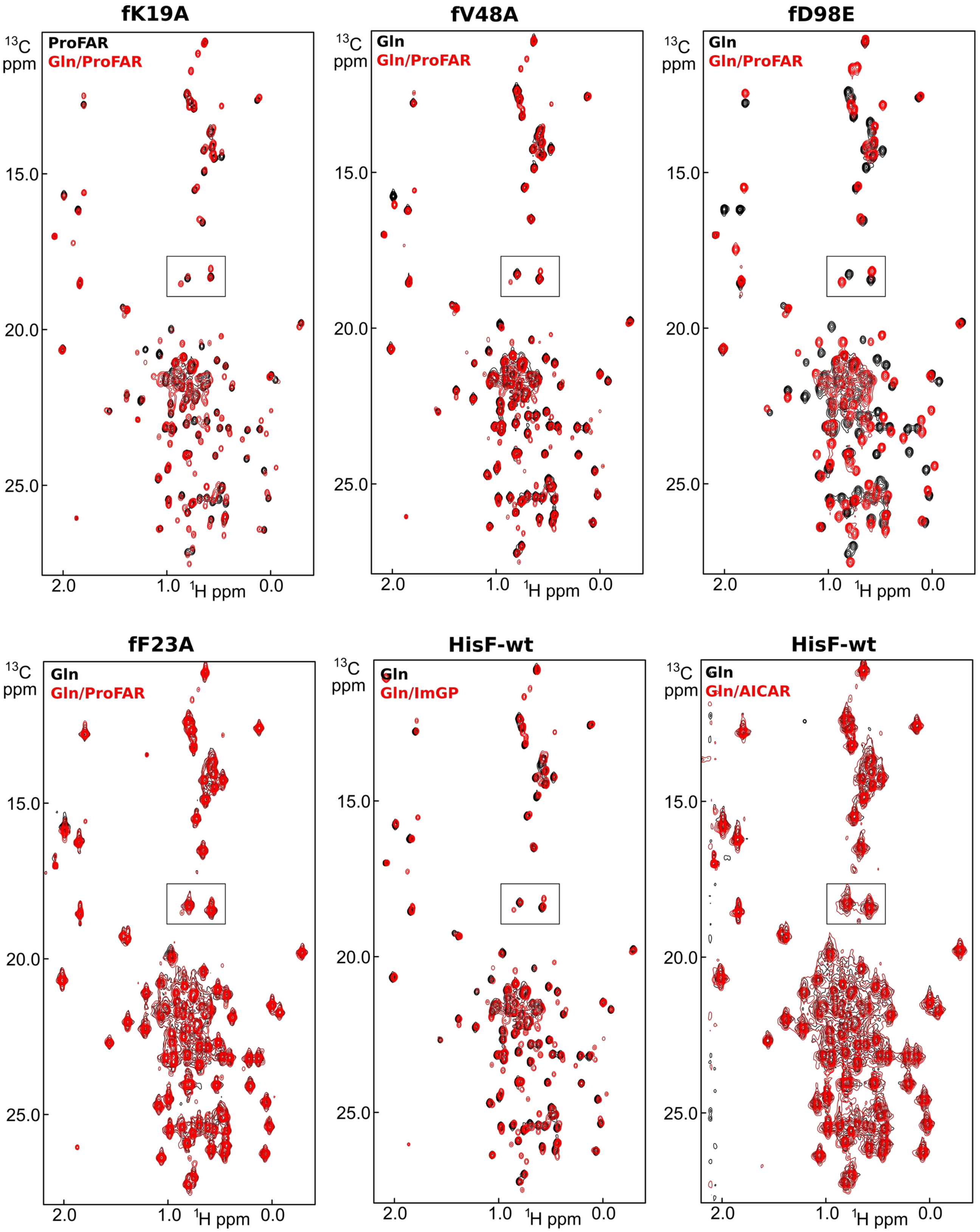

Supplementary Fig. 19 | The population of the active conformation of HisFH is modulated by different activators or mutations in HisF.

Methyl-TROSY spectra of HisFH-hC84S (HisF $^{2}H^{15}N$-labeled, HisH ^{2}H, ILMVA methyl-$^{1}H^{13}C$-labeled) for WT HisF and different mutants in the presence of saturating concentrations of Gln

(black) and after addition of activator (AICAR, ImGP or ProFAR). The contour levels are chosen such that the signals of the active conformation are visible. The boxed regions highlight the signals of hV8 and hV111 and correspond to the regions shown in Fig. 3 in the main text. Note that the population of the active conformations is modulated by the various activators and mutations, however, the structures of the active conformations are basically identical as evidenced by the identical HisH spectra of the active conformations.
*(**A**) HisFH-fK19A/ProFAR titrated with Gln (Note that here the black spectrum corresponds to the ProFAR bound complex and the red spectrum to the Gln/ProFAR bound complex).*
*(**B**) HisFH-fV48A/Gln titrated with ProFAR*
*(**C**) HisFH-fD98E/Gln titrated with ProFAR*
*(**D**) HisFH-fF23A/Gln titrated with ProFAR*
*(**E**) WT HisFH/Gln titrated with ImGP*
*(**F**) WT HisFH/Gln titrated with AICAR*

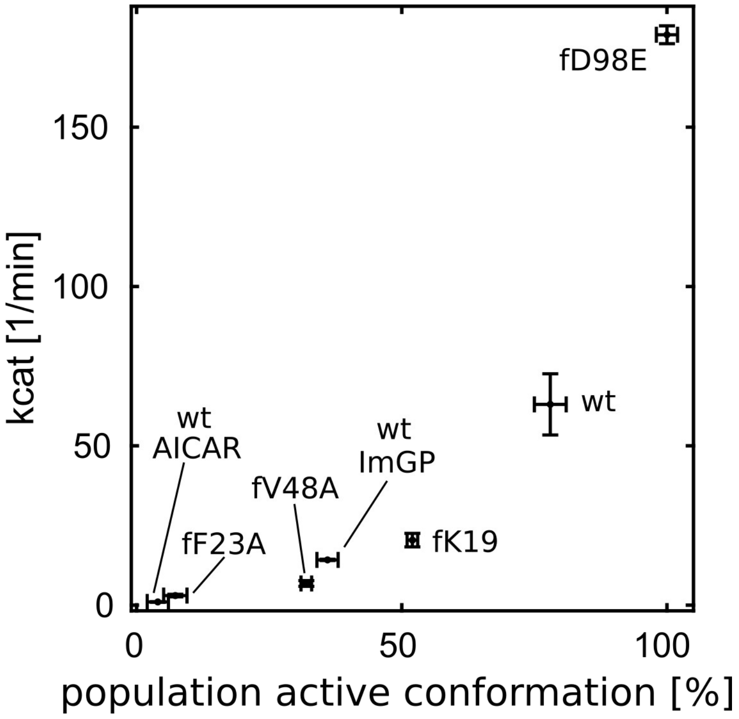

Supplementary Fig. 20 | Correlation between the k_{cat} of the HisFH glutaminase reaction and the population of the active conformation for HisF mutants and various activators.

The populations of the active conformations were determined in the HisFH-hC84S background, whereas glutaminase activity was measured on the WT HisFH complex. ProFAR was used as an activator unless indicated otherwise. Data are presented as mean values ± 1 standard deviation (n=8 resonances). Source data are provided as a Source Data file.

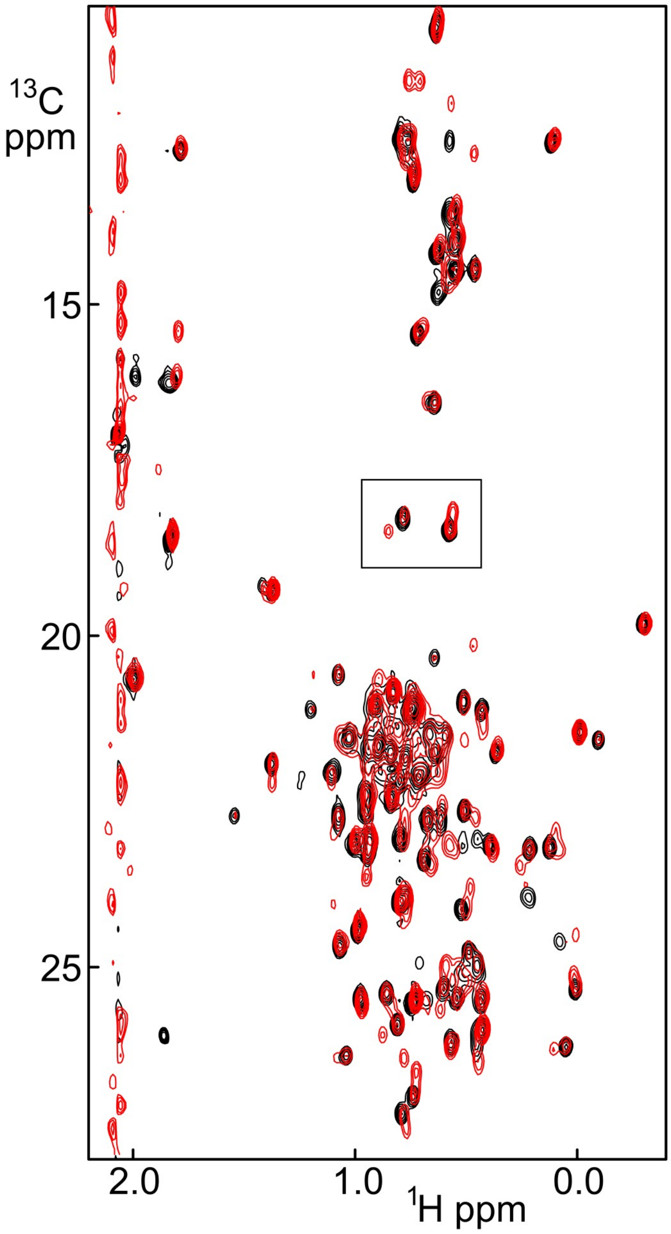

Supplementary Fig. 21 | NMR spectra under multiple turnover conditions

Methyl-TROSY spectra of WT HisFH (black) and HisFH-fD98E (red) during catalysis in the presence of 100 mM Gln and 400 µM ProFAR (HisF $^{2}H^{15}N$-labeled, HisH ^{2}H, ILMVA methyl-$^{1}H^{13}C$-labeled). The characteristic signals of the active conformation are only observed for the fD98E mutant. The artifacts around 2.1 ppm are due to t1 noise from the high concentration of Gln. The boxed region highlight the signals of hV8 and hV111 and corresponds to the region shown in Fig. 3e in the main text.

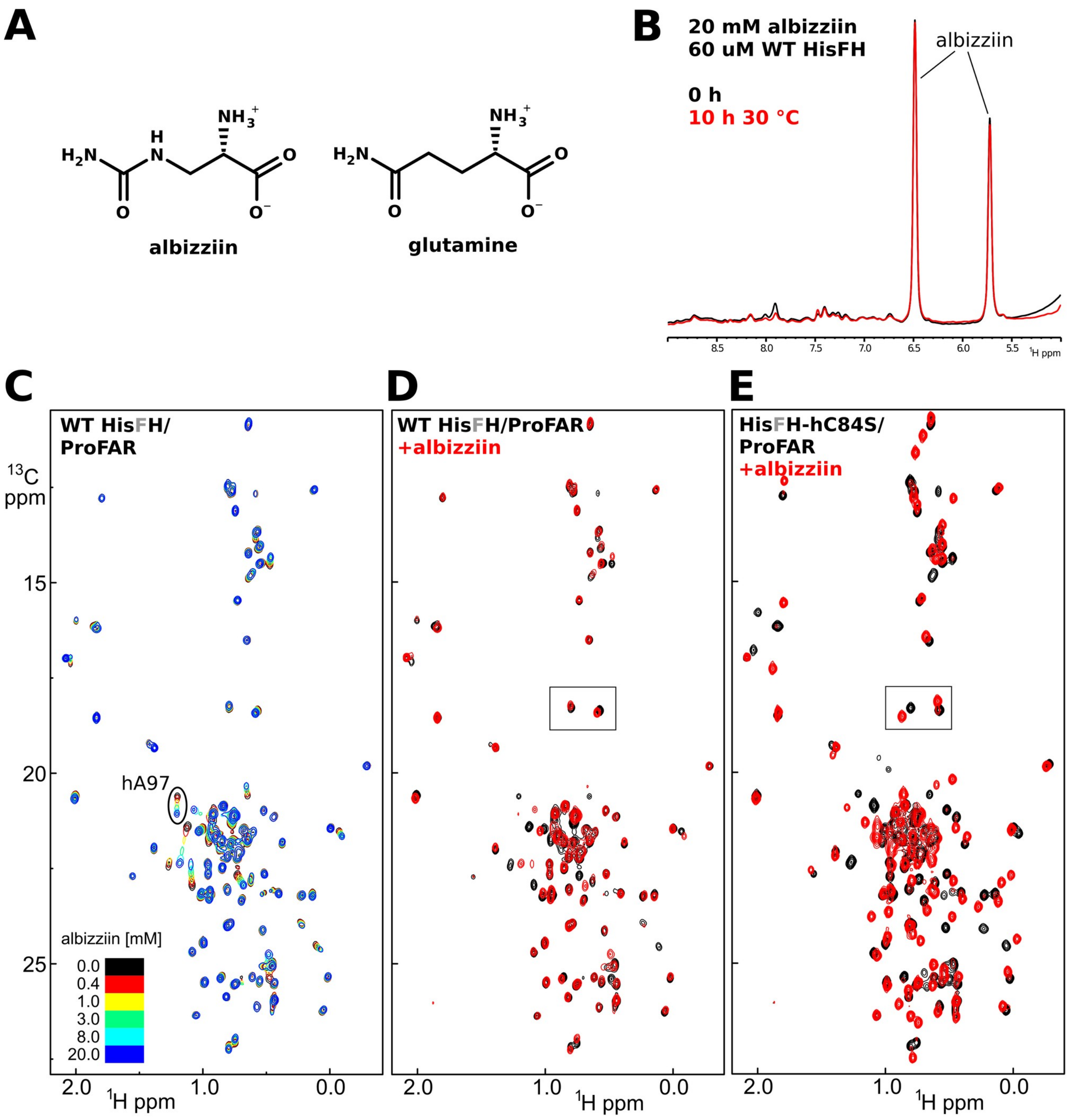

Supplementary Fig. 22 | The nonhydrolyzable Gln analog albizziin induces the formation of the active conformation only in the background of the hC84S mutation.

*(**A**) Structure of albizziin and Gln.*

*(**B**) Albizziin is not hydrolyzed by WT HisFH. 1D-^{1}H spectra of 20 mM Albizziin in NMR buffer after at 30 °C in the presence of 60 µM WT HisFH and 400 µM ProFAR (black) and after incubation for 10 h (red). The signals of the albizziin NH_2 group are indicated.*

*(**C**) Albizziin binds in fast to intermediate exchange to the WT HisFH/ProFAR complex and induces similar CSPs as Gln (compare to Suppl. Fig. 17). It is thus a reversible, competitive inhibitor of HisH. An albizziin titration to WT HisFH in the presence of ProFAR is shown (HisF ^{2}H^{15}N-labeled, HisH ^{2}H, ILMVA methyl-^{1}H^{13}C-labeled). Methyl-TROSY spectra were recorded after addition of 0-20 mM albizziin (black to blue, see color legend). The signal of hA97 is marked by an ellipse.*

*(**D**) Methyl-TROSY spectra of WT HisFH (HisF $^{2}H^{15}N$-labeled, HisH ^{2}H, ILMVA methyl-$^{1}H^{13}C$-labeled) in the presence of ProFAR before (black) and after (red) addition of albizziin. Albizziin binds to the complex, but the active conformation is not formed.*
*(**E**) Same spectra for the HisFH-hC84S mutant. Addition of albizziin leads to the formation of the active conformation. The boxed regions highlight the signals of hV8 and hV111 and correspond to the region shown in Fig. 3 in the main text.*

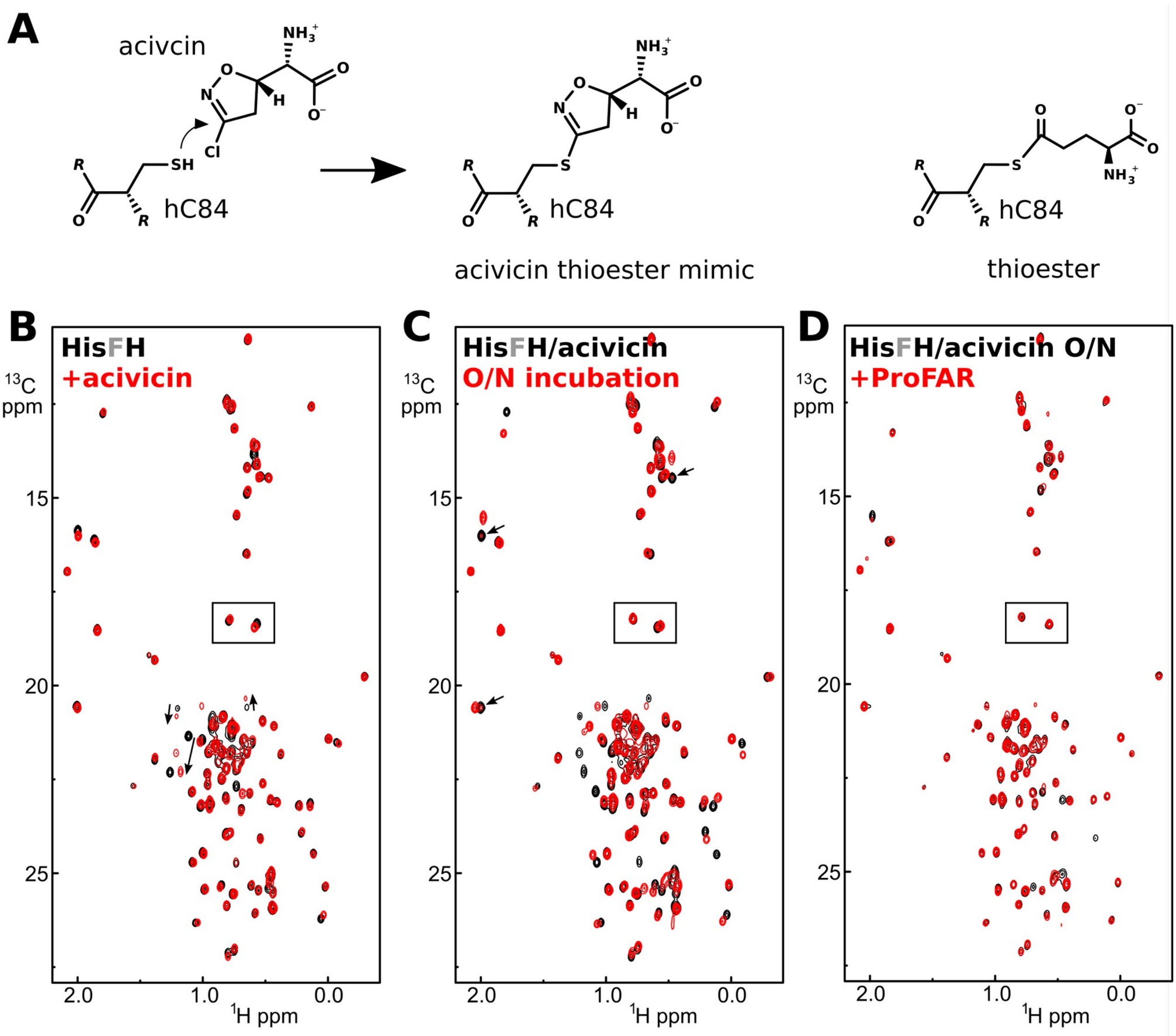

Supplementary Fig. 23 | The HisFH/acivicin/ProFAR complex does not form the active conformation.

*(**A**) Reaction of the covalent glutaminase inhibitor acivicin with the catalytic hC84 in HisH [3] leads to the formation of a thioester mimic. For comparison the natural thioester intermediate is shown (right).*

*(**B**) Methyl-TROSY spectra of WT HisFH (HisF $^{2}H^{15}N$-labeled, HisH ^{2}H, ILMVA methyl-$^{1}H^{13}C$-labeled) before (black) and after (red) addition of 5 mM acivicin. The CSPs show that acivicin binds similar to Gln (similar CSPs are indicated by arrows, compare to Suppl. Fig. 6), but more pronounced CSP after incubation overnight (O/N) indicate that the reaction with hC84 is much slower (see C). The boxed regions highlight the signals of hV8 and hV111 and corresponds to the region shown in Fig. 3 in the main text.*

*(**C**) Methyl-TROSY spectra of WT HisFH (HisF $^{2}H^{15}N$-labeled, HisH ^{2}H, ILMVA methyl-$^{1}H^{13}C$-labeled) in the presence of 5 mM acivicin before (black) and after (red) O/N incubation at 30 °C. The additional CSPs indicate that the thioester mimic was formed after O/N incubation. Very faint signals of the unreacted HisFH complex are still visible (indicated by arrows).*

*(**D**) Methyl-TROSY spectra of WT HisFH/acivicin complex (HisF $^{2}H^{15}N$-labeled, HisH ^{2}H, ILMVA methyl-$^{1}H^{13}C$-labeled), where the acivicin thioester mimic has been formed after incubation O/N*

before (black, same as red spectrum in C) and after addition of ProFAR (red). Weak CSP signal the binding of ProFAR, but the active conformation is not formed.

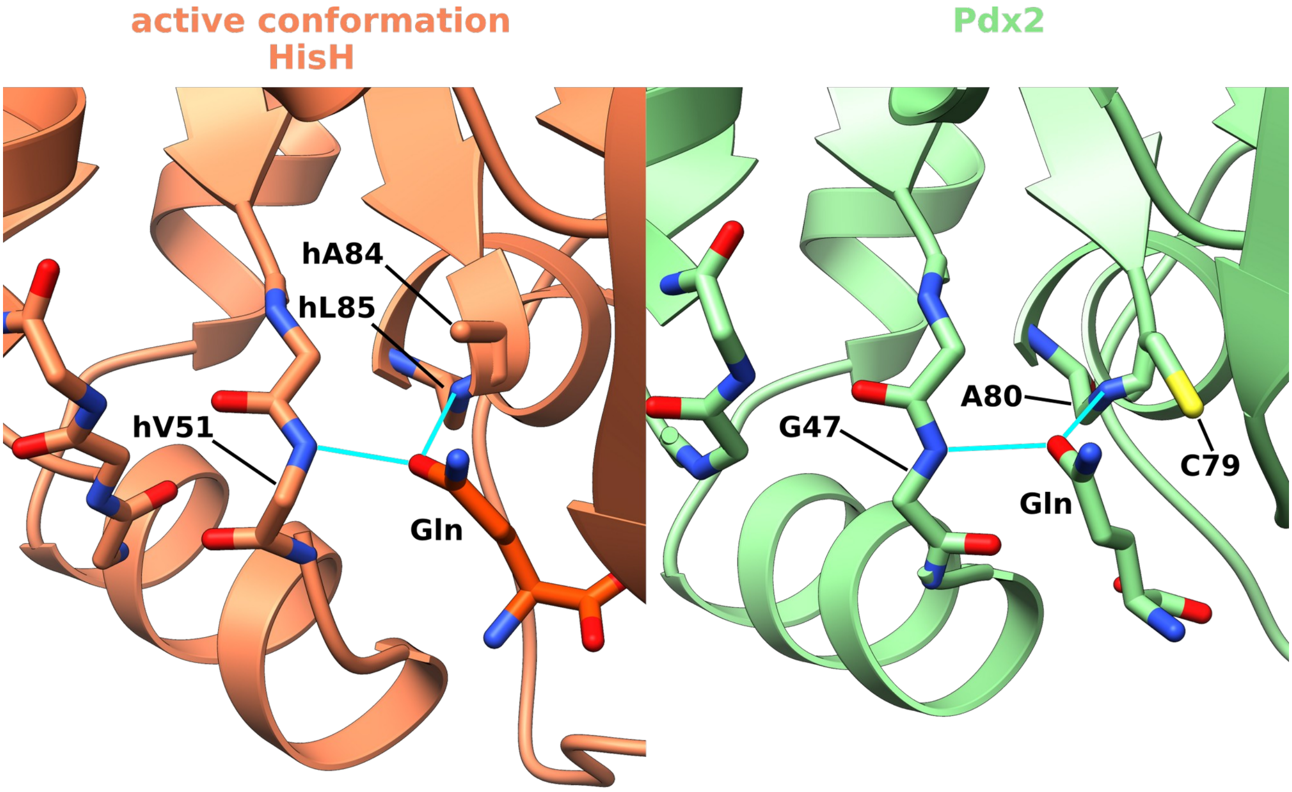

Supplementary Fig. 24 | The oxyanion holes of Pdx2 in the pyridoxal 5'-phosphate synthase complex and of HisH in the active conformation

Close-ups of the active sites of HisH (left, orange, active conformation of the HisFH complex; PDBID 7AC8, chains E/F) and Pdx2 (right, light green, PDB 2NV2, chain D). The other subunits of the complexes are omitted for clarity. In Pdx2 binding of Gln is sufficient for the formation of the oxyanion hole. The hydrogen bonds between the Gln amide oxygen and the oxyanion hole are shown in cyan. The active cite cysteine (mutated to hA84 in HisH) and the residues that form the oxyanion hole are labeled. Turnover of Gln by Pdx2 in the pyridoxal 5'-phosphate synthase complex is prevented by mutation of the catalytic histidine to asparagine (H170N, not shown).

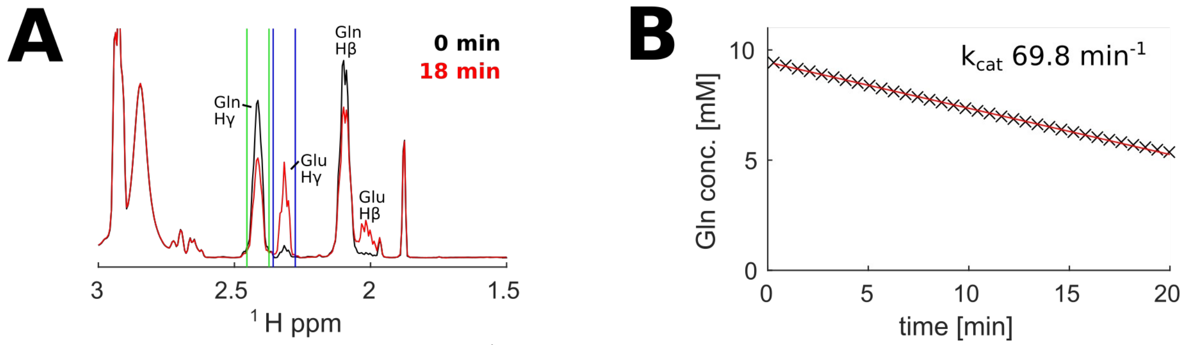

Supplementary Fig. 25 | Exemplary 1D-¹H NMR based glutaminase assay for WT HisFH in the presence of ProFAR.

*(**A**) Initial 1D-¹H spectrum (black) and spectrum recorded after 18 min. The regions used for the integration of the Gln and Glu Hγ signals are shown in green and blue, respectively. The following concentrations were used in the assay: Gln 10 mM, ProFAR 300 µM, WT HisFH 3 µM.*

*(**B**) The Gln concentration that is calculated from the peak integrals is plotted against incubation time (black). A straight line is fitted to the curve (red line). The k_{cat} determined form the fit is 69.8 min^{-1}. Source data are provided as a Source Data file.*

Supplementary Table 1 | Crystallographic data and refinement (PDBID: 7AC8)

Space group	$P3_2$
Cell dimensions (a, b, c (Å))	92.835 92.835 168.625
α, β, γ (°)	90.00 90.00 120.00
Resolution (Å)	2.06
R merge	0.08592 (1.052)
I/σ(I)	16.19 (2.21)
CC 1/2	0.999 (0.709)
Completeness (%)	99.99 (99.99)
Redundancy	10.3 (10.7)
**Refinement**	
Resolution (Å)	46.1 - 2.06 (2.134 - 2.06)
No. reflections	100493
R work / R free	0.1564 / 0.1861
**No. atoms**	11328
Protein	10656
ligands	79
Water	593
**B factors**	49.15
Protein	48.61
ligands	54.695
Water	58.093
**R.m.s. deviations**	
Bond lengths (Å)	0.008
Bond angles (°)	0.123
**Ramachandran statistics**	
Favored regions (%)	97.62
Allowed regions (%)	2.01
Outliers (%)	0.37

The highest resolution shell values are indicated in parenthesis.

Supplementary Table 2 | Influence of activators and HisFH mutants on glutaminase activity and the population of the active conformation.

HisF/HisH	activator	population active conformation [%]	glutaminase activity [min^{-1}]
WT/C84S	ProFAR	78 ± 3	63.0 ± 9.6
WT/C84S	ImGP	36 ± 2	14.1 ± 0.1
WT/C84S	AICAR	4 ± 2	1.0 ± 0.1
fD98E/C84S	ProFAR	100 ± 2	179.0 ± 2.8
fD98E/C84S	ImGP	100 ± 2	76.8 ± 7.6
fD98E/C84S	AICAR	81 ± 2	56.5 ± 3.5
fK19A/C84S	ProFAR	52 ± 1	20.4 ± 2.1
fF23A/C84S	ProFAR	7 ± 2	3.0 ± 0.4
fV48A/C84S	ProFAR	32 ± 1	6.8 ± 0.8
WT/C84A	ProFAR	89 ± 2	63.0 ± 9.6

Populations of the active conformation were determined in the HisH background in the presence of saturating concentrations of Gln and the given activator. Glutaminase activity was measured in the WT HisH background. Given are the average and standard deviation of the active state population (determined from n=8 methyl group signals; see method section for details) and the mean and standard deviation of the glutaminase activity (based on ≥ 16 timepoints and ≥ 2 independent experiments). Note: for fD98E with ProFAR and ImGP no inactive conformation is observable in the spectra and the error was estimated based on the noise level of the spectra. Source data are provided as a Source Data file.

Supplementary Table 3 | Plasmids used for protein expression.

Protein	Uniprot ID	Vector	Purification Tags	Mutations	#
HisF	Q9X0C6	pET-11	-	-	-
HisF	Q9X0C6	Modified pET-28	N-His_6-TEV	-	-
HisF	Q9X0C6	Modified pET-28	N-His_6-TEV	D98E	-
HisF	Q9X0C6	Modified pET-28	N-His_6-TEV	F23A	-
HisF	Q9X0C6	pET-11	-	K19A	-
HisF	Q9X0C6	pET-11	-	V48A	-
HisH	Q9X0C8	Modified pET-28	N-His_6-TEV	-	1861
HisH	Q9X0C8	Modified pET-28	N-His_6-TEV	C84A	-
HisH	Q9X0C8	Modified pET-28	N-His_6-TEV	C84S	-
HisH	Q9X0C8	Modified pET-28	N-His_6-TEV	I106L	2004
HisH	Q9X0C8	Modified pET-28	N-His_6-TEV	V111A	1998
HisH	Q9X0C8	Modified pET-28	N-His_6-TEV	V51A	1996
HisH	Q9X0C8	Modified pET-28	N-His_6-TEV	L153V	2001
HisH	Q9X0C8	Modified pET-28	N-His_6-TEV	V81I	1997
HisH	Q9X0C8	Modified pET-28	N-His_6-TEV	H178A	-
HisH	Q9X0C8	Modified pET-28	N-His_6-TEV	C84S, H178A	-
HisH	Q9X0C8	Modified pET-28	N-His_6-TEV	C84S, H53A	-

TEV: Tobacco Etch Virus protease site. The pET-28 was modified by insertion of an N-terminal, TEV cleavable hexahistidine tag (N-His_6-TEV).

Mutations have been introduced into the plasmids using the Quikchange (Aglient) or the Phusion (Finnzymes) site-directed mutagenesis approach.

Supplementary References:

1. Zalkin, H. & Smith, J. L. Enzymes Utilizing Glutamine as an Amide Donor. in *Advances in Enzymology - and Related Areas of Molecular Biology* (ed. Purich, D. L.) 87–144 (John Wiley & Sons, Inc., 2006). doi:10.1002/9780470123188.ch4.
2. Douangamath, A. *et al.* Structural evidence for ammonia tunneling across the (beta alpha)(8) barrel of the imidazole glycerol phosphate synthase bienzyme complex. *Structure* **10**, 185–193 (2002).
3. Chittur, S. V., Klem, T. J., Shafer, C. M. & Davisson, V. J. Mechanism for Acivicin Inactivation of Triad Glutamine Amidotransferases [†]. *Biochemistry* **40**, 876–887 (2001).
