# Supplementary material for: Molecular basis for the allosteric activation mechanism of the heterodimeric imidazole glycerol phosphate synthase complex
Source: Nat Commun. 2021 May 12;12:2748. doi: 10.1038/s41467-021-22968-6 (PMC8115485; doi:10.1038/s41467-021-22968-6)
Supplement: Supplementary file 2 — Peer Review File [file 41467_2021_22968_MOESM2_ESM.pdf]

REVIEWER COMMENTS

Reviewer #1 (Remarks to the Author):

In this manuscript Wurm et al. explore the allosteric coupling between the glutaminase subunit (HisH) in imidazole glycerol phosphate synthase (HisFH) with the subunit for the ultimate product formation (HisF). Binding of substrates to both subunits stimulates the hydrolysis of glutamine by a factor of approximately 4,500-fold. Previous experiments failed to identify any significant conformational changes to either subunit upon binding of a single ligand to either subunit. However, here they have now demonstrated for the first time that significant conformational changes are observed by NMR spectroscopy and X-ray crystallography when both subunits are occupied with ligands. The most significant changes appear to result in the formation of a functional oxyanion hole that is required for the hydrolysis of glutamine to glutamate and ammonia. The results have significant implications for a greater understanding of other glutamine-dependent enzymes that function to supply ammonia to another adjacent subunit. There are, however, a few issues that would make this manuscript better.

1. Most of the figures for the protein structures are too small to be of any use to the reader of this manuscript. These figures could be made larger in the SI.

2. It is curious that the authors have made no attempt to discuss the actual chemical mechanism for glutamine hydrolysis in this manuscript. In the enzymes from the amidotransferase family, the reaction proceeds through a thioester intermediate. Therefore, the overall reaction mechanism involves two different chemical events (formation of the thioester intermediate and hydrolysis of the thioester intermediate). One or both of these steps could be rate limiting in the absence or presence of the other substrate. I think that it would be important that the relative rates of these steps be measured so that a more complete picture of this allosteric mechanism be determined.

Reviewer #2 (Remarks to the Author):

The authors have studied the structure and structural dynamics of the bi-enzyme complex HisFH by means of solution NMR spectroscopy and X-ray crystallography. The enzyme complex consists of two enzymes, which catalyze a chemically coupled reaction as part of the histidine and purine biosynthesis. Intriguingly, the catalytic competence of both active sites is strictly coupled and dependent on the presence of both individual substrates. This coupling mechanism ensures that

ammonia liberation and its diffusive transfer between both components will only happen if the acceptor substrate at the second active site has been arrived. The structural basis of the 25 A long-range coupling has remained elusive and as such the molecular basis of allostery.

The authors now report on the first structural insights of the bi-enzyme complex that is found to be 'catalytically locked in'. Most notably, long-range coupling estabishes formation of the oxyanion hole as an essential asset of the active site of HisH. The dynamic equiblibrium between the various conformational substates is further characterized by TROSY NMR. All in all, the current work is a nice piece of work that expands the current understanding of HisHF but also provides general insights into long-range signaling in proteins. There are some points that should nonetheless be considered.

1) Is it possible to define the coupled networks linking the HisF and HisH active sites? Which types of interactions and structural transitions are key? Beyond HisHF, how this integrates into the more general picture of allostery and cooperativity (compare e.g. Cui Q, Karplus M. Allostery and cooperativity revisited. Protein Sci. 2008 Aug;17(8):1295-307.)

2) The reviewer noted that the authors used a Cys-to-Ser variant for the NMR experiments and a Cyst-to-Ala variant for the crystallographic analyses. Is there a rationale for this?

3) Related to point 2, are the autors confident that the X-ray structure of the variant is a blueprint for the structural transitions and coupled equilibria of the wild-type?

4) In the crystal structure with both substrates present, 3 HisHF molecules are present in the asymmetric unit: two in an 'inactive conformation' and one in a presumed 'active' conformation. Are these different conformations resulting from packing of the molecules in the lattice? It would be helpful to rationalize this observation.

5.) Some figures, in particular the ones with structural superpositions are a bit crowded and could be improved.

Reviewer #3 (Remarks to the Author):

This paper explores the mechanism of allostery in the imidazole glycerol phosphate synthase enzyme complex HisFH. HisFH is a bi-enzyme complex in which the substrate of the first reaction (Gln) binds to HisF and the substrate of the second reaction (PrFAR) binds to HisH. Remarkably, the chemical product of HisF is transmitted through HisFH and becomes a second substrate for the HisH reaction. It was previously known that PrFAR binding allosterically activates catalysis of Gln a large distance away in the complex. However, questions remained about the mechanism, especially since previous crystal structures showed little/no change upon binding of ligands. Here, the authors use a combination of NMR, X-ray crystallography, and mutagenesis to explore the allosteric mechanism linking the HisF and HisH active sites. They find that binding of either substrate alone is insufficient

to elicit any large-scale conformational changes, but simultaneous binding of both triggers a previously unseen activated conformation that affects both active sites. In this conformation, several conformational changes occur at the HisF-HisH interface, and the catalytic oxyanion hole in HisH is now formed. Through a series of different NMR experiments, the authors establish that the protein transitions dynamically between the inactive and active conformations (although this analysis is complicated by differences between wildtype vs. active-site inactivating mutations in different experiments). They also show a difference in timescales between fast Gln binding and slower conformational change to achieve the active conformation. Finally they demonstrate a correlation between destabilization of the active conformation induced by mutations or ligands (essentially a proxy for the occupancy or population of the active state) and catalytic rate.

MAJOR COMMENTS:

This paper has many merits. The HisFH system is very cool -- the bi-enzyme allosteric mechanism is quite intriguing! It is also a central metabolic enzyme, so is presumably of interest for that reason. The results are not shocking or groundbreaking per se, as it makes sense that higher occupancy of the active conformation would lead to higher activity. But the paper does provide direct evidence for that idea in this system, and importantly reveals the detailed nature of that active conformation for the first time. The authors performed a wealth of experiments to generate a trove of new data on this system. They make good use of a variety of tools, predominantly NMR but also some enzymology and crystallography, as well as various mutations and combinations of ligands (substrates, substrate mimics with various properties, etc.) to paint an overall picture. The prose is well written and easy to follow, and the figures are generally well organized and well labeled (some minor suggestions are below).

Although the results flow smoothly and mostly paint a cohesive picture, the reader is left a bit disappointed in the poor correlation between results for WT and hC84S (the inactivating active-site mutation that was necessary for most experiments). For example, the authors spend much of the paper characterizing the active state seen in hC84S, but then for WT enzyme (Fig 3e top) that active state is not present. They do perform additional analysis to bypass this problem (Fig 3f). We have some (minor) issues with the assumptions associated with this analysis (see below). However, overall the finding that the WT activity correlates with the hC84S-based active-state population proxy measurements is reasonably convincing.

In chain A in the deposited PDB, the stretch from 18-28 is marked in the PDB Validation Report as a poor fit to electron density, and A_K19 is marked as having particularly bad geometry. While we don't expect all regions to be perfect, both of these at the same time suggests greater scrutiny is required. This is particularly important as it concerns the conformation of the fL1 loop, which this manuscript describes in detail, and presumably uses as the reference structure for the inactive conformation of fL1 in HisFH. In the absence of provided structure factors / density maps, we

request that the authors reexamine this region of the model. As we mention below for line 227, we would also like to see density maps of this region in Supp Figs. We are reasonably confident that the conclusions drawn from these structural data are valid. Pending the results of the authors' reexamination of this region, we recognize the possibility that addressing this point may require redeposition, and subsequent re-rendering of the figures.

Overall, we recommend publication after our concerns are addressed.

MINOR COMMENTS:

Line 111-112: "an HisFH" = typo (presumably)

Line 113-116: I don't understand -- you speak of allostery between HisF and HisH, then give evidence only of ligands in HisF affecting dynamics also in HisF.

Line 130: "glutaminse"

Line 154-156: Hard to see by switching back & forth between Supp Fig 6 & 7 just how "very similar" the two spectra are. I mean, they look similar, but it's hard to say if they are essentially identical or not. A series of overlays of WT vs. hC84S (for different ligand situations, for HisF alone, for HisH alone, etc.) could help make this more clear.

Relatedly, line 183-185: "This new conformation is tolerant to the hC84S mutation in HisH as virtually identical results are obtained in a hC84A background (Suppl. Fig. 10) as well as for WT HisFH in combination with the covalent inhibitor DON (a thioester mimic; Suppl. Fig. 11)." Supp Fig. 10 has overlays for hC84A vs. hC84S -- I do not know why a similar overlay is not shown for wt vs. the mutants. I see that Supp Fig 11 features a truly WT spectrum including a molecule that induces a thioester mimic -- but it is unclear why true WT alone was not studied here, and why this spectrum was not overlaid with the mutant spectra as in Supp Fig 10.

Line 176: "These CSP are" - typo

Line 226: "The crystals were refined to a resolution of 2.06 Å" - imprecise language. Crystals are not refined in the sense that I think you mean. I think you mean the crystallographic models were refined. Also, not clear why it is plural, since you report only one structure…

Line 227: Show us the density for both conformations in a Supp Fig, preferably using omit maps! We as reviewers cannot inspect it ourselves since we were not provided with structure factors (although we were given PDB coordinates at least).

Line 230 + Fig 2 caption: Please note here which of the two inactive conformation complexes you are comparing against the active conformation in chains E/F? Which complex is used for making the figures? Is it chains A/B? Or C/D?

Fig 2(a): “4: Formation of the oxianion hole”: typo oxyanion

Line 231 vs. 235: You say one of the other chains has electron density for ProFAR with little structural effect, but that in one of chains E/F there is “clear” electron density and a large structural effect. This language subtly implies that the ProFAR density is stronger in chains E/F than in the other chains, perhaps corresponding to higher binding occupancy. Is that true?

Line 282: Should say “crystal structure”, not just “structure”

Line 297-298: “HisF in the inactive conformation bound to ProFAR and Gln” - But HisF doesn’t bind Gln… ?

Line 328: What is the k_on?

Line 343: “is not altered” -- those Kd’s are not identical within the error you report. But they are of course very similar.

Line 361-362: Can you be sure this is true for PrFAR though? It’s a reasonable question since you say that, although ImGP also can induce the active conf in the presence of Gln, it is a weaker activator (line 368). Perhaps you cannot say due to limitations of this experiment, but please comment at least.

Fig. 3d caption says “hC84S background”, but panel labels in Fig 3d itself say “wt”. Should we assume “wt” = “hC84S” i.e. not totally wildtype? This is a bit confusing since e.g. Fig. 3e also says “wt”, but in that case I think it is truly wildtype (not hC84S)... The reader should not have to reference the caption to discern that “wt” means different things in adjacent panels of the same figure.

Fig. 3 labeling: in d, text at top apparently = the black part of the plot. But in e, text at top = just a label for that panel. Inconsistent = confusing.

Line 379: Do you mean kcat?

Line 439-444: “The prerequisite for this approach is that HisF mutations and weaker activating ligands lead to a similar destabilization of the active conformation in the WT complex and in the hC84S mutant. This assumption is reasonable as the HisF active site and the HisF mutations are remote from the hC84S mutation. In agreement with that, we observe that the HisF NMR spectra are not perturbed by the HisH hC84S mutation.” What about epistasis? Perhaps the hC84S mutation alone has little effect on HisF but the combination of that with other perturbations has different/more effects. It is not clear to me that the HisF mutations and ligands must interact identically with WT vs. hC84S just because they are far away from each other -- after all, the point of this paper is that this is an allosteric complex! Of course, in the final analysis, Fig 3f seems to argue against this (?).

Line 657: Provide (approximate) crystal dimensions, and the (approximate) amount of the time it took for these crystals to grow to these dimensions.

Overall, the structure figures are good, not great. They could be improved by a more aggressive use of “fog” to obscure unnecessary, distracting elements from the background, and draw the reader’s attention to where it needs to be.

Supp Table 1: Cell dimensions are missing the 3 angles.

We review non-anonymously,

Daniel Keedy

Blake Riley

We thank the reviewers for their compliments on our work and for their constructive remarks. We have now adapted our manuscript accordingly. Below, we include a point-by-point response to the issues raised.

Reviewer #1

In this manuscript Wurm et al. explore the allosteric coupling between the glutaminase subunit (HisH) in imidazole glycerol phosphate synthase (HisFH) with the subunit for the ultimate product formation (HisF). Binding of substrates to both subunits stimulates the hydrolysis of glutamine by a factor of approximately 4,500-fold. Previous experiments failed to identify any significant conformational changes to either subunit upon binding of a single ligand to either subunit. However, here they have now demonstrated for the first time that significant conformational changes are observed by NMR spectroscopy and X-ray crystallography when both subunits are occupied with ligands. The most significant changes appear to result in the formation of a functional oxyanion hole that is required for the hydrolysis of glutamine to glutamate and ammonia. The results have significant implications for a greater understanding of other glutamine-dependent enzymes that function to supply ammonia to another adjacent subunit. There are, however, a few issues that would make this manuscript better.

1. Most of the figures for the protein structures are too small to be of any use to the reader of this manuscript. These figures could be made larger in the SI.
We thank reviewer 1 and also the other reviewers for commenting on this. We have changed the figures such that they display the structures significantly better.

2. It is curious that the authors have made no attempt to discuss the actual chemical mechanism for glutamine hydrolysis in this manuscript. In the enzymes from the amidotransferase family, the reaction proceeds through a thioester intermediate. Therefore, the overall reaction mechanism involves two different chemical events (formation of the thioester intermediate and hydrolysis of the thioester intermediate). One or both of these steps could be rate limiting in the absence or presence of the other substrate. I think that it would be important that the relative rates of these steps be measured so that a more complete picture of this allosteric mechanism be determined.
The reviewer raises a very important point, namely that the reaction takes place in two chemical steps. In **Suppl. Fig. S1**, we show these steps. In the reaction cycle the thio-ester formation results in the formation of the “thioester intermediate” and the hydrolysis of this intermediate results in the formation of glutamate. One of these steps (thioester formation or thioester hydrolysis) must be the rate limiting step during the chemical reaction. In our manuscript, we show that the oxyanion hole is formed in the active state of the enzyme. The oxyanion hole is crucial for both catalytic steps (thioester formation and thioester hydrolysis) and its formation is thus expected to enhance both steps significantly.
In the methyl TROSY NMR spectra of HisH we can directly determine if the active state has been formed or not. In the WT background we can mimic the thio-ester state using DON, that covalently binds to the active site cystein. In that “thio-ester mimic” situation we observe that HisH is mostly in the active state, where the oxyanion hole is fully formed (**Suppl. Fig. 12**). In the WT background in the presence of the natural substrate Gln (under multiple turnover; **Fig. 3e**), we don’t observe the active state of HisH. This implies that the majority of the protein is in the ground conformation under multiple turnover conditions. That observation implies that thio-ester hydrolysis is faster than thio-ester formation. We now include these considerations in the discussion part of the paper (page 22; lines 929-637).

Reviewer #2
The authors have studied the structure and structural dynamics of the bi-enzyme complex HisFH by means of solution NMR spectroscopy and X-ray crystallography. The enzyme complex consists of

two enzymes, which catalyze a chemically coupled reaction as part of the histidine and purine biosynthesis. Intriguingly, the catalytic competence of both active sites is strictly coupled and dependent on the presence of both individual substrates. This coupling mechanism ensures that ammonia liberation and its diffusive transfer between both components will only happen if the acceptor substrate at the second active site has been arrived. The structural basis of the 25 A long-range coupling has remained elusive and as such the molecular basis of allostery.
The authors now report on the first structural insights of the bi-enzyme complex that is found to be 'catalytically locked in'. Most notably, long-range coupling estabishes formation of the oxyanion hole as an essential asset of the active site of HisH. The dynamic equiblibrium between the various conformational substates is further characterized by TROSY NMR. All in all, the current work is a nice piece of work that expands the current understanding of HisHF but also provides general insights into long-range signaling in proteins. There are some points that should nonetheless be considered.
1) Is it possible to define the coupled networks linking the HisF and HisH active sites? Which types of interactions and structural transitions are key?
In **Fig. 2**, we show the structural changes that take place. We divide those into 4 different groups, as displayed in **Fig. 2c-e**. Clearly, we cannot unambiguously define the exact order of events. Nevertheless, we describe the most likely coupling of the events in the main text (as of line 239 in the old manuscript, or line 267 of the revised manuscript). In brief: first the HisF ligand binds to HisF which results in the closing of loop 1 in HisF (**Fig. 2c**). Secondly, the HisF hydrophobic core is rearranged. Thirdly, the orientation between HisF and HisH changes (**Fig. 2e**). Finally, the oxyanion hole in HisH is formed in the HisH active site when the glutamine substrate is present. Current research in our lab tries to identify the aminoacids that are central in this signal transmission process. To that end, we combine a mutational analysis with activity assays and NMR spectroscopy. These experiments go well beyond the scope of the current manuscript but are of course of high interest to us.

Beyond HisHF, how this integrates into the more general picture of allostery and cooperativity (compare e.g. Cui Q, Karplus M. Allostery and cooperativity revisited. Protein Sci. 2008 Aug;17(8):1295-307.)
We appreciate this remark of the reviewer. In the light of the excellent review by Cui and Karplus, we can state that the allosteric signaling in HisFH occurs through a shift in populations (the “new view”). The structural differences between the two states include changes in the tertiary (shifts of helices) and quaternary structure (HisF-HisH orientations) of the complex. We now refer to the review by Cui and Karplus in the manuscript.

2) The reviewer noted that the authors used a Cys-to-Ser variant for the NMR experiments and a Cyst-to-Ala variant for the crystallographic analyses. Is there a rationale for this?
For our crystallographic studies we tried both the hC84S mutant as well as the hC84A mutant. In the hC84S background the crystals dissolved upon soaking with the ProFAR ligand. For the hC84A mutant this did not happen. We now mention this in the methods section of the manuscript (line 771).

3) Related to point 2, are the autors confident that the X-ray structure of the variant is a blueprint for the structural transitions and coupled equilibria of the wild-type?
Yes, we are 100% confident that the hC84A as well as the hC84S mutants are representative of the WT enzyme as the NMR spectra (that are extremely sensitive to structural changes) of the active state that is formed by the WT enzyme in complex with DON and ProFAR is very similar to the NMR spectra of the hC84A and hC84S forms of the complex in the presence of glutamine. The structure of the active state of the enzyme is thus not influenced by the point mutation in the active site of HisH.

4) In the crystal structure with both substrates present, 3 HisHF molecules are present in the asymmetric unit: two in an 'inactive conformation' and one in a presumed 'active' conformation. Are these different conformations resulting from packing of the molecules in the lattice? It would be helpful to rationalize this observation.
In our HisFH/ Gln crystals all three complexes are in the inactive conformation (unpublished data). We then soaked these crystals with ProFAR to induced the active conformation. In general, it is hard to rationalize why structural changes in crystals do or don't take place upon soaking of ligands. Nevertheless in our case, the packing in the crystal of the HisFH complex shows that the 3 HisFH complexes are involved crystal packing to different degrees. The complex that changed the conformation in the crystal (from ground to active, chains E/F) is the complex that is least involved in packing interactions. This could rationalize why only this complex is observed in the active conformation. We have included a new supplementary figure (**Suppl. Fig. 13**) to visualize the crystal contacts.

5.) Some figures, in particular the ones with structural superpositions are a bit crowded and could be improved.
We agree with the reviewer (see also reviewer 1) and have improved our figures by making use of extensive depth cueing.

Reviewer #3
This paper explores the mechanism of allostery in the imidazole glycerol phosphate synthase enzyme complex HisFH. HisFH is a bi-enzyme complex in which the substrate of the first reaction (Gln) binds to HisF and the substrate of the second reaction (PrFAR) binds to HisH. Remarkably, the chemical product of HisF is transmitted through HisFH and becomes a second substrate for the HisH reaction. It was previously known that PrFAR binding allosterically activates catalysis of Gln a large distance away in the complex. However, questions remained about the mechanism, especially since previous crystal structures showed little/no change upon binding of ligands. Here, the authors use a combination of NMR, X-ray crystallography, and mutagenesis to explore the allosteric mechanism linking the HisF and HisH active sites. They find that binding of either substrate alone is insufficient to elicit any large-scale conformational changes, but simultaneous binding of both triggers a previously unseen activated conformation that affects both active sites. In this conformation, several conformational changes occur at the HisF-HisH interface, and the catalytic oxyanion hole in HisH is now formed. Through a series of different NMR experiments, the authors establish that the protein transitions dynamically between the inactive and active conformations (although this analysis is complicated by differences between wildtype vs. active-site inactivating mutations in different experiments). They also show a difference in timescales between fast Gln binding and slower conformational change to achieve the active conformation. Finally they demonstrate a correlation between destabilization of the active conformation induced by mutations or ligands (essentially a proxy for the occupancy or population of the active state) and catalytic rate.

MAJOR COMMENTS:

This paper has many merits. The HisFH system is very cool -- the bi-enzyme allosteric mechanism is quite intriguing! It is also a central metabolic enzyme, so is presumably of interest for that reason. The results are not shocking or groundbreaking per se, as it makes sense that higher occupancy of the active conformation would lead to higher activity. But the paper does provide direct evidence for that idea in this system, and importantly reveals the detailed nature of that active conformation for the first time. The authors performed a wealth of experiments to generate a trove of new data on this system. They make good use of a variety of tools, predominantly NMR but also some enzymology and crystallography, as well as various mutations and combinations of ligands (substrates, substrate mimics with various properties, etc.) to paint an overall picture. The

prose is well written and easy to follow, and the figures are generally well organized and well labeled (some minor suggestions are below).

Although the results flow smoothly and mostly paint a cohesive picture, the reader is left a bit disappointed in the poor correlation between results for WT and hC84S (the inactivating active-site mutation that was necessary for most experiments). For example, the authors spend much of the paper characterizing the active state seen in hC84S, but then for WT enzyme (Fig 3e top) that active state is not present. They do perform additional analysis to bypass this problem (Fig 3f). We have some (minor) issues with the assumptions associated with this analysis (see below). However, overall the finding that the WT activity correlates with the hC84S-based active-state population proxy measurements is reasonably convincing.
The fact that the active conformation of the enzyme is not observable in our NMR experiments is a direct consequence of the low population of this active state in the WT background. The hC84S mutant form of the enzyme shifted the populations towards the active state and thus allowed us to structurally study the active conformation in detail. We fully agree with the reviewers that it would be preferable to perform all experiments in a WT background, however, this is unfortunately not possible. In that light, our approach is the only option we have to gain insights into the fascinating allosteric mechanism in HisFH.

In chain A in the deposited PDB, the stretch from 18-28 is marked in the PDB Validation Report as a poor fit to electron density, and A_K19 is marked as having particularly bad geometry. While we don't expect all regions to be perfect, both of these at the same time suggests greater scrutiny is required. This is particularly important as it concerns the conformation of the fL1 loop, which this manuscript describes in detail, and presumably uses as the reference structure for the inactive conformation of fL1 in HisFH. In the absence of provided structure factors / density maps, we request that the authors reexamine this region of the model. As we mention below for line 227, we would also like to see density maps of this region in Supp Figs. We are reasonably confident that the conclusions drawn from these structural data are valid. Pending the results of the authors' reexamination of this region, we recognize the possibility that addressing this point may require redeposition, and subsequent re-rendering of the figures.
We thank the reviewers for pointing this out. For the figures we mostly have used chains C/D for the ground state conformation and chains E/F for the active state conformation. The poorer quality of the model in the region of loop1 in chain A thus does not influence our conclusions and figures.
The HisF active site is occupied with ProFAR in the chain A. This likely slightly destabilizes the open conformation of loop 1. This then results in the poor electron density and consequently in increased B-factors. We show this in a new supplementary figure (**Supp. Fig. 14**).
We also re-refined our structure in this region. However, we found that this did not result in changes in the model or the quality thereof. We therefor decided to stick to the model that we initially deposited. For completeness, we have now uploaded the mtz files for the reviewers.

Overall, we recommend publication after our concerns are addressed.

MINOR COMMENTS:
Line 111-112: "an HisFH" = typo (presumably)
We corrected this to "a HisFH"

Line 113-116: I don't understand -- you speak of allostery between HisF and HisH, then give evidence only of ligands in HisF affecting dynamics also in HisF.
We agree that this was phrased sub-optimally. We now write: "NMR measurements show that binding of ligands to HisF results in increased dynamics that extend from the HisF active site to the HisF-HisH interface and it has been proposed that these dynamics are part of the allosteric coupling between the two enzymes."

Line 130: “glutaminse”
We corrected this into “glutaminase”

Line 154-156: Hard to see by switching back & forth between Supp Fig 6 & 7 just how “very similar” the two spectra are. I mean, they look similar, but it’s hard to say if they are essentially identical or not. A series of overlays of WT vs. hC84S (for different ligand situations, for HisF alone, for HisH alone, etc.) could help make this more clear.
We have now prepared a novel supplementary figure (**Suppl. Fig. 8**) to show this unambiguously. In detail, we display overlays of the WT HisFH complex (all in black) with the hC84S HisFH complex (all in red). The overlays are shown for HisF (H-N spectra, top row), as well as for HisH (methyl TROSY spectra, bottom row) for the apo complex (left column), the complex in the presence of Gln (middle column) as well as for the complex in the presence of ProFAR (right column). Based on that, it is clear that the hC84S mutation has only a very limited effect on the NMR spectra (and thus structure) of the HisFH complex.

Relatedly, line 183-185: “This new conformation is tolerant to the hC84S mutation in HisH as virtually identical results are obtained in a hC84A background (Suppl. Fig. 10) as well as for WT HisFH in combination with the covalent inhibitor DON (a thioester mimic; Suppl. Fig. 11).” Supp Fig. 10 has overlays for hC84A vs. hC84S -- I do not know why a similar overlay is not shown for wt vs. the mutants. I see that Supp Fig 11 features a truly WT spectrum including a molecule that induces a thioester mimic -- but it is unclear why true WT alone was not studied here, and why this spectrum was not overlaid with the mutant spectra as in Supp Fig 10.
We now show the overlay of the WT and the hC84S complex in a novel supplementary figure (**Suppl. Fig. 8**; see response to remark above). In **Suppl. Fig 12**, we overlay the WT complex in the absence and presence of DON, i.e. in the absence of the hC84S or hC84A mutation.

Line 176: “These CSP are” – typo
We corrected this.

Line 226: “The crystals were refined to a resolution of 2.06 Å” - imprecise language. Crystals are not refined in the sense that I think you mean. I think you mean the crystallographic models were refined. Also, not clear why it is plural, since you report only one structure…
We agree that this is incorrectly phrased and have re-phrased this. We thank the reviewers for pointing us to this inaccuracy.

Line 227: Show us the density for both conformations in a Supp Fig, preferably using omit maps! We as reviewers cannot inspect it ourselves since we were not provided with structure factors (although we were given PDB coordinates at least).
We have prepared a supplementary figure (**Suppl Fig 14**) that shows the composite electron density for loop 1 and for the ligands ProFAR and glutamine (Composite omit 2Fo - Fc electron density map, contoured at 1.0 σ).
Panels A, B and C show the active conformation in chains E and F. Loop 1, ProFAR and glutamine are well defined in the electron density.
Panels D, E and F show the inactive conformation in chains A and B. Loop 1 less well defined, the ligands are well defined.
Panels G and H show the inactive conformation in chains C and D. Loop 1 and glutamine are well defined. There is no electron density for ProFAR in this complex.

Line 230 + Fig 2 caption: Please note here which of the two inactive conformation complexes you are comparing against the active conformation in chains E/F? Which complex is used for making the figures? Is it chains A/B? Or C/D?

For in inactive conformation mostly images have been prepared using chains C/D. In all figure legends the chains that are used are now clearly indicated.

Fig 2(a): "4: Formation of the oxianion hole": typo oxyanion
We corrected this.

Line 231 vs. 235: You say one of the other chains has electron density for ProFAR with little structural effect, but that in one of chains E/F there is "clear" electron density and a large structural effect. This language subtly implies that the ProFAR density is stronger in chains E/F than in the other chains, perhaps corresponding to higher binding occupancy. Is that true?
The electron density for ProFAR in the C/D and E/F complexes is similar. We removed the word "clear" for the description of complex E/F to prevent confusion.

Line 282: Should say "crystal structure", not just "structure"
We changed "structure" to "crystal structure"

Line 297-298: "HisF in the inactive conformation bound to ProFAR and Gln" - But HisF doesn't bind Gln… ?
We thank the reviewers for pointing out this inaccuracy. The figure legend now reads "Closeup of the HisF active site: Comparison between the inactive (left) and the active conformation (right)."

Line 328: What is the k_on?
Based on the kD (1.3 mM for WT HisH and 1.7 mM for HisH hC84S) and k_off (1600 for WT HisH and 2100 for HisH hC84S), the k_on (=k_off/kD) can be determined to be 1.2 $*10^6$ $M^{-1}s^{-1}$ for the WT as well as for the hC84S forms of HisH. This is now mentioned in the text.

Line 343: "is not altered" -- those Kd's are not identical within the error you report. But they are of course very similar.
We agree that those values are very similar. We changed "is not altered" to "is not significantly changed".

Line 361-362: Can you be sure this is true for PrFAR though? It's a reasonable question since you say that, although ImGP also can induce the active conf in the presence of Gln, it is a weaker activator (line 368). Perhaps you cannot say due to limitations of this experiment, but please comment at least.
The reviewer is absolutely right, we have not been able to accurately determine these rates in the presence of PrFAR (that is by far too unstable to perform any NMR measurement; **Suppl. Fig. 4**) or in the presence of ProFAR (that is not compatible with the lengthy NMR measurements). We were, however, able to record a single time-point of a longitudinal exchange experiment in the presence of ProFAR. In this case, the intensities of the cross peaks (that directly report on the exchange rates) are similar to the cross peaks that we observe in the presence of ImGP (updated **Suppl. Fig. 18**). This clearly shows that the exchange rates in the presence of ImGP and in the presence of ProFAR are similar. Based on that, the ground-active state conversions are faster than the turnover rates also in the presence of ProFAR. We now state this in the manuscript (lines 421-424).

Fig. 3d caption says "hC84S background", but panel labels in Fig 3d itself say "wt". Should we assume "wt" = "hC84S" i.e. not totally wildtype? This is a bit confusing since e.g. Fig. 3e also says "wt", but in that case I think it is truly wildtype (not hC84S)... The reader should not have to reference the caption to discern that "wt" means different things in adjacent panels of the same figure.
The reviewer understood things correctly, and we agree that the labeling was confusing. We changed the labels in the items that are displayed in the figure.

Fig. 3 labeling: in d, text at top apparently = the black part of the plot. But in e, text at top = just a label for that panel. Inconsistent = confusing.
Again, we understand the confusion and have changed the labels accordingly.

Line 379: Do you mean kcat?
Indeed, this we now clearly state.

Line 439-444: "The prerequisite for this approach is that HisF mutations and weaker activating ligands lead to a similar destabilization of the active conformation in the WT complex and in the hC84S mutant. This assumption is reasonable as the HisF active site and the HisF mutations are remote from the hC84S mutation. In agreement with that, we observe that the HisF NMR spectra are not perturbed by the HisH hC84S mutation." What about epistasis? Perhaps the hC84S mutation alone has little effect on HisF but the combination of that with other perturbations has different/more effects. It is not clear to me that the HisF mutations and ligands must interact identically with WT vs. hC84S just because they are far away from each other -- after all, the point of this paper is that this is an allosteric complex! Of course, in the final analysis, Fig 3f seems to argue against this (?).
In brief, our finding is that the HisFH complex can adopt two structurally different states (ground and active). Mutations in HisF or in HisH are able to shift the populations of those two states. What we argue here is that the (ΔΔG) effects of mutations that shift the populations are additive.
Of course the reviewer is correct, things could be much more complicated than we assume. However, based on the Occam's razors principle, we tried to first assume the least complicated explanation. The findings that we extract based on these assumptions show there was indeed no need to invoke additional parameters in the analysis.

Line 657: Provide (approximate) crystal dimensions, and the (approximate) amount of the time it took for these crystals to grow to these dimensions.
We have now included this information in the method section of the manuscript (lines 770-771): "Crystals appeared after 2 days and reached the final size (0.3 x 0.4 x 0.1 mm) after three to four additional days. "

Overall, the structure figures are good, not great. They could be improved by a more aggressive use of "fog" to obscure unnecessary, distracting elements from the background, and draw the reader's attention to where it needs to be.
We appreciate this suggestion and have updated the figures accordingly. In our opinion things are indeed much clearer now. See also the remarks of the other reviewers.

Supp Table 1: Cell dimensions are missing the 3 angles.
The angles α, β and γ are defined by the space group $P3_2$ to be 90.00, 90.00 and 120.00 degrees respectively. We have now included this missing information in **Suppl. Table 1**.

We review non-anonymously,
Daniel Keedy
Blake Riley

Final remarks
In addition to the edits mentioned above, we have corrected a number of type-o's that we found after submission. All changes to the originally submitted manuscript are incorporated using the "track-changes" option. Note that the line-numbering has changed slightly due to the conversion of our manuscript from Libreoffice Writer format into Microsoft Word (that is requested by the publisher).

REVIEWER COMMENTS

Reviewer #1 (Remarks to the Author):

The revised manuscript has satisfied my concerns.

Reviewer #2 (Remarks to the Author):

The authors have constructively addressed all issues raised by the reviewers such that acceptance would be supported by this reviewer.

Reviewer #3 (Remarks to the Author):

We thank the authors for responding to our comments. After perusing their additions and changes to figures, captions, and portions of the text, we feel they are generally appropriate.

We also thank the authors for providing the model and electron density maps for the crystal structure (PDB ID 7AC8). Having now examined them in some detail, we have three remaining concerns about the model, with varying degrees of implications for the conclusions of the paper.

ProFAR in chain C

First, and most importantly, we have concerns over the presence vs. absence of ProFAR in chain C. In the caption for Supp Fig 14 (and implied on lines 258-259, and 753), the authors state “There is no electron density for ProFAR in this complex”, referring to the chain C+D “inactive” complex. The model and maps that the authors provide are apparently in conflict with this statement: they have modelled selected fragments of ProFAR into the ProFAR binding site here: a PO4, a ribose phosphate, and a few waters.

We note the authors have labeled these terminal atoms as AMZ, i.e. AICAR, i.e. the product of the PrFAR reaction (as in Fig 1A), with atoms from the aminoimidazole ring deleted. This claim, present in the crystallographic model, must be discussed in the text of the manuscript. Is it plausible that this fragment is AICAR (i.e. is there a reaction mechanism by which ProFAR can be converted to AICAR?)? Do the authors have any other evidence to justify their claim of product being bound?

That said, if it is indeed AICAR, why have they not mentioned that the HisF in chain C is in a product-bound state, per their model? It is strange that they state "there is no [...] ProFAR in this complex" in Supp Fig 14 caption, but neglect to mention what would obviously be of interest to the reader: that a product is bound there instead! One piece of evidence arguing against it being AICAR is that it is unclear where the adjacent modeled PO4 would have come from in this case. As neither the protein buffer nor the crystallization buffer contain phosphates, the authors will need to explain the source of the modelled PO4.

With that in mind, are you sure the molecule in question is AICAR (with a PO4 nearby)? We note the possibility that it is actually ProFAR with both ends ordered (including the PO4 group), and the middle disordered. This alternative explanation is plausible given the chemical structure of ProFAR, with several DOFs in the middle. A Coot NCS ghost of the other inactive complex (chains A+B), which does have ProFAR modelled, aligns well with the authors' modelled fragments in chain C, and provides a suggestion for where ProFAR could be modelled. This may also clear up a -6.87σ difference map peak over the PO4 in chain C, as the PO4 may need to be repositioned to displace some currently modeled waters.

In either case, Supp Fig 14 should show the 2Fo-Fc density for the binding site of ProFAR along with Fo-Fc difference map density carved to some distance (e.g. 3 Å) around the ligand site. The figure should include panels for each of ProFAR vs. AICAR to show which fits best. The text "no electron density for ProFAR" should be adjusted accordingly.

Other experiments could potentially help reveal the identity of the mystery species in chains C+D. For example, the authors could dissolve ProFAR-soaked crystals, then conduct mass spectrometry to ascertain whether AICAR is present in the crystals, as opposed to only ProFAR. If AICAR is present, an additional MS experiment on the ProFAR solution that was used for crystal soaking could then reveal whether the reaction occurred enzymatically in the crystal vs. non-enzymatically in solution (presumably slowly either way).

We emphasize this aspect because of the potential that additional details from this structure could add nuance to the biological conclusions from this paper. Either explanation is potentially

biologically interesting. For example, the Introduction states that AICAR (not just ProFAR) can activate the complex. Thus, if the species in chain C is AICAR, then maybe chains C+D are activated, not inactivated as Supp Fig 14 says -- but perhaps the product (AICAR) just activates the complex in a different way than the substrate (ProFAR) does? By contrast, what if the species in chain C is ProFAR, but it is just bound in a novel, partially disordered conformation, with only the two ends ordered? Can something interesting be learned about HisFH allostery from the conformational coupling between differently ordered ProFAR states and differently ordered nearby loop fL1 states in the different complexes in the ASU?

Chain A, residues 18-28

Second, having examined the model + maps, we now feel that residues 18-28 in chain A which we previously indicated was an outlier in the PDB Validation Report is indeed misfit. For example, it is clear from the Fo-Fc map that His228 needs to switch rotamer, which would lead to a clash with the current position for Lys19; that combined with the poor density for Lys19 suggests that Lys19 is mismodeled. This region is adjacent to the ligand binding site, which makes the modeling issues unfortunate. However, we were unable to readily remodel this region ourselves (albeit after limited attempts), so we understand that the authors were unable to reach a perfect model. Still, when the authors re-refine for issue #1 above, they may wish to try refitting this region again as well.

Remaining difference map peaks

Third, there are some very bright peaks in the Fo-Fc difference map, suggesting that the authors still have some remaining modelling of accessory ligands, etc. to do. For example, in chain C, the amide N-H vector of residue GLU251 points towards a +14.75σ difference map peak. In chain A, this is a +9.67σ peak; in chain E, this is a +8.19σ peak. The presence of such a bright peak, present in all NCS copies in the unit cell, strongly suggests to us that this site is occupied by an unmodelled atom. The buffers used suggest that this could be either K, Na or Cl, and the neighboring presence of Arg249 suggests that it could be Cl-. However, the authors would know better. Is this a known ion-binding site in HisFH? This issue is more minor as it is unlikely to affect biological interpretations in this paper based on the fact that the site in question is quite distal from both of the main ligand binding sites. Still, when the authors re-refine for issue #1 above, they may wish to try refitting these areas as well.

As a final aside, we still feel that the effects of mutations and activating ligands in WT vs. hC84S backgrounds may not be truly identical, but the paper presents sufficient overall evidence that they are comparable enough to justify the treatment here in, for example, Fig. 3f.

Overall, we feel that additional revisions are required to address these points regarding the crystal structure.

We (still) review non-anonymously,

Daniel Keedy

Blake Riley

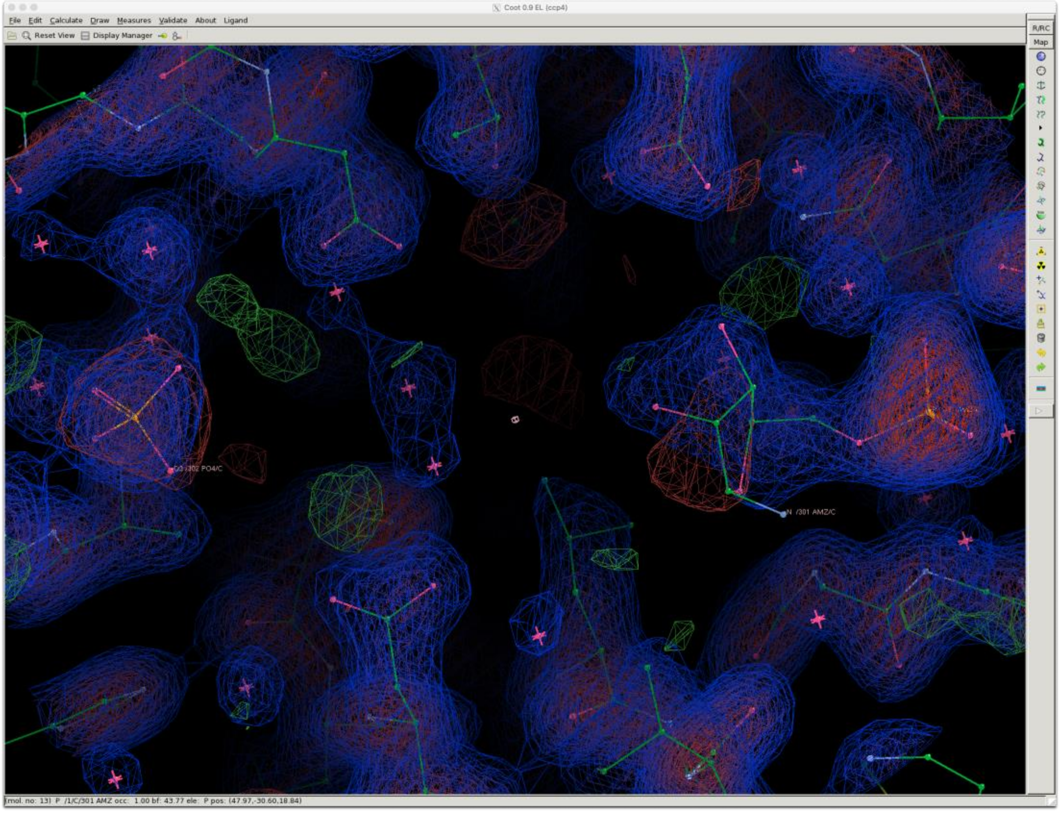

Figure 1: Authors' model, 2Fo-Fc map (as Coot multi-contour, outer contour 1σ) & Fo-Fc map (green mesh = +3σ, red mesh = -3σ). View is centred on the ProFAR binding site of chain C.

On the right of the image is a ribose phosphate fragment of ProFAR, labelled 301 AMZ.

On the left of the image is a phosphate (likely a fragment of ProFAR), labelled 302 PO4, enclosed by a negative difference mesh.

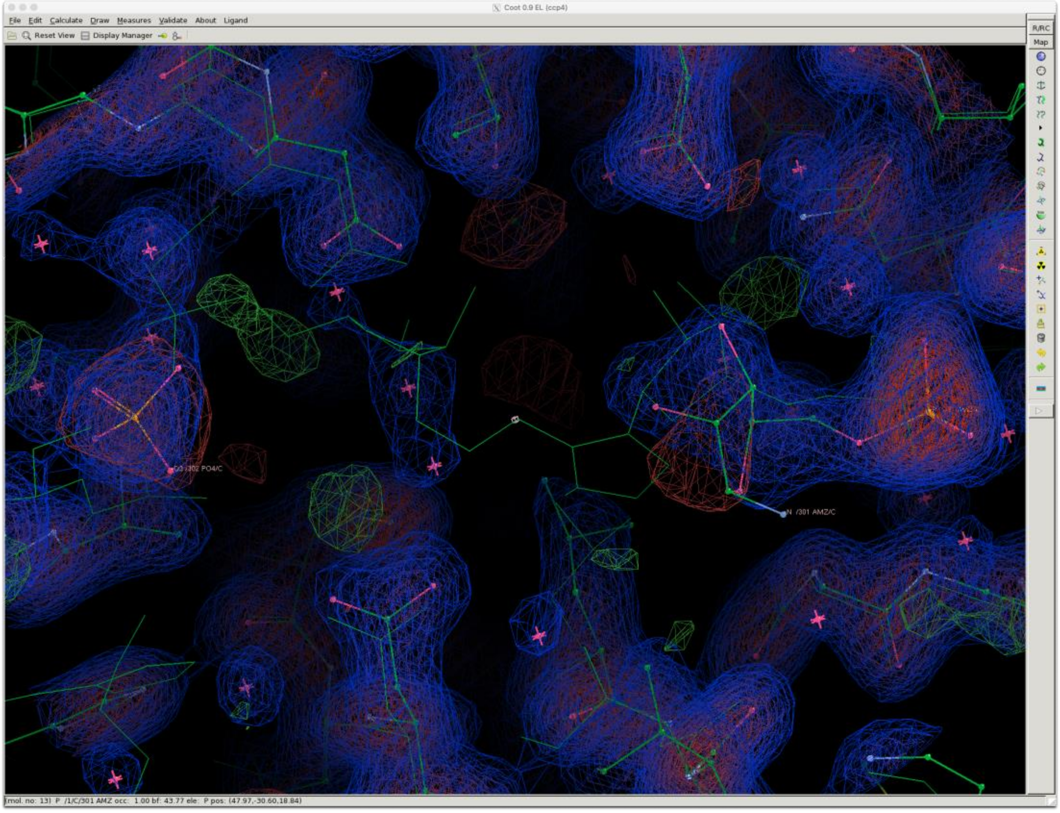

Figure 2: The exact same view, but with chain A aligned and overlayed (Coot NCS ghost). Chain A has ProFAR modelled, aligns well with the authors' modelled fragments in chain C, and provides a suggestion for where the ProFAR could be modelled in this density.

We thank the reviewers for their compliments on our work and for their constructive remarks. We have now adapted our manuscript accordingly. Below, we include a point-by-point response to the issues raised.

Reviewer #1 (Remarks to the Author):

The revised manuscript has satisfied my concerns.

Reviewer #2 (Remarks to the Author):

The authors have constructively addressed all issues raised by the reviewers such that acceptance would be supported by this reviewer.

We thank the reviewers for their time and efforts in improving our manuscript.

Reviewer #3 (Remarks to the Author, see also attached PDF):

We thank the authors for responding to our comments. After perusing their additions and changes to figures, captions, and portions of the text, we feel they are generally appropriate.

We thank the reviewers for these positive remarks.

We also thank the authors for providing the model and electron density maps for the crystal structure (PDB ID 7AC8). Having now examined them in some detail, we have three remaining concerns about the model, with varying degrees of implications for the conclusions of the paper.

We thank the reviewers for their careful and important remarks regarding the X-ray model. It is clear that the raised points are important and addressing those has improved the quality of our structure. At the same time, the conclusions the we drew from the structures are not effected by the adjustment the we made to the model.

ProFAR in chain C
First, and most importantly, we have concerns over the presence vs. absence of ProFAR in chain C. In the caption for Supp Fig 14 (and implied on lines 258-259, and 753), the authors state “There is no electron density for ProFAR in this complex”;, referring to the chain C+D “inactive”; complex. The model and maps that the authors provide are apparently in conflict with this statement: they have modelled selected fragments of ProFAR into the ProFAR binding site here: a PO4, a ribose phosphate, and a few waters.

We thank the reviewer for spotting the inconsistency between the coordinate files and the text of the manuscript. We have now carefully revised the density in the HisF active site in chain C. It is clear that the previously modeled in AICAR/ PROFAR required a correction. To that end, we removed the previously modeled ligand and have now replaced this with a phosphate and a solvent molecule. Details are presented in Supplementary Fig. 15B and are discussed in more detail below.

We note the authors have labeled these terminal atoms as AMZ, i.e. AICAR, i.e. the product of the PrFAR reaction (as in Fig 1A), with atoms from the aminoimidazole ring deleted. This claim, present in the crystallographic model, must be discussed in the text of the manuscript. Is it plausible that this fragment is AICAR (i.e. is there a reaction mechanism by which ProFAR can be converted to AICAR?)? Do the authors have any other evidence to justify their claim of product being bound?

As mentioned above, we have now removed the bound product/ substrate as the data did not allow for the accurate and complete modeling of AICAR or ProFAR. We have no evidence that chain C is bound to the AICAR product of the reaction.

That said, if it is indeed AICAR, why have they not mentioned that the HisF in chain C is in a product-bound state, per their model? It is strange that they state ";there is no [...] ProFAR in this complex"; in Supp Fig 14 caption, but neglect to mention what would obviously be of interest to the reader: that a product is bound there instead! One piece of evidence arguing against it being AICAR is that it is unclear where the adjacent modeled PO4 would have come from in this case. As neither the protein buffer nor the crystallization buffer contain phosphates, the authors will need to explain the source of the modeled PO4.

With that in mind, are you sure the molecule in question is AICAR (with a PO4 nearby)? We note the possibility that it is actually ProFAR with both ends ordered (including the PO4 group), and the middle disordered. This alternative explanation is plausible given the chemical structure of ProFAR, with several DOFs in the middle. A Coot NCS ghost of the other inactive complex (chains A+B), which does have ProFAR modelled, aligns well with the authors'; modelled fragments in chain C, and provides a suggestion for where ProFAR could be modelled. This may also clear up a -6.87σ ; difference map peak over the PO4 in chain C, as the PO4 may need to be repositioned to displace some currently modeled waters.

We have removed the AICAR from chain C. The active center of chain C contains residual density in two locations and at sites equivalent to the two terminal ProFAR phosphate positions in chains A and E. The presence of isolated phosphate ions can be dismissed, as no phosphate was added to the crystallization medium or during ligand soaking. The most plausible explanation for the density in the active site of HisF in chain C is thus the presence of ProFAR, however, with a low occupancy or with high mobility.

Inspired by the reviewers remarks, we decided for a conservative approach and modeled the stronger density as phosphate (chain C, PO4 # 301) and the weaker density as a water molecule (chain S, HOH # 932). We also tried to model the second supposed phosphate group as a phosphate ion, however, this generated significant negative difference density after refinement. Consequently, we decided for a more conservative approach and modeled this site as a water molecule. This is now clearly highlighted in Suppl. Fig. 15. In addition, we have asked the PDB to include a comment into the respective mmCIF file, to clarify that the annotation of these two entities present placeholders of our best guess. The Supplementary Figure S14 has been modified accordingly.

In the manuscript, it is now explicitly mentioned that the density in the HisF active site of chain C is likely a loosely bound ProFAR molecule (lines 237-240).

In either case, Supp Fig 14 should show the 2Fo-Fc density for the binding site of ProFAR along with Fo-Fc difference map density carved to some distance (e.g. 3 Å;) around the ligand site. The figure should include panels for each of ProFAR vs. AICAR to show which fits best. The text ";no electron density for ProFAR"; should be adjusted accordingly.

We have prepared an updated Suppl. Fig 15, that displays the 2Fo-Fc and Fo-Fc densities for all copies of the HisF active site.

Other experiments could potentially help reveal the identity of the mystery species in chains C+D. For example, the authors could dissolve ProFAR-soaked crystals, then conduct mass spectrometry to ascertain whether AICAR is present in the crystals, as opposed to only ProFAR. If AICAR is

present, an additional MS experiment on the ProFAR solution that was used for crystal soaking could then reveal whether the reaction occurred enzymatically in the crystal vs. non-enzymatically in solution (presumably slowly either way).

We thank the reviewers for these suggestions. These suggestions, however, go beyond the scope of the current manuscript. In addition, a quantitative mass spectrometry analysis is not feasible at present as we would have to freeze structural states after defined time points after start of the ligand soaks. We have no access to such an experimental setup at the moment. In addition, we have limited the interpretation of our structural data to those chains where the evidence for ProFAR binding is unambiguous (chains A/B and E/F).

We emphasize this aspect because of the potential that additional details from this structure could add nuance to the biological conclusions from this paper. Either explanation is potentially biologically interesting. For example, the Introduction states that AICAR (not just ProFAR) can activate the complex. Thus, if the species in chain C is AICAR, then maybe chains C+D are activated, not inactivated as Supp Fig 14 says -- but perhaps the product (AICAR) just activates the complex in a different way than the substrate (ProFAR) does? By contrast, what if the species in chain C is ProFAR, but it is just bound in a novel, partially disordered conformation, with only the two ends ordered? Can something interesting be learned about HisFH allostery from the conformational coupling between differently ordered ProFAR states and differently ordered nearby loop fL1 states in the different complexes in the ASU?

The reviewers are right, AICAR is able to activate the complex to a very limited degree (1.6 % of the activity of the ProFAR bound state). The chains C/D are, however, clearly in an inactive conformation: loop 1 is not docked onto the HisF active site, the orientation between HisF and HisH correspond to the inactive state and the oxyanion hole is not formed. As discussed above, the bound ligand is most likely a loosely bound ProFAR molecule. Although maybe tempting, it is not possible to correlate the "differently ordered ProFAR states" with allostery, as we have no direct experimental evidence on the mobility of ProFAR in the active site of HisF. Any conclusions that would be drawn based on the (poor) density around the active site of HisF in chain C and a molecular mechanism would be pure speculation. Future NMR studies that focus on ProFAR might be able to provide direct information.

Chain A, residues 18-28
Second, having examined the model + maps, we now feel that residues 18-28 in chain A which we previously indicated was an outlier in the PDB Validation Report is indeed misfit. For example, it is clear from the Fo-Fc map that His228 needs to switch rotamer, which would lead to a clash with the current position for Lys19; that combined with the poor density for Lys19 suggests that Lys19 is mismodeled. This region is adjacent to the ligand binding site, which makes the modeling issues unfortunate. However, we were unable to readily remodel this region ourselves (albeit after limited attempts), so we understand that the authors were unable to reach a perfect model. Still, when the authors re-refine for issue #1 above, they may wish to try refitting this region again as well.

We revised the coordinate file, by removing side chains of the residues listed below where there is no significant density. With respect to the loop covering residues 18-28 in chain A, we remodeled this segment and improved its conformation, using the equivalent loop from chain C with better resolved density for guidance.

The following residues were removed due to a lack of significant density:

Chain A: L253
Chain B: R201

Chain C: L253
Chain D: R200, R201
Chain E: G252, L253
Chain F: S199, R200, R201

The side chains of the following residue were removed due to a lack of significant density.
Chain A: E24, N25, R27, H228, R230
Chain B: R200
Chain C: E24

Remaining difference map peaks
Third, there are some very bright peaks in the Fo-Fc difference map, suggesting that the authors still have some remaining modelling of accessory ligands, etc. to do. For example, in chain C, the amide N-H vector of residue GLU251 points towards a +14.75σ difference map peak. In chain A, this is a +9.67σ peak; in chain E, this is a +8.19σ peak. The presence of such a bright peak, present in all NCS copies in the unit cell, strongly suggests to us that this site is occupied by an unmodelled atom. The buffers used suggest that this could be either K, Na or Cl, and the neighboring presence of Arg249 suggests that it could be Cl-. However, the authors would know better. Is this a known ion-binding site in HisFH? This issue is more minor as it is unlikely to affect biological interpretations in this paper based on the fact that the site in question is quite distal from both of the main ligand binding sites. Still, when the authors re-refine for issue #1 above, they may wish to try refitting these areas as well.

We have reinvestigated the strong density blobs close to the side chains of Lys238 and Arg 249, revealing that the density is not identical in the three HisFH copies within the asymmetric unit. We noticed that these densities shows tail-like features to different extents. Cleary the density is exceeding the density of an ordered solvent molecule but, as this is close to the relative un-structured C-termini of chains A, C and E, the density could also originate from a distal group of one of the side chains that are removed from nearby residues in the structure (see above for the listing of residues and side chains removed) or from any other ligands added to the crystallization medium. Based on the given uncertainty, we left this density uninterpreted. In addition, we would like to mention that ion binding sites have not been observed in any previous HisF/HisH structures.

As a final aside, we still feel that the effects of mutations and activating ligands in WT vs. hC84S backgrounds may not be truly identical, but the paper presents sufficient overall evidence that they are comparable enough to justify the treatment here in, for example, Fig. 3f.

Overall, we feel that additional revisions are required to address these points regarding the crystal structure.

We thank the reviewers again for their careful assessment of our structure and truly appreciate the constructive remarks.

REVIEWERS' COMMENTS

Reviewer #3 (Remarks to the Author):

We thank the authors for responding to our further comments.

For the ProFAR vs. AICAR point, the new Supp. Fig. 15B provides the relevant information clearly. While there are alternative modelling choices for this situation — including a ProFAR ligand with most atoms missing, or with partial occupancy — the authors' choice to model a solo phosphate and some waters is satisfactory, particularly with the additional PDB caveat they have appended. This is vastly better than the previous model with a product bound, which was actively misleading.

Regarding "speculation" about allostery involving the partially disordered ProFAR, what we meant is that chains C/D seem to show that loosely bound ProFAR is not sufficient to activate the complex. This both supports and adds nuance to the authors' central hypothesis that substrate binding to both halves of the complex is required for activation of the complex, by clarifying that tight, well-ordered binding of ProFAR appears to be required.

The responses to our other comments were appropriate.

Overall we are pleased to recommend publication at this stage.

We (still) review non-anonymously,

Daniel Keedy

Blake Riley
